# Lymph node contraction links sex-biased naive CD8^+^ T cell decline to compromised antigen recognition during middle age

**DOI:** 10.1038/s43587-026-01238-4

**Published:** 2026-10-09

**Authors:** Lutz Menzel, Maria Zschummel, Meghan J. O’Melia, Mårten Sandstedt, Hengbo Zhou, Pin-Ji Lei, Lingshan Liu, Hang Lee, Debattama R. Sen, Lance L. Munn, Lena Jonasson, Timothy P. Padera

**Affiliations:** 1 Edwin L. Steele Laboratories, Department of Radiation Oncology, Massachusetts General Hospital, Harvard Medical School, Boston, MA USA; 2 Department of Medicine, Harvard Medical School, Boston, MA USA; 3 Broad Institute of Massachusetts Institute of Technology and Harvard, Cambridge, MA USA; 4 Krantz Family Center for Cancer Research, Massachusetts General Hospital, Harvard Medical School, Charlestown, MA USA; 5 Department of Health, Medicine and Caring Sciences, Linköping University, Linköping, Sweden; 6 Clinical Department of Radiology, Region Östergötland, Linköping, Sweden; 7 Center for Medical Image Science and Visualization (CMIV), Linköping University, Linköping, Sweden; 8 Biostatistics Center, Massachusetts General Hospital, Harvard Medical School, Boston, MA USA; 9 Clinical Department of Cardiology, Region Östergötland, Linköping, Sweden; 10 Present Address: Immunology of Aging, Leibniz Institute on Aging—Fritz Lipmann Institute (FLI), Jena, Germany

**Keywords:** Lymphocytes, Cancer models, Experimental models of disease, Ageing

## Abstract

The abundance of diverse naive CD8^+^ T cell clones is essential for broad protection against infection and cancer, but how sex and aging jointly shape this compartment remains poorly understood. Here, using mouse models with supporting human data, we uncover a sex-biased mechanism of immune aging in which early male-biased depletion of naive CD8^+^ T cells driven by accelerated, antigen-agnostic differentiation into virtual memory cells combines with thymic involution, limiting naive CD8^+^ T cell replenishment. These mechanisms led to more rapid lymph node contraction and reduced local naive T cell clone availability in males, limiting cancer antigen recognition. Therapeutic thymus regeneration via androgen ablation repopulated naive CD8^+^ T cells in lymph nodes, reinvigorated cancer-specific T cell responses and enhanced responsiveness to immune checkpoint blockade in male mice. These findings reveal the impact of sex and age on naive T cell clone abundance in lymph nodes and suggest strategies to restore immune competence in middle-aged men.

## Abstract (web summary)

Menzel and colleagues report that male mice and humans undergo accelerated immune aging, characterized by naive CD8^+^ T cell decline and early thymic involution, which impairs cancer antigen recognition and response to immunotherapy.

## Main

Age is a dominant risk factor for cancer, with individuals over 50 accounting for 90% of cancer-related mortality. Notably, men exhibit nearly twice the incidence and mortality risk compared to women^1^. Although recent work has begun to clarify how cancer-targeting T cell responses are shaped by biological sex^2,3^ or age^4,5^, the intersection of these factors remains incompletely understood. Addressing these gaps may reveal strategies to restore immune competence and improve cancer outcomes across aging populations in a sex-specific manner.

The immune system’s ability to recognize and respond to a wide array of antigens is directly linked to the number of naive T cells and their clonotypic diversity. With age, the naive T cell compartment declines due to progressive thymic involution and ongoing peripheral attrition via differentiation into memory and senescence-prone effector cells^6,7^. The decline in naive T cells has been linked to reduced vaccine responses, impaired tumor surveillance and reduced efficacy of immunotherapies^6,8^. Lymph nodes (LNs) are the primary sites for initiating immune responses to locoregional antigen sources, rendering them essential for effective anticancer immunity^9,10^. A decline in naive T cells reduces their local abundance within LNs, thereby limiting critical encounters between antigen-presenting cells (APCs) and cognate naive T cell clonotypes. These encounters are critical to generating adaptive immune responses, and limiting them reduces antigen-specific adaptive immunity^11^. Recent work has begun to clarify why the numerical availability of naive CD8^+^ T cells in draining LNs is crucial for anticancer immunity. Anticancer immune responses are inherently stochastic and driven by rare, functionally critical T cells. As the size of the naive T cell precursor pool available for priming decreases, the availability of these rare cells to encounter tumor-antigen-bearing APCs and be activated also decreases^10,12^. The primary source of naive T cells is the thymus, whose output is robust early in life through adolescence, establishing a diverse and self-tolerant peripheral T cell repertoire. Although thymic involution has long been considered a feature of aging with limited impact on the adult immune system^13^, recent human data suggest that it has active clinical relevance that may lead to higher long-term risk of mortality and incidence of cancer^14,15^. These data indicate that sustained thymic activity contributes to ongoing immune competence even later in life. How the decline in naive T cells and thymic atrophy throughout life influence the ability of LNs to initiate adaptive anticancer immune responses, and whether and how this process differs by sex, particularly during middle age when cancer incidence increases, remains unclear.

In this study, we bridge this gap by analyzing naive CD8^+^ T cell dynamics across the lifespan in female and male mice, complemented by supporting observations from human datasets. Our data show a more rapid decline in naive CD8^+^ T cell populations in male mice than in female mice, leading to significantly fewer naive CD8^+^ T cells by middle age. Further, our data indicate that thymic involution alone is insufficient to explain the early and sex-biased loss of naive CD8^+^ T cells; instead, peripheral antigen-agnostic differentiation into virtual memory T (T_VM_) cells acts as an amplifying attrition mechanism as thymic output becomes limiting. The differentiation into T_VM_ cells, which are shorter-lived and more prone to senescence^16^, reduces the abundance of unique naive T cell clones in LNs and is associated with impaired cancer recognition. Experimental thymus regeneration in male mice was able to restore the source of naive CD8^+^ T cells and improved cancer-targeted responses in middle-aged, but not aged, male mice. These mechanisms uncover the potential for immune regeneration strategies to mitigate age- and sex-related differential treatment responses to immune checkpoint blockade. Understanding these mechanisms is pivotal for developing novel therapeutic approaches to mitigate immune aging, particularly by addressing sex-specific vulnerabilities in the thymus and naive T cell regeneration^17,18^.

## Results

### Age and sex are critical factors for tumor-draining LN-derived T cell responses to melanoma

**Fig. 1:**
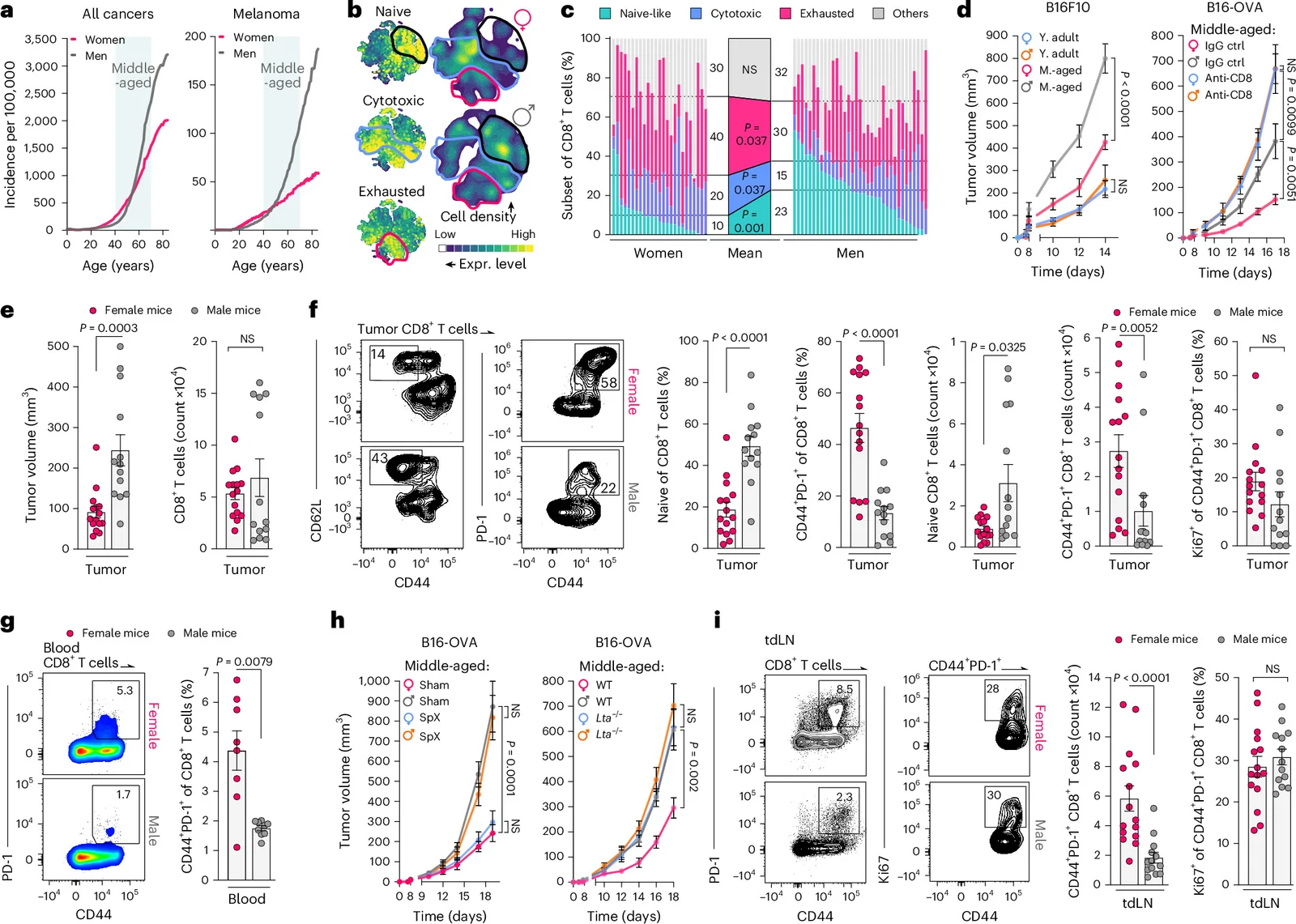
Age and sex are critical factors for tdLN-derived T cell responses to melanoma. **a**, Incidence per 100,000 individuals, based on data from SEER^19^ (*n* = 1.8 × 10^6^–17.8 × 10^6^ cases per age and sex group). **b**, Cell density projections and expression levels of gene module scores of CD8^+^ TILs isolated from melanomas, using integrated public datasets^20–23^. Corresponding data are shown in Extended Data Fig. 1c,d. Expr., expression. **c**, Frequency of CD8^+^ TIL subsets in individual participants and as an average per sex (women, *n* = 25; men, *n* = 35). **d**, B16F10 (left) tumor growth in untreated young adult (Y. adult, *n* = 5) or middle-aged (M.-aged, *n* = 8) mice. B16-OVA (right) tumor growth in middle-aged mice with and without CD8^+^ T cell depletion (*n* = 5 per group). Ctrl, control. **e**, B16-OVA tumor volume and CD8^+^ T cell infiltration 13–15 days after implantation in middle-aged female (*n* = 15) and male (*n* = 13) mice (corresponding to **f** and **g**). **f**, Flow cytometry analysis of CD8^+^ T cells isolated from B16-OVA tumors in middle-aged female (*n* = 15) and male (*n* = 13) mice. The gating strategy is shown in Supplementary Fig. 2. **g**, CD8^+^ T cells from peripheral blood of middle-aged mice (female, *n* = 8; male, *n* = 9) with B16-OVA tumors. **h**, B16-OVA tumor growth in middle-aged mice. Left: after sham surgery or splenectomy (SpX), *n* = 8 per group. Right: in WT or lymphotoxin α-knockout (*Lta*^−/−^) mice, *n* = 8–10 per group. **i**, Flow cytometry analysis of CD8^+^ T cells from the tdLNs of middle-aged mice (female, *n* = 15; male, *n* = 13) with B16-OVA tumors 13–15 days after implantation. The gating strategy is shown in Supplementary Fig. 2. Data are shown as mean ± s.e.m., with each data point representing a single LN or tumor from an individual mouse or person. Statistics were calculated using a two-sided Mann–Whitney *U*-test (MWU; **c**, **e–g** and **i**) and two-way repeated-measures analysis of variance (ANOVA; **d** and **h**) to assess overall effects across time and group. NS, not significant. Source data

**Extended Data Fig. 1:**
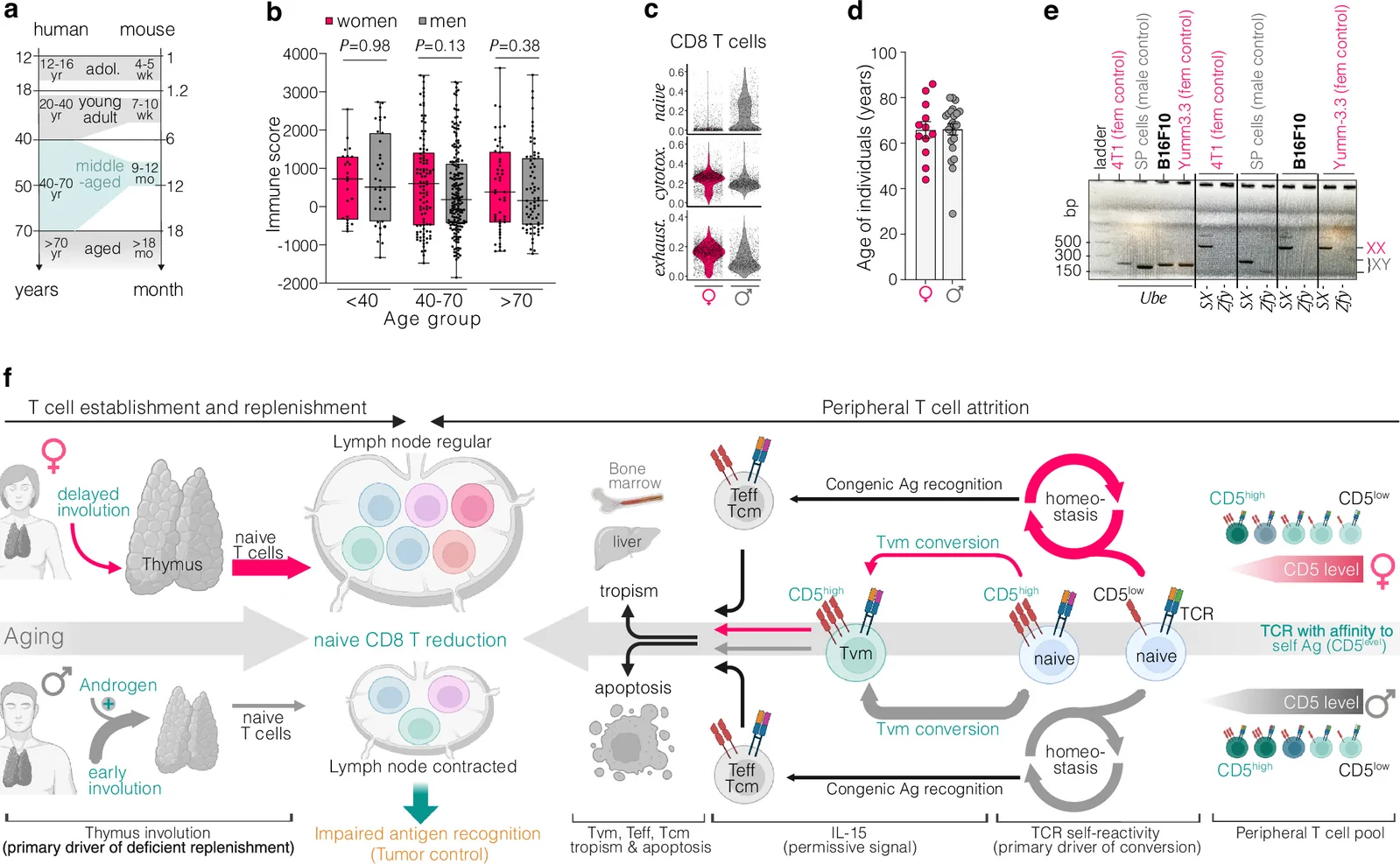
T cell infiltration in melanoma. **a**, Age categories in humans and mice^1,2^. **b**, Immune scores derived from The Cancer Genome Atlas (TCGA) melanoma transcriptomes^3^, reflecting the estimated abundance of tumor-infiltrating immune cells (female/male young adult n = 24/35, middle-aged n = 101/182, aged n = 49/70). Box plots show median, 25^th^-75^th^ percentiles. **c**, Modul score distributions for CD8+T cells from the Durante et al. data set^4^. **d**, Age distribution of melanoma patients included in the analyses shown in Fig. 1b, c (age provided when available; otherwise unspecified). **e**, PCR analysis, *Sly* intron 8 from the Y chromosome and *Xlr* intron 6 from the X chromosome (*SX*, 480/685 bp on XX chromosomes, 280 bp on Y chromosomes). Male control gene *Zfy* and Ubiquitin as general control gene *Uba1/Ube1y1* (Ube, 217 bp). PCR analysis was performed once to characterize the B16F10 melanoma cell line used throughout this study, confirming the absence of the Y chromosome. **f**, Conceptual model linking sex-biased thymic involution and TCR self-reactivity–driven Tvm conversion to naïve CD8^+^ T cell loss, LN contraction, and impaired antigen recognition. Data shown as mean ± SEM with data points representing individual animals. Statistics calculated with two-sided MWU test (b). Illustration in **f** was created with BioRender; Menzel, L. https://biorender.com/w9i0h6x (2026). Source data

Analysis of data from the Surveillance, Epidemiology and End Results (SEER)^19^ program indicates that men have higher overall cancer incidence rates, including melanoma, with these differences becoming apparent in middle age (Fig. 1a and Extended Data Fig. 1a). Both sexes had similar involvement of tumor-infiltrating T cells (TILs) in melanoma across age groups based on transcriptional immune scores (Extended Data Fig. 1b). However, defining T cell differentiation states using transcriptional module scoring of canonical gene signatures (Supplementary Fig. 1a,b) in published samples from individuals with melanoma^20–23^ revealed a higher relative proportion of naive-like CD8^+^ T cells than antigen-experienced (cytotoxic and exhausted) T cells within tumors from men than women (Fig. 1b,c). These data suggest restricted antigen-specific activation in men, which could be a result of deficits in T cell priming in tumor-draining LNs (tdLNs). In addition to well-documented sex- and age-related T cell impairments, such as exhaustion, dysfunction or the development of suppressor phenotypes^2,3,5,24^, a notable proportion of TILs in older individuals retain a naive-like state. Consequently, these TILs may not actively engage in, and could remain bystanders to, antitumor immune responses^25,26^.

**Extended Data Fig. 2:**
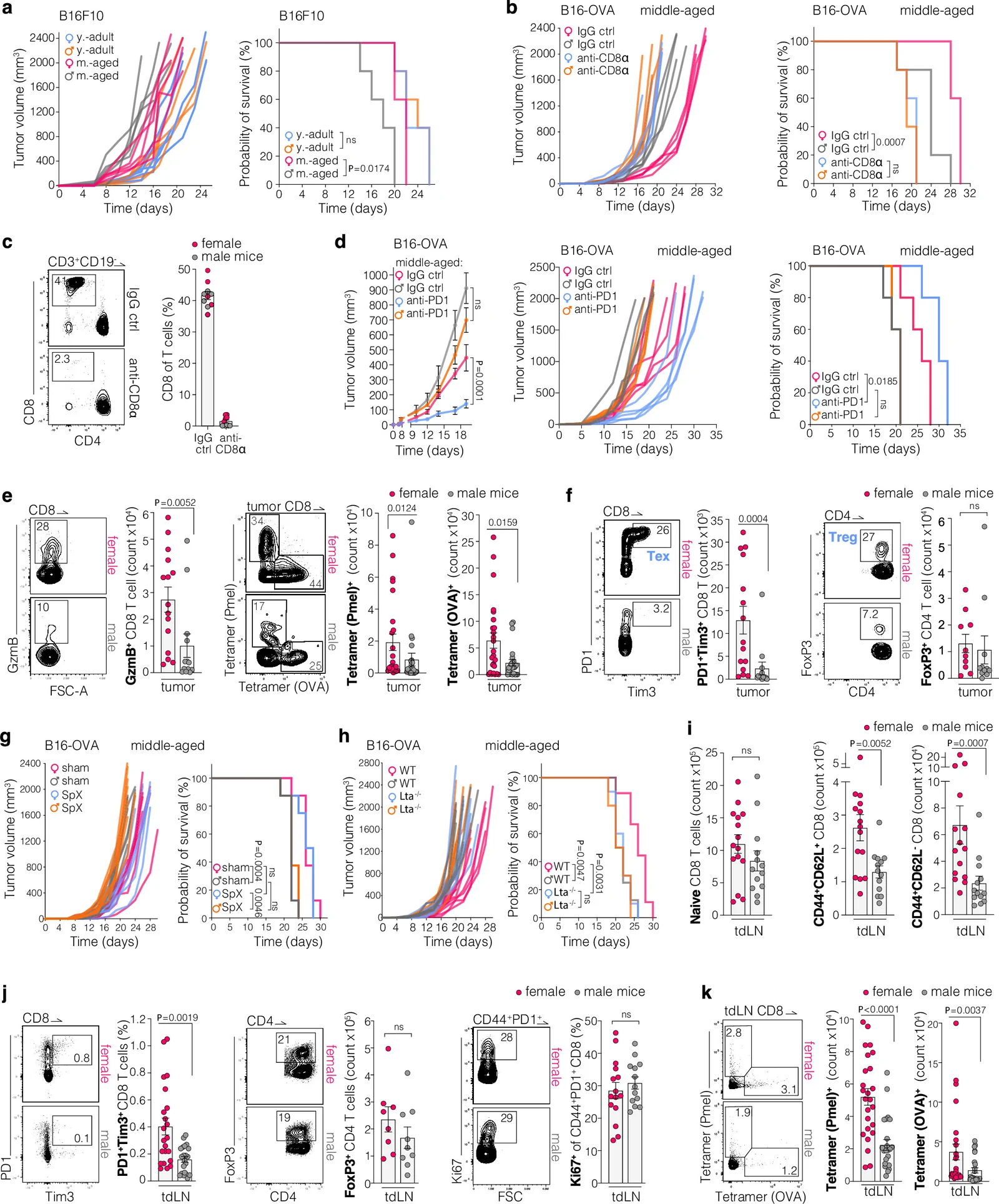
Tumor growth and flow cytometry analysis of the B16 mouse melanoma model. **a**, B16F10 tumor growth and survival probability in adult and middle-aged mice (n = 5-8/group), corresponding to ***left*** part of Fig. 1d. **b**, B16-OVA tumor growth and survival probability in middle-aged mice after CD8+T cell depletion in comparison to controls. **c**, Flow cytometry of PBMCs from mice after treatment with IgG control or anti-CD8+antibody to deplete CD8+T cells, corresponding to Extended Data Fig. 2b and Fig. 1d. **d**, B16-OVA tumor growth and survival probability in middle-aged mice treated with IgG controls or anti-PD1 antibody (n = 5/group). **e** and **f**, Flow cytometry analysis of GzmB^+^, PD1^+^Tim3^+^ (female n = 15, male=13), Tetramer^+^ (female n = 25, male=24), CD8+T cells and FoxP3^+^ CD4 T cells (female n = 10, male=11) from the primary tumor of middle-aged mice implanted with B16-OVA tumors, group matched 13-15 days after implantation (corresponding to Fig. 1e,f,g,i). Data points include the control sham/vehicle groups of middle-aged mice from thymectomy, gonadectomy and Degarelix treatment experiments (Extended Data Fig. 8i, Extended Data Fig. 10c,g). **g**, B16-OVA tumor growth and survival probability in middle-aged mice after sham surgery or splenectomy (SpX), n = 8/group. **h**, B16-OVA tumor growth in middle-aged wild type (WT) or *Lta*^−/−^ mice (n = 8-10/group). **i** and **j**, Flow cytometry analysis of CD8+T cells (female n = 15, male=13) and FoxP3^+^ CD4 T cells (female n = 8, male=9) in the tdLN of middle-aged mice implanted with B16-OVA tumors, group matched 13-15 days after implantation (corresponding to Fig. 1e,f,g,i)**. k**, Flow cytometry analysis of tetramer staining of CD8+T cells from B16-OVA tumor draining LNs of middle-aged mice (female n = 25, male=24). Data points include the control sham/vehicle groups of middle-aged mice from thymectomy, gonadectomy and Degarelix treatment experiments (Extended Data Fig. 8i, Extended Data Fig. 10c,g). Data shown as mean ± SEM with data points representing individual animals. Statistics calculated with two-sided MWU test (e,f,j), two-sided Log-rank test (a,b,d,g,h), 2-way ANOVA (d), ns=nonsignificant. Source data

To assess CD8^+^ T cell-mediated tumor control across age and sex, we compared tumor growth in young adult (7–10 weeks) and middle-aged (9–12 months) mice (Extended Data Fig. 1a) using the B16F10 melanoma model. In young adults, male and female mice showed comparable tumor growth. By middle age, however, male mice displayed a marked loss of tumor control, whereas female mice retained substantially lower tumor growth (Fig. 1d). CD8^+^ depletion in middle-aged mice using the ovalbumin-expressing B16F10 (B16-OVA) melanoma model, which enables tracking of antigen-specific CD8^+^ T cell responses, accelerated tumor growth in both sexes and abolished all sex-related differences, demonstrating that the sex-dependent difference in tumor control is CD8^+^ T cell dependent (Fig. 1d and Extended Data Fig. 2a–c). Consistent with this, middle-aged female mice mounted a more robust response to anti-PD-1 therapy than middle-aged male mice (Extended Data Fig. 2d). At an early stage of tumor growth, when significant sex-related differences in tumor size were already apparent (Fig. 1e, left), we performed flow cytometry analysis of TILs in B16-OVA tumors from middle-aged mice. Consistent with human data (Fig. 1b,c), tumors in middle-aged female mice contained a higher number of antigen-experienced (CD44^+^PD-1^+^), tetramer^+^ T cells and T cells with a functional effector phenotype (granzyme B^+^), while male mice exhibited a predominance of naive CD8^+^ T cells (Fig. 1f and Extended Data Fig. 2e). This disparity occurred despite comparable overall CD8^+^ T cell infiltration and intratumoral proliferation of effector T cells (Fig. 1e,f). The number of exhausted T cells (TIM3^+^PD-1^+^) was higher in female mice, and regulatory T cells were similar between sexes (Extended Data Fig. 2f). The relative increase in naive T cells in tumors from middle-aged male mice suggests that they are not properly primed before entering the tumor. The ability of naive T cells to enter the primary tumor is likely a result of abnormal tumor vasculature, including dysregulation of adhesion molecules on the endothelial cells, leading to aberrant lymphocyte trafficking^27^.

A higher frequency of CD44^+^PD-1^+^ CD8^+^ T cells was also present in the blood of middle-aged female mice, suggesting that these cells are activated outside the tumor and likely reflect more effective priming in LNs (Fig. 1g). In middle-aged male mice, we performed splenectomy or used lymphotoxin α-deficient (*Lta*^−/−^) middle-aged mice, which lack functional LNs^28^, and found that LNs, rather than the spleen, were critical for supporting the superior tumor recognition capacity in middle-aged female mice (Fig. 1h and Extended Data Fig. 2g,h). Consistently, tdLNs from middle-aged female mice contained significantly higher numbers of antigen-experienced (CD44^+^PD-1^+^) and tumor-specific (tetramer^+^) CD8^+^ T cells, independent of the proliferation capacity of activated cells (Fig. 1i and Extended Data Fig. 2i–k), suggesting a higher probability of naive T cells that recognize tumor antigens in the tdLNs of middle-aged female mice. Together, these findings indicate that tumors in middle-aged female mice have a higher proportion of cancer-targeting CD8^+^ effector T cells that are primed in tdLNs before they infiltrate the tumor, consistent with the human data (Fig. 1b,c).

**Extended Data Fig. 3:**
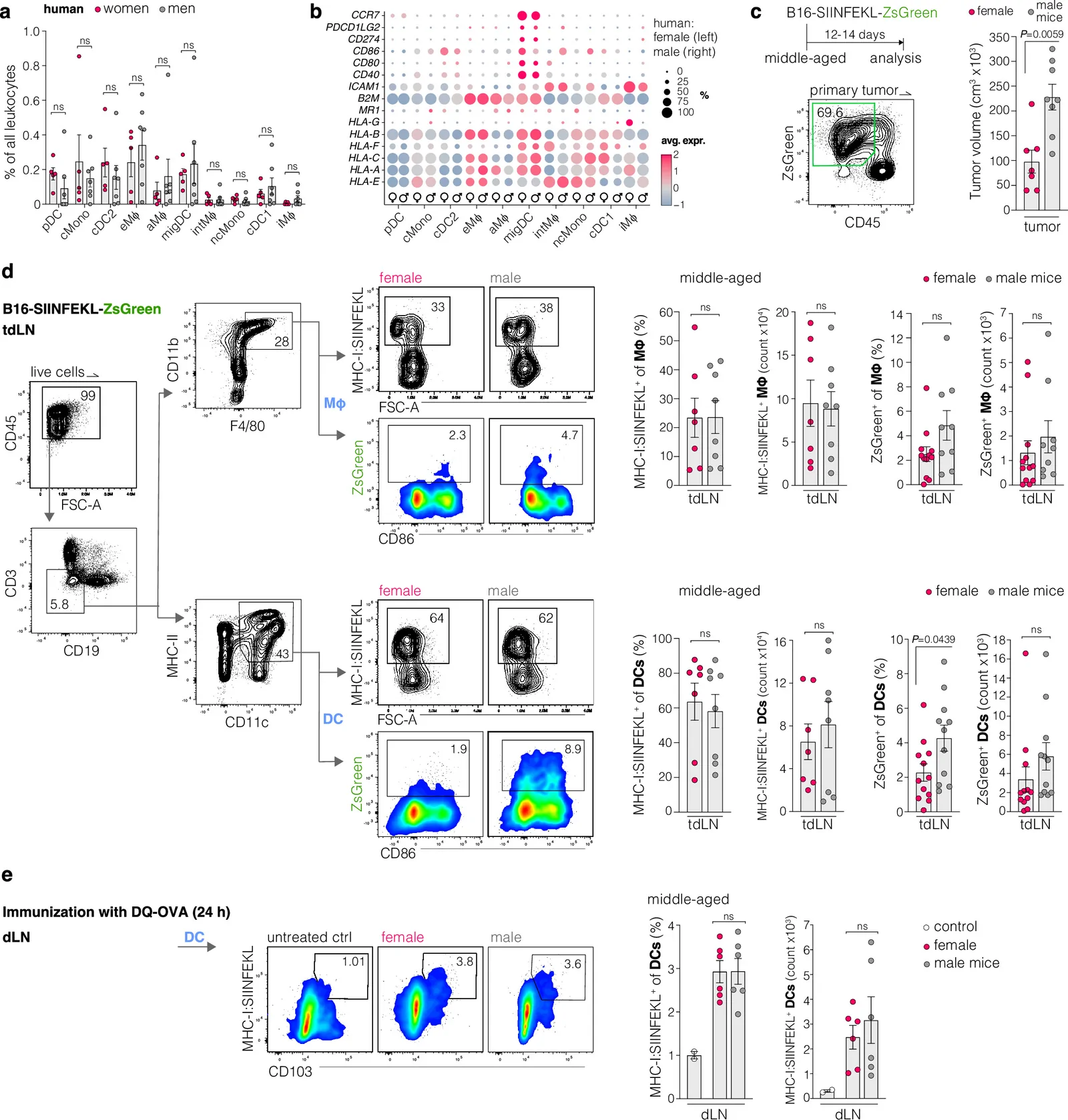
Subset distribution of APCs in human LNs and antigen presentation capacity in murine LNs. **a**, Frequencies of subsets of APC in human LNs (53-73 years of age; women n = 5, men n = 7) taken from scRNA-seq data from Dominguez Conde et al.^8^ Plasmacytoid dendritic cell (pDC), classical monocytes (cMono), classical DC type 2 (cDC2), erythrophagocytic macrophage (eMΦ), alveolar (aMΦ), migratory DC (migDC), intestinal MΦ (intMΦ), non-classical Monocyte (ncMono), classical DC type 1 (cDC1), intermediate MΦ (iMΦ). **b**, Expression levels of functional receptors in APCs of human LNs (women n = 5, men n = 7). **c**, B16-SIINFEKL-zsGreen tumors implanted in middle-aged mice (female n = 7, male=8), volume at day of analysis and representative flow cytometry plot demonstrating presence of zsGreen in tumor cells and in some CD45^+^ leukocytes within the primary tumor. **d**, Representative plots of the flow cytometry analysis and quantification of DCs and MΦ presenting SIINFEKL peptide via MHC-I (MHC-I:SIINFEKL; female n = 7 male n = 8) or carrying tumor antigen (zsGreen; female n = 12, male=9) in tdLNs of middle-aged mice with B16-SIINFEKL-zsGreen tumors. **e**, Representative plots of the flow cytometry analysis and quantification of MHC-I:SIINFEKL^+^ in draining LNs (dLN) of middle-aged mice 25 hours after immunization with DQ-OVA, which identifies antigen uptake and processing (controls n = 2, female n = 6, male n = 6). Data shown as mean ± SEM with data points representing individual animals or persons. Statistics calculated with two-sided MWU test (a,c,d,e), ns=nonsignificant. Source data

Of note, although sex disparities of intradermal APCs have been reported for adult mice^29^, LNs in middle-aged humans did not contain dysregulated proportions of APCs (Extended Data Fig. 3a). Further, no differences in antigen uptake or presentation between the sexes in middle-aged mice were observed, as assessed using the B16-SIINFEKL-zsGreen tumor model and DQ-OVA immunization (Extended Data Fig. 3b–e). The lack of sex-based differences in antigen presentation in LNs prompted us to test whether middle-aged male mice possess a restricted naive T cell pool and, consequently, contain limited numbers of unique T cell antigen receptors (TCRs) capable of recognizing tumor antigens in LNs.

### Age-related decline of naive CD8^+^ T cells and contraction of LNs occur earlier in men and male mice

**Fig. 2:**
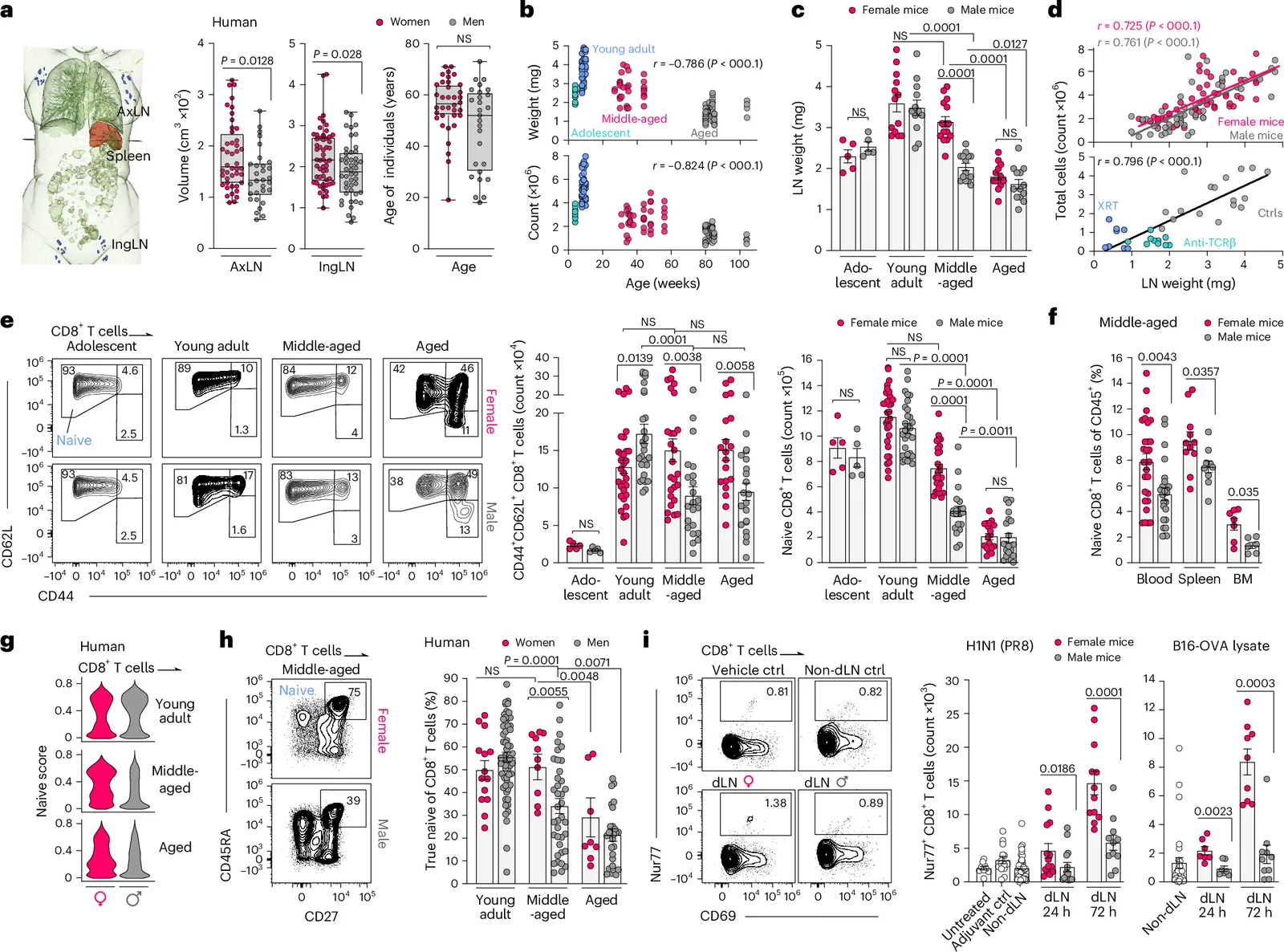
Age-related naive CD8^+^ T cell decline and LN contraction occur earlier in male mice and men. **a**, Average axillary LN (axLN; women, *n* = 54; men, *n* = 46) and ingLN (women, *n* = 44; men, *n* = 30) volume per individual human, segmented from computed tomography scans^30,31^; participant age (when available; for others, age is unspecified). Box plots show median and 25th–75th percentiles. **b**, Correlation of individual ingLN weight (top) or cellularity (bottom) with age in female and male mice. Adolescent mice are shown but excluded from correlation calculations. **c**, LN weights in age groups of female/male mice (adolescents, *n* = 5/5; young adults, *n* = 13/12; middle-aged, *n* = 15/14; aged, *n* = 13/13). **d**, Top: correlation of LN weight with LN cellularity across all ages. Bottom: correlation after irradiation (XRT; 4 Gy) or after T cell depletion with anti-TCRβ. **e**, Flow cytometry analysis of naive (CD44^−^CD62L^+^) and memory (CD44^+^CD62L^+^) CD8^+^ T cells from the ingLNs of mice (adolescents, *n* = 5/5; young adults, *n* = 31/29; middle-aged, *n* = 30/26; aged, *n* = 19/21). The gating strategy is shown in Supplementary Fig. 4. **f**, Frequency of naive CD8^+^ T cells in middle-aged female/male mice in the blood (*n* = 28/25), spleen (*n* = 11/15) and bone marrow (BM; *n* = 7/6). **g**, Expression levels of naive T cell genes (Naive score) in human CD8^+^ T cells from the ABF300 study^35^. The Naive score is described in Supplementary Fig. 1. **h**, Flow cytometry quantification of true naive CD8^+^ T cells from PBMCs of women/men (young adults, *n* = 14/60; middle-aged, *n* = 10/38; aged, *n* = 8/27) using a public dataset^36^. The gating strategy is shown in Supplementary Fig. 5. **i**, Nur77 expression in CD8^+^ T cells from the draining LN (dLN) of middle-aged female/male mice 24 or 72 h after immunization with UV light-inactivated H1N1 (PR8; *n* = 14; 24 h, *n* = 14/14; 72 h, *n* = 12/11; ctrl, *n* = 9; vehicle ctrl, LN, *n* = 44) influenza virus or B16F10-OVA melanoma cell lysate (24 h, *n* = 7/7; 72 h, *n* = 9/10; ctrl, *n* = 32). Data are shown as mean ± s.e.m., with data points representing a single LN from an individual mouse or human participant. Statistics were calculated using the two-sided MWU (**a**, **c**, **e**, **f**, **h** and **i**) or two-sided Spearman correlation (**b** and **d**), and trend lines are shown for visualization (**d**). Source data

**Extended Data Fig. 4:**
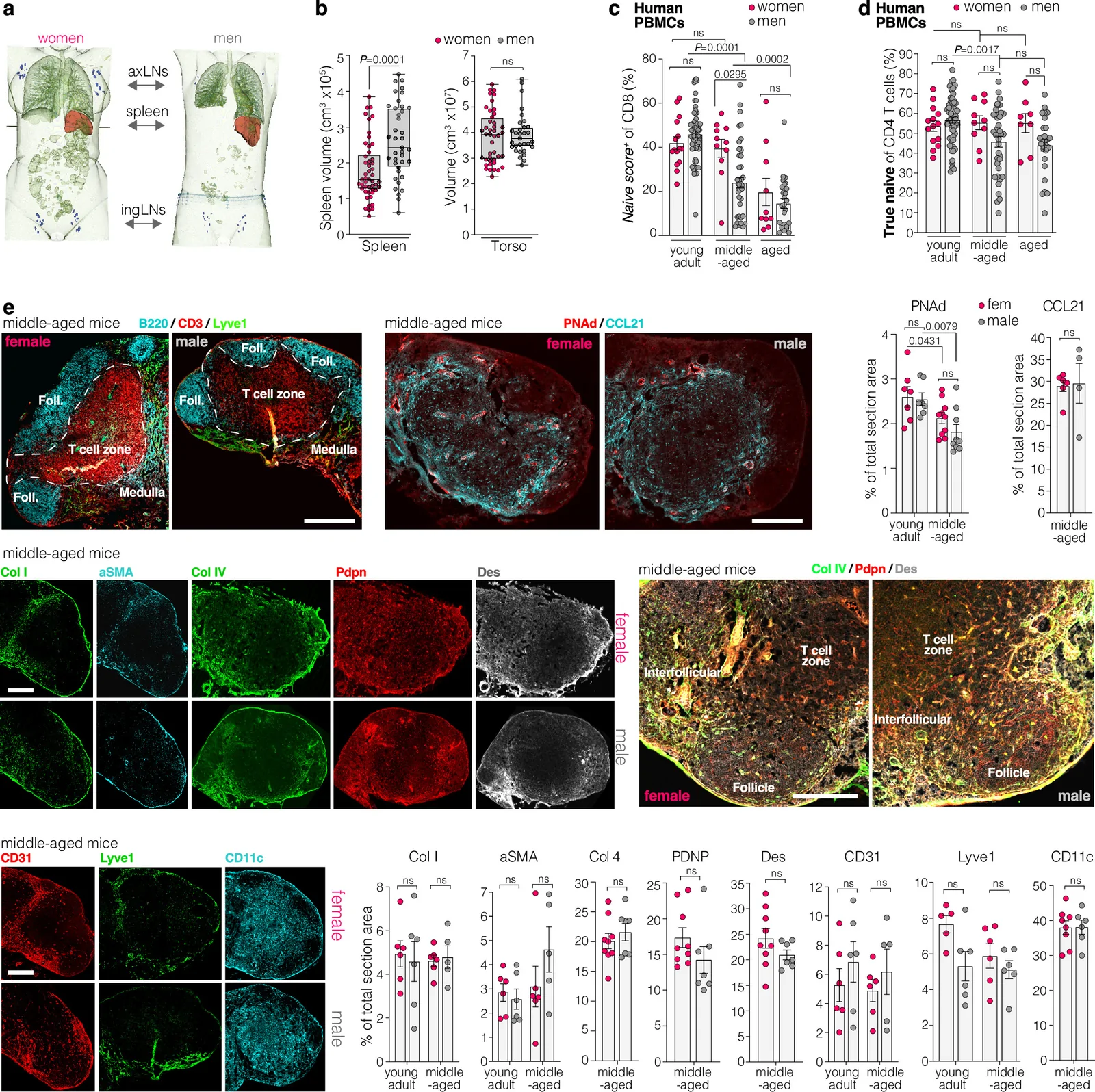
Human LN volumes and naive CD8+T cells. **a**, Schematic representation of LN segmentation, 3D rendering, and quantification of LN volumes based on CT scans from Roth et al. and Rister et al.^9,10^. **b**, Data corresponding to Fig. 2a,b, Segmented and quantified spleen (women/men n = 49/39) and torso volumes (women/men n = 43/31). Box plots show median, 25^th^-75^th^ percentiles. **c**, Quantification of naive CD8+T cells in human PBMCs (women/men young adult n = 15/62; middle-aged n = 11/41; aged n = 10/27) using the *Naive Score*, based on scRNA-seq data from Terekhova et al.^11^. **d**, Flow cytometry quantification of true naive CD4 T cells from human PBMCs, public data set^12^. Gating strategy in Supplementary Data Fig. 5. **e**, Representative images and quantification of inguinal LN sections; B220 (B cells), CD3 (T cells), peripheral node adressin (PNAd, high endothelial venules; female/male young adult n = 7/7, middle-aged n = 10/9), C-C motif chemokine ligand 21 (CCL21; female/male n = 6/4), collagen I (Col 1; female/male young adult n = 6/6, middle-aged n = 6/5), alpha smooth muscle actin (αSMA; female/male young adult n = 6/6, middle-aged n = 6/5), Collagen IV (Col 4; female/male n = 9/7, podoplanin (Pdpn; n = 9/7), Desmin (Des; female/male n = 9/7), CD31 (PECAM-1, endothelial cells; female/male young adult n = 6/6, middle-aged n = 6/5), lymphatic vessel endothelial hyaluronan receptor-1 (Lyve-1, lymphatic endothelial cells; female/male young adult n = 5/6, middle-aged n = 6/6), CD11c (dendritic cells; female/male n = 8/6). Scale bar: 200 µm, 50 µm (FRC network images). Data shown as mean ± SEM with data points representing individual animals or human subjects. Statistics calculated with two-sided MWU test (b,c,d,e), ns=nonsignificant. Source data

Volume segmentation of full-body computed tomography scans of humans from a publicly available database^30,31^ revealed larger spleens but significantly smaller LNs in men than in women (Fig. 2a and Extended Data Fig. 4a,b). With age, LNs can accumulate fat due to lipomatosis, which can disrupt the correlation between volume and cellularity, potentially leading to an underestimation of the decline in cellularity^32^. In middle-aged mice, LNs did not show a dysregulated stromal structure (Extended Data Fig. 4e). Consistent with human LN data, mouse LN size and cellularity declined with age, occurring earlier and more markedly in male mice and resulting in a significant sex disparity by middle age (Fig. 2b,c). The differences were more pronounced in skin-draining LNs than in mesenteric LNs and were independent of reproductive status and genetic background (Supplementary Fig. 3a–c). LN weight strongly correlated with cellularity during physiological aging and under lymphopenic conditions, emphasizing the codependence of LN size with the number of lymphocytes (Fig. 2d and Supplementary Fig. 3d).

Previous studies have established that the naive CD8^+^ T cell compartment undergoes substantial remodeling with age, including numerical reductions in naive CD8^+^ T cells^33,34^. Much of this decline has traditionally been viewed in the context of a proportional shift from naive to memory phenotypes^6^. However, our data showed quantitative stability or loss of CD44^+^CD62L^+^ memory-phenotype cells and a clear loss of naive CD8^+^ T cells in inguinal LNs (ingLNs) beginning shortly after young adulthood, with naive cells exhibiting a more pronounced reduction in male mice than in female mice. Both sexes converged with advanced age (Fig. 2e). This decline in naive CD8^+^ T cells was also evident in blood, spleen and bone marrow (Fig. 2f), underscoring a systemic loss of naive CD8^+^ T cells with advancing age. Analysis of published multimodal datasets of human T cells from blood^35,36^ corroborated these findings, showing a decline in naive CD8^+^ T cells determined by flow cytometry or by applying a gene expression ‘Naive score’ on single-cell RNA-sequencing (scRNA-seq) data (Fig. 2g,h and Extended Data Fig. 4c).

**Extended Data Fig. 5:**
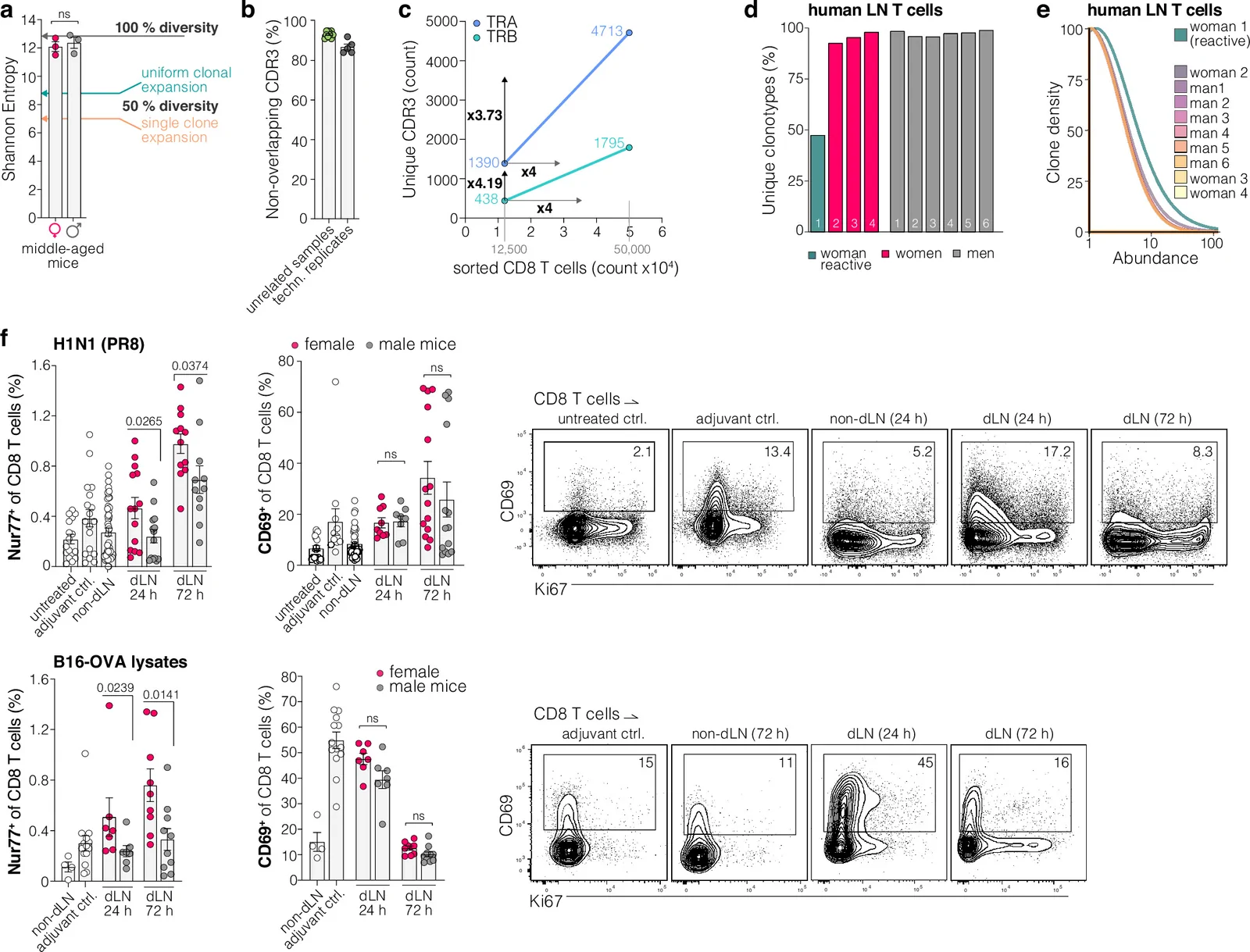
TCR diversity of naive T cells in LNs and T cell activation after immunization in mice. **a**, Shannon Entropy analysis of the TCR repertoire (T cell receptor beta chain, TRB) diversity using bulk TCR sequencing data of CD8+T cells sorted from inguinal LNs of middle-aged mice (n = 3/sex). Indicated are approximations of theoretical values with 100% diversity and 50% diversity in reactive LNs with an even clonal expansion or a single expanded clone. **b**, CDR3 (TRB) sequences overlapping with other samples (n = 10) or technical replicates (n = 5) of the same sample. **c**, Bulk TCR sequencing of T cells from the same LN sample (ingLN of middle-aged male) with different CD8+T cell counts (12,500 and 50,000 cells). Indicated are unique CDR3 (TRA, TRB) and the fold change (x…) between samples with different input cell counts. Results show that almost every additional cell in this LN sample equals an additional unique TCR. **d**, scTCR-seq of T cells from human LNs, data set from Dominguez Conde et al.^8^ Percent of unique clonotypes (including combination of TRA, TRB). One female sample with apparent reactive LN (see **e**). **e**, Clonal density and abundance (arbitrary units) of samples depicted in **d**. **f**, Flow cytometry analysis of Nur77 and CD69 expression in CD8+T cells 24 or 72 hours after immunization with inactivated H1N1 (PR8) virus particles (untreated controls n = 17, vehicle n = 12, contralateral LN n = 45; female/male 24 h n = 9/8, 72 h n = 14/14) or B16-OVA tumor cell lysates (vehicle n = 4, contralateral LN n = 19; female/male 24 h n = 7/7, 72 h n = 9/10). Data shown as mean ± SEM with data points representing single LNs of individual animals or persons. Statistics calculated with t test (a), two-sided MWU test (f), ns=nonsignificant. Source data

To examine TCR diversity in LNs, we performed TCR sequencing of CD8^+^ T cells from LNs of middle-aged mice and found a maximally diverse TCR repertoire with over 96% of unique T cell clonotypes in non-reactive LNs independent of sex, mirrored by observations in humans (Extended Data Fig. 5a–e). Thus, we hypothesized that the absolute number of naive T cells within an LN at any given time is directly correlated with the number of unique TCRs present and is therefore a rate-limiting factor for the probability of antigen recognition. To test this, we immunized middle-aged mice intradermally in the flank with either inactivated H1N1 influenza virus particles or B16-OVA tumor cell lysates and analyzed the draining LNs at 24 and 72 h after immunization to assess early T cell activation. Both sexes exhibited similar frequencies of CD8^+^ T cells expressing the general activation marker CD69. However, a significantly greater number of CD8^+^ T cells in the LNs of female mice exhibited TCR-specific activation, as indicated by a higher number of Nur77^+^ cells (Fig. 2i and Extended Data Fig. 5f). These data highlight the functional impact of lower naive T cell numbers and smaller LNs in middle-aged male mice on the generation of antigen-specific immune responses. These findings support a model in which reduced availability of naive CD8^+^ T cells limits the probability of cancer-reactive clones. This is consistent with recent work^10,12^ showing that a diminished naive T cell pool is expected to directly constrain the formation and diversity of these precursor populations, thereby reducing the likelihood of effective cancer-specific T cell responses. The greater activation of CD8^+^ T cells in LNs of middle-aged female mice is consistent with an increase in the proportion of antigen-experienced CD8^+^ TILs in the human data (Fig. 1b,c). Even though middle-aged male mice have fewer naive CD8^+^ T cells in total, they also have fewer antigen-experienced CD8^+^ T cells due to a reduced number of tumor-antigen-cognate T cell clones in LNs. Thus, as a proportion, the TILs in the human data remain skewed toward a naive-like phenotype in men.

### Enhanced propensity for an antigen-agnostic loss of T cell naivety in males

Although thymic involution is a well-known feature of aging, its functional relevance remains debated^13,14^, as peripheral T cell numbers, particularly in the CD4^+^ T cell compartment, remain relatively stable in adulthood^37^ (Extended Data Fig. 4d). However, other studies^33,34,38,39^ and our data reveal a progressive, selective decline in naive CD8^+^ T cells with age, suggesting that peripheral maintenance is insufficient to maintain the CD8^+^ T cell pool once thymic output declines with age. This raises the question of what drives this loss and why it occurs earlier in men.

**Fig. 3:**
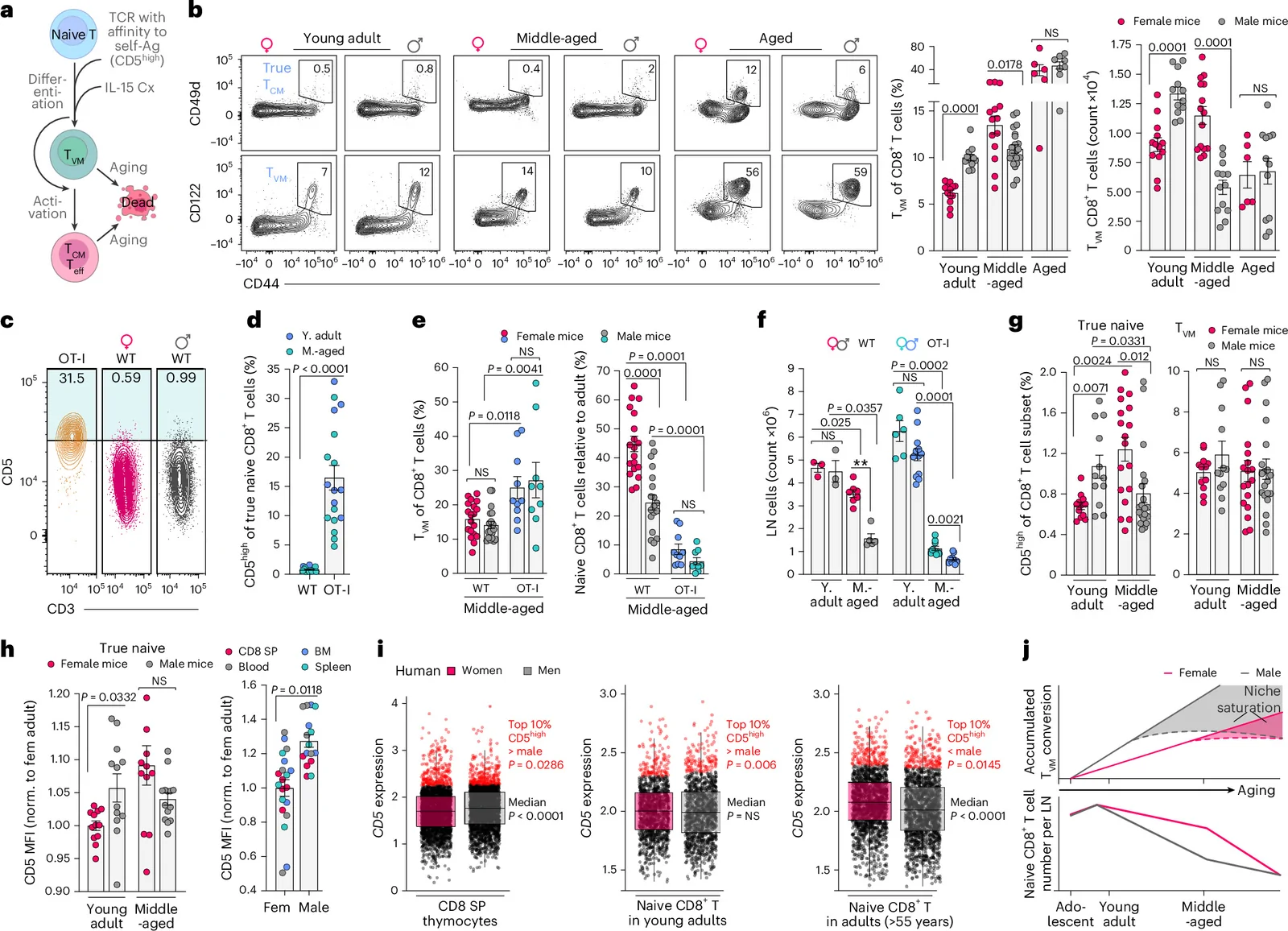
Enhanced propensity for antigen-agnostic loss of T cell naivety in male mice. **a**, Schematic illustration of naive T cell differentiation into T_VM_, effector (T_eff_) and T_CM_ cells. Ag, antigen. **b**, T_CM_ and T_VM_ cells in the ingLNs of WT female/male mice (young adults, *n* = 13/12; middle-aged, *n* = 14/16; aged, *n* = 6/8). The gating strategy is shown in Supplementary Fig. 4. **c**,**d**, Example plots (**c**) and quantification of (**d**) CD5^high^ cells in true naive CD8^+^ T cells from ingLNs of WT (*n* = 18) or OT-I (*n* = 18) mice. OT-I mice were used as a known positive control. **e**, Quantification of T_VM_ and naive CD8^+^ T cells in middle-aged WT (female, *n* = 19; male, *n* = 19) and OT-I (female, *n* = 10; male, *n* = 9) mice. **f**, Quantification of total cell counts of ingLNs of young adults and middle-aged female/male WT (young adults, *n* = 3/3; middle-aged, *n* = 7/5) and OT-I (young adults, *n* = 6/13; middle-aged, *n* = 10/9) mice. **P = 0.0025. **g**, Quantification of CD5^high^ cells from the ingLNs of WT mice as a percentage of true naive CD8^+^ T or T_VM_ cells (female/male mice; young adults, *n* = 12/12; middle-aged, *n* = 18/20). **h**, CD5 mean fluorescence intensity (MFI) normalized to young adult female mice (female/male mice young adults, *n* = 12/12; middle-aged, *n* = 12/14) and MFI of CD5^+^ CD8^+^ single-positive thymocytes and true naive CD8^+^ T cells from the blood, bone marrow and spleen (female, *n* = 5 per tissue; male, *n* = 4 per tissue). Norm., normalized; fem, female. **i**, Human *CD5* expression in top 10% CD5^high^ cells and median overall *CD5* expression in CD8^+^ single-positive thymocytes from young adults (women, *n* = 9; men, *n* = 17; left) or naive CD8^+^ T cells in young adults (women, *n* = 8; men, *n* = 39) or adults over 55 years of age (women, *n* = 16; men, *n* = 51; right). Data are from published datasets of human thymocytes^46,47^ and PBMCs^35^. Box plots show median and 25th–75th percentiles. **j**, Illustrative projection of cumulative conversion of CD5^high^ naive CD8^+^ T cells into T_VM_ cells during aging. Top: linear accumulation of T_VM_ cells over time (solid line) and the assumed deceleration in accumulation due to competition and the prior conversion of naive CD8^+^ T cells with highest CD5 levels (dashed line: slow down). Bottom: the T_VM_ cell formation-associated attrition of naive CD8^+^ T cells. Data are shown as mean ± s.e.m., with each data point representing a single LN or tumor from an individual animal. Statistics were calculated using the two-sided MWU (**b** and **d**–**h**) or the two-sided Wilcoxon rank-sum test (**i**). SP, single-positive. Illustration in **a** created in BioRender; Menzel, L. https://biorender.com/m2eqnj4 (2026). Source data

**Extended Data Fig. 6:**
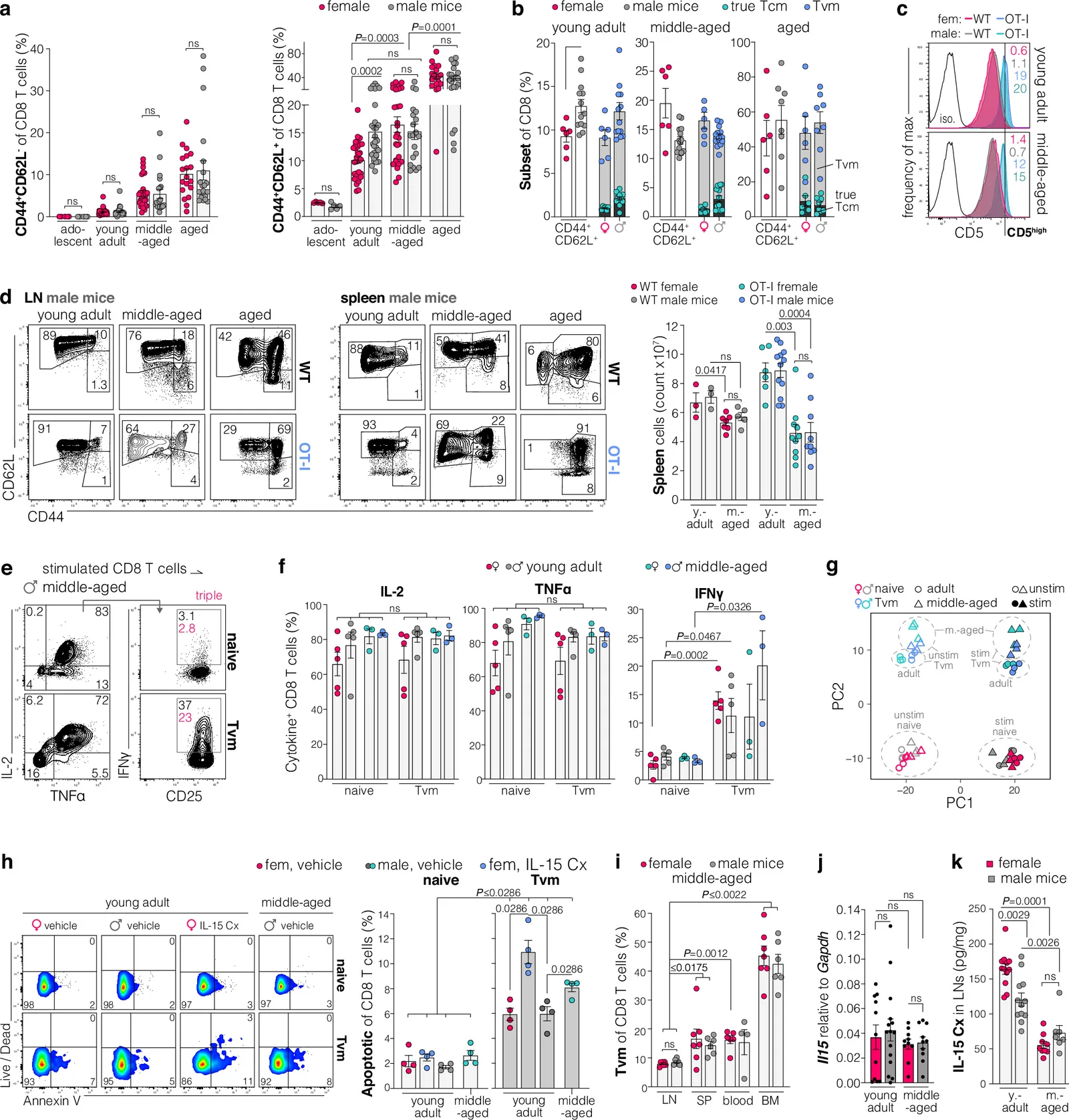
Age group related analysis of naive and memory CD8+T cells in wild type and OT-I mice. **a**, Flow cytometry analysis of central memory-like (CD44^+^CD62L^+^) and effector-like (CD44^+^CD62L^−^) CD8+T cells in LNs of wild-type (WT) mice (female/male adolescent n = 5/5, young adult n = 31/29, middle-aged n = 28/20, aged n = 19/21). **b**, Quantification of flow cytometry analysis of CD8+T cells from the ingLN of adult (female n = 6, male n = 12), middle-aged (female n = 6, male n = 14) and aged (female n = 6, male n = 8) mice show that CD44^+^CD62L^+^ contain both true Tcm and virtual memory (Tvm) cells, with a majority being Tvm. **c**, Histograms of CD5 expression in true naive CD8+T cells, vertical line indicates CD5^high^ cells from ingLNs of WT or OT-I mice, young adults (y. adult) and middle-aged (m.-aged). **d**, Representative flow cytometry plots of CD8+T cells and quantification of total cell count from the spleens of WT (female/male young adult n = 3/3, middle aged n = 7/5) and OT-I mice (female/male young adult n = 6/12, middle aged n = 10/9). **e**, Example plots and **f**, Quantification of cytokine production in subsets of CD8+T cells in vitro following stimulation (female/male young adult n = 5/5, middle-aged n = 3/3). **g**, Principal component (PC) analysis of VST-normalized bulk RNA-seq counts across all 48 samples. **h**, Flow cytometry analysis of Live/Dead (Zombie UV) and Annexin-V staining in true naive or Tvm cells of young adult mice after treatment with vehicle solution or IL-15 Cx or from vehicle treated middle-aged males (n = 4/age and treatment group). Analysis 1 week after end of treatment. **i**, Flow cytometry analysis of Tvm CD8+T cells in LNs (female n = 7, male 6), spleen (SP; female n = 7, male 6), blood (female n = 6, male 4) and bone marrow (BM; female n = 7, male 6). **j**, qPCR gene expression analysis of *Il15* relative to *Gapdh* in murine ingLN (female/male young adult n = 11/15, middle-aged n = 10/10). **k**, ELISA quantification of IL-15/IL-15 receptor alpha complex (Il-15 Cx) in ingLNs of young adult and middle-aged mice, indicated relative to weight of LNs (female/male young adult n = 12/12, middle-aged n = 9/7). Data shown as mean ± SEM with data points representing single LNs or tissues of individual animals. Statistics calculated with Student’s t-test (b,f) or two-sided MWU test (a,d,h,I,j,k), ns=nonsignificant. Source data

Naive CD8^+^ T cells are long-lived but can exit the naive pool through activation into effector and central memory T (T_CM_) cells following antigen encounter or via antigen-agnostic differentiation into T_VM_ cells^40–42^ (Fig. 3a). While the effector/T_CM_ route can be pathogen driven^6,8,43^, our data show that naive CD8^+^ T cells still decline with age in mice housed under low-pathogen conditions (Fig. 2e,f). CD44^+^CD62L^−^ effector-like cells did not differ by sex across the lifespan, suggesting similar antigen exposure. However, young adult male mice exhibited an increased frequency of CD44^+^CD62L^+^ CD8^+^ T cells before any sex difference in LN cellularity was apparent in middle age (Extended Data Fig. 6a). In line with previous studies^16,44^, most CD44^+^CD62L^+^ cells were not true T_CM_ cells (CD44^+^CD49d^+^) but T_VM_ cells (CD44^+^CD122^+^CD49d^−^; Fig. 3b and Extended Data Fig. 6b). The propensity of naive CD8^+^ T cells to undergo antigen-agnostic differentiation into T_VM_ cells is shaped by their clone-specific sensitivity to self-peptide–major histocompatibility complex (MHC) and is reflected by CD5 expression, which is stably imprinted during thymic selection^45^. CD5^high^ cells are more self-reactive and more likely to undergo T_VM_ cell conversion in the periphery over time^40,42^.

To test whether high self-reactivity alone is sufficient to drive this process, we used OT-I mice, in which CD8 T cells express the same transgenic TCR and have elevated reactivity for endogenous self-antigens^40,42^, resulting in higher proportions of CD5^high^ cells than wild-type (WT) mice (Fig. 3c,d and Extended Data Fig. 6c). As expected, OT-I mice exhibited accelerated T_VM_ cell formation, a pronounced reduction of naive CD8^+^ T cells in LNs with age and reduced cellularity of both the LNs and spleen in middle age in both sexes (Fig. 3e,f and Extended Data Fig. 6d). These findings confirm that high TCR self-reactivity is sufficient to drive antigen-agnostic T_VM_ cell differentiation and naive CD8^+^ T cell pool contraction. To determine whether this mechanism operates in a physiologically diverse TCR setting, we analyzed WT mice and found that CD5^high^ cells were enriched in the T_VM_ cell population relative to naive CD8^+^ T cells. CD5^high^ true naive CD8^+^ T cells were more frequent in young adult male mice than in female mice (Fig. 3g), accompanied by higher CD5 expression levels across LNs and other organs (Fig. 3h), suggesting a male-biased predisposition to early T_VM_ cell conversion. This difference reversed with age as CD5^high^ naive CD8^+^ T cell frequencies increased in female mice but declined in middle-aged male mice, likely reflecting prior selective loss of CD5^high^ self-reactive clones through T_VM_ cell differentiation (Fig. 3g). Consistent with the increased frequency of CD5^high^ cells in young male mice, our analysis of publicly available scRNA-seq data^35,46,47^ from human CD8 single-positive thymocytes and naive CD8^+^ T cells in peripheral blood mononuclear cells (PBMCs) showed elevated CD5 expression among the top 10% CD5^high^ cells in men compared to women (Fig. 3i).

Although the difference in CD5^high^ naive CD8^+^ T cell frequency between young adult women and men is modest (approximately 0.7% versus 1.1%), these cells arise from a compartment comprising >85% of all CD8^+^ T cells, resulting in a substantial absolute pool of highly self-reactive clones available for T_VM_ cell conversion. To assess the long-term impact, we generated a simple model of naive-to-T_VM_ cell conversion with sex-specific rates based on the CD5^high^ naive CD8^+^ T cell frequency (Supplementary Fig. 6a–d), showing that small initial differences in CD5^high^ frequency can drive cumulative divergence in T_VM_ cell generation and naive CD8^+^ T cell depletion over time (Fig. 3j).

### Transient experimental T_VM_ cell induction initiates a naive CD8^+^ T cell decline over time

**Fig. 4:**
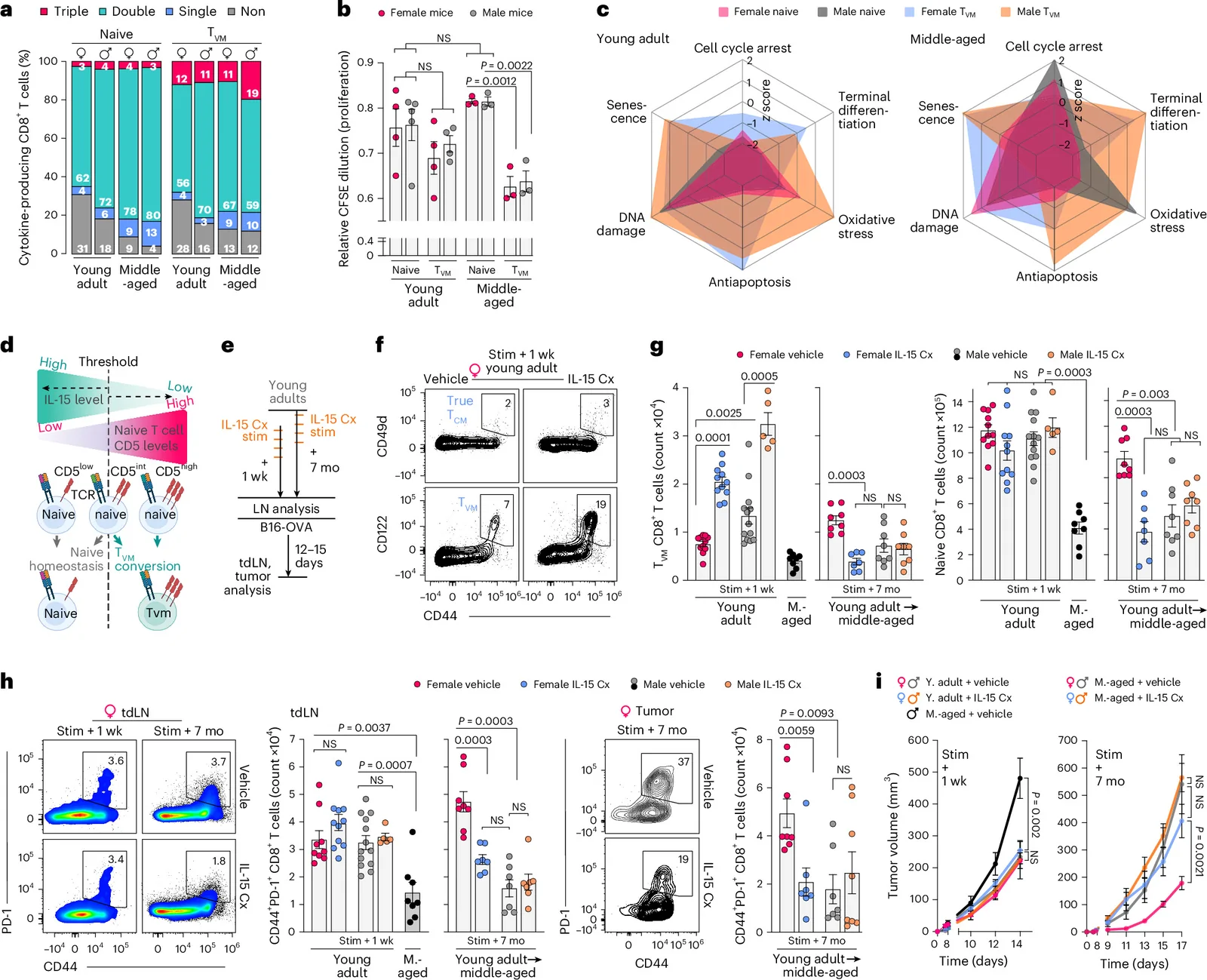
Experimental induction of T_VM_ cells in young female mice leads to restricted naive CD8^+^ T cell pool in middle age. **a**, Polyfunctional cytokine production in subsets of CD8^+^ T cells after in vitro stimulation, showing the proportion of cells producing combinations of IL-2, interferon-γ (IFNγ) and tumor necrosis factor (TNF)) as a measure of effector capacity. **b**, Proliferation capacity of CD8^+^ T cell subsets after in vitro stimulation, with quantification depicted by dilution of carboxyfluorescein succinimidyl ester (CFSE) MFI relative to unstimulated controls (female/male mice; young adult, naive: *n* = 4/5; young adult, T_VM_ cells: *n* = 4/4; middle-aged, naive: *n* = 3/3; middle-aged, T_VM_ cells: *n* = 3/3) using 1 independent experiment. **c**, Radar plots showing *z*-scored mean expression of senescence-associated gene sets in young adult and middle-aged naive and T_VM_ CD8^+^ T cells across all groups (average of *n* = 3 per group). Gene sets are described in Methods. **d**, Schematic model of naive-to-T_VM_ cell conversion. IL-15 acts as a permissive signal that establishes a self-reactivity threshold (CD5 level, here depicted as low, intermediate (int) or high). Only naive CD8^+^ T cells above this threshold undergo conversion to T_VM_ cells. **e**, Experimental setup. Young adult and middle-aged mice were treated with vehicle or IL-15 Cx (once weekly for 4 weeks) with LN analysis and B16-OVA tumor implantation 1 week after the acute stimulation phase (stim + 1 wk) or 7 months later (stim + 7 mo). **f**, Representative flow cytometry plots. **g**, Quantification of CD8^+^ T cells in the ingLNs of mice treated with vehicle plus 1 week (female/male adult, *n* = 11/13; middle-aged male, *n* = 8) and after 7 months (female/male adult, *n* = 8/8) or IL-15 Cx plus 1 week (female/male adult, *n* = 11/5) and after 7 months (female/male, *n* = 7/8), as described in **f**. **h**, Flow cytometry analysis of CD8^+^ T cells from the tdLNs or the primary tumor. Young adult and middle-aged mice were treated with vehicle and analyzed at 1 week (female/male, young adult, *n* = 9/13; middle-aged male, *n* = 8) or after 7 months (female/male, young adult, *n* = 8/7) or treated with IL-15 Cx and analyzed at 1 week (female/male, young adult, *n* = 11/5) or after 7 months (female/male, *n* = 7/7). **i**, B16-OVA tumor growth in mice treated with vehicle plus 1 week (female/male, young adult, *n* = 10/11; middle-aged male, *n* = 8) or IL-15 Cx plus 1 week (female/male, young adult, *n* = 11/5) and treatment with vehicle plus 7 months (female, *n* = 8; male, *n* = 7) or IL-15 Cx plus 7 months (female, *n* = 8; male, *n* = 8), as described in **e**. Data are shown as mean ± s.e.m., with each data point representing a single LN or tumor from an individual animal. Statistics were calculated using the unpaired two-sided *t-*test (**b**), two-sided MWU (**g** and **h**) and two-way repeated measures ANOVA (**j**). Illustration in **d** created in BioRender; Menzel, L. https://biorender.com/tsxp0ok (2026). Source data

From an evolutionary perspective, the early skimming of naive CD8^+^ T cells to T_VM_ cells may reflect a functional trade-off. T_VM_ cells offer rapid, cytokine-driven responses that prioritize short-term immune readiness over long-term repertoire maintenance^40,48^. Compared to naive cells, T_VM_ cells displayed enhanced effector cytokine production following stimulation, yet lost proliferative capacity, upregulated senescence-associated transcriptional signatures (Fig. 4a–c and Extended Data Fig. 6e–g) and increased apoptosis rates with age (Extended Data Fig. 6h), with minimal age- and sex-dependent metabolic differences (Supplementary Fig. 7). LNs continued to shrink with age despite T_VM_ cell formation, as T_VM_ cells have a shorter lifespan due to increased apoptosis^16^ and preferentially relocate to the spleen, bone marrow (Extended Data Fig. 6i) or liver^40^. Both of these processes contribute to a sex-biased loss of naive CD8^+^ T cells and LN contraction earlier in life in men.

To further establish that T_VM_ cell formation can lead to depletion of naive CD8^+^ T cell populations, we used interleukin-15 (IL-15)/IL-15 receptor-α complexes (IL-15 Cx), the biologically most active form of IL-15, to experimentally drive T_VM_ cell formation in young adult mice. As illustrated in Fig. 4d, IL-15 did not directly induce T_VM_ cell differentiation but acted as a permissive costimulatory signal that lowered the activation threshold of self-reactivity (CD5^level^) of naive CD8^+^ T cells for conversion into T_VM_ cells^40,49^. Thus, IL-15 Cx enables experimental lowering of the self-affinity threshold for T_VM_ cell formation in a setting with preserved TCR diversity and low baseline conversion.

Recombinant IL-15 Cx was administered once weekly for four weeks to young adult mice (Fig. 4e). IL-15 Cx-treated female mice allowed us to ask whether transient, experimentally induced T_VM_ cell formation could mimic male-like immune aging. This approach allowed us to probe the causal link between premature T_VM_ cell skewing and later-life naive T cell loss in a setting where these features are not inherently coupled, thereby isolating the hypothesized process from confounding differences. We observed a marked expansion of T_VM_ cells in LNs of young adult female mice after the acute IL-15 Cx stimulation phase (stimulation + 3 weeks). Remarkably, this transient intervention in female mice led to a significant and lasting reduction in naive CD8^+^ T cells 7 months later (stimulation + 7 months), mimicking the profile observed in age-matched untreated male mice (Fig. 4f,g). As T_VM_ cells are more prone to apoptosis and preferentially leave LNs for other organs, their accelerated conversion from naive CD8^+^ T cells provides an explanation for the sustained depletion of CD8^+^ T cell populations observed after IL-15 Cx treatment. We next assessed whether endogenous IL-15 levels differ between sexes. *Il15* transcript levels were comparable at all ages, whereas protein levels showed a modest decrease only in young male mice (Extended Data Fig. 6j,k). As the transcription of *Il15* is not different in young male and female mice, the reduction in IL-15 in young male mice could result from increased IL-15 consumption by the expanding T_VM_ cell compartment. Further, these data indicate that sex-specific differences in IL-15 availability are unlikely to underlie the observed phenotype.

Our data show that this transient expansion of the T_VM_ cell pool was sufficient to drive a long-lasting reduction in naive CD8^+^ T cell numbers.

Although the acute expansion of T_VM_ cells had no measurable effect on tumor rejection shortly after treatment (stimulation + 3 weeks), early-life IL-15 Cx treatment led to decreased numbers of activated CD8^+^ T cells within tdLNs and the tumor and reduced tumor control in middle-aged female mice (Fig. 4h,i). These findings demonstrate that transient induction of T_VM_ cells in young adult female mice preserves short-term antitumor effector function but causes loss of long-term tumor control through naive CD8^+^ T cell attrition, thereby mimicking key features of male-biased immune aging.

### Early thymic involution in males limits naive CD8^+^ T cell replenishment and facilitates LN contraction

**Fig. 5:**
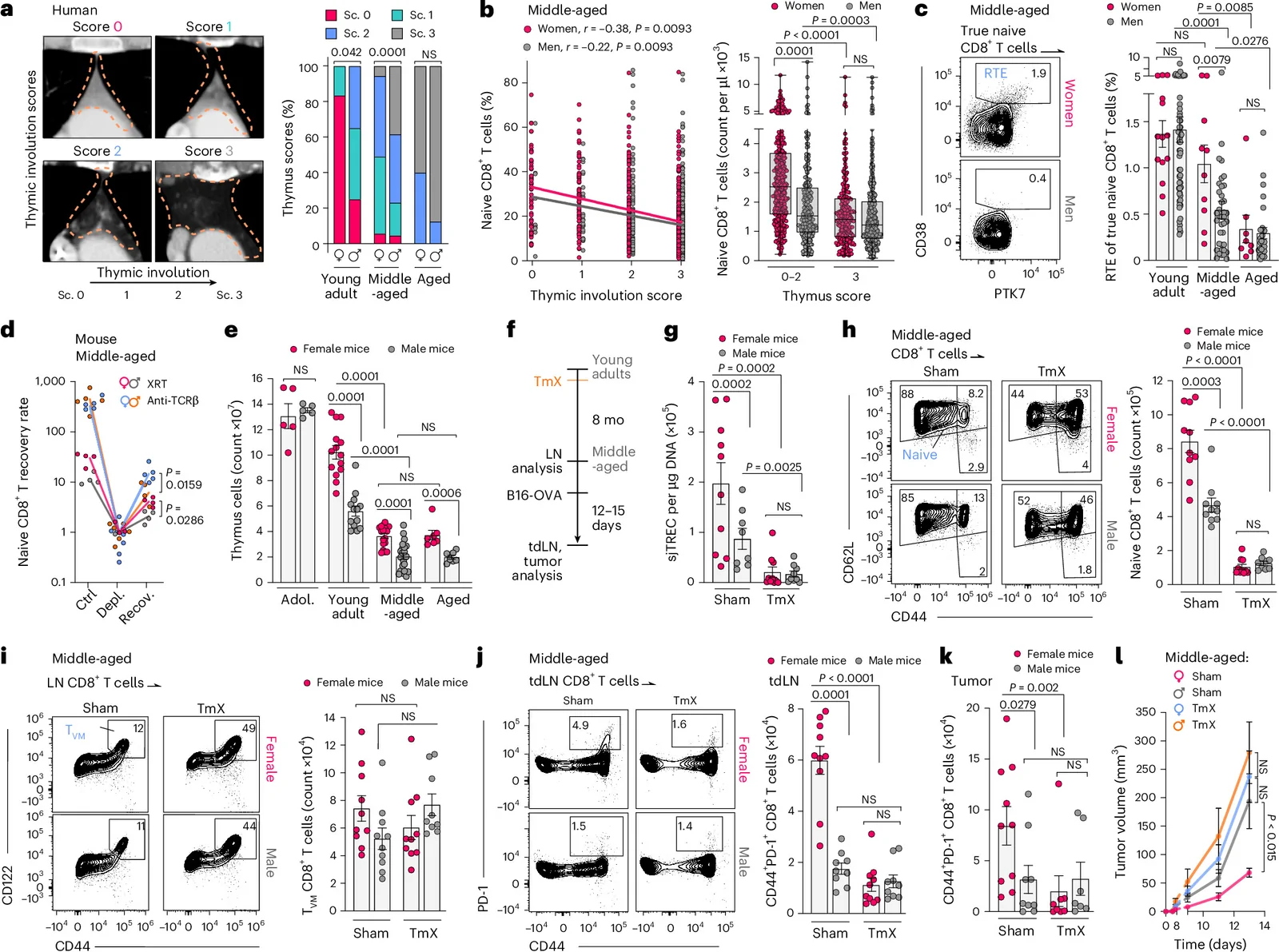
Early thymic involution in male mice and humans limits naive CD8^+^ T cell replenishment and facilitates LN contraction. **a**, Analysis of human thymic involution in computed tomography scans. Score (Sc.) 0: predominantly soft tissue; score 1: parity of fatty and soft tissue; score 2: predominantly fat; score 3: complete fatty replacement. The scoring system details are shown in Extended Data Fig. 7a and previously published data^30,31^. **b**, Left: correlation between the thymic involution score and naive CD8^+^ T cell frequency (women, *n* = 535; men, *n* = 543). Right: number of naive CD8^+^ T cells per microliter of blood in individuals with preserved thymus (score 0–2; women, *n* = 360; men, *n* = 224) or fully involuted thymus (score 3; women, *n* = 224; men, *n* = 224). **c**, Flow cytometry data from human PBMCs for RTEs (CD38^high^) in true naive CD8^+^ T cells (female/male, young adult, *n* = 14/60; middle-aged, *n* = 10/38; aged, *n* = 8/28) from a public dataset^36^. The gating strategy is shown in Supplementary Fig. 5. **d**, Recovery rate of naive CD8^+^ T cells in middle-aged mice 7 days after irradiation (XRT; female, *n* = 4; male, *n* = 4) or anti-TCRβ T cell depletion (depl.; female, *n* = 5, male, *n* = 5) and after a period of recovery (recov.): 28 days for XRT and 19 days for anti-TCRβ. Cell numbers were normalized to the mean at the depletion timepoint. **e**, Total thymocyte counts in female and male mice across the lifespan (female/male, adolescent, *n* = 5/5; young adults, *n* = 14/13; middle-aged, *n* = 18/21; aged, *n* = 7/7). **f**, Experimental setup: TmX was performed in 6-week-old mice, and the mice were aged for 8 months until middle aged, after which an LN was analyzed, and B16-OVA tumors were implanted. The resulting cohort was used for all analyses shown in **g**–**l**. **g**, sjTREC quantification in lymphocytes of LNs from middle-aged mice after sham surgery (female, *n* = 10; male, *n* = 8) or TmX (female, *n* = 10; male, *n* = 9) performed when young. **h**,**i**, Flow cytometry of CD8^+^ T cells and quantification of true naive CD8^+^ T cells (**h**) and T_VM_ cells in middle-aged mice after sham surgery (female, *n* = 10; male, *n* = 10) or TmX (female, *n* = 10; male, *n* = 9; **i**). The gating strategy is shown in Supplementary Fig. 4. **j**,**k**, Flow cytometry analysis of CD8^+^ T cells from the tdLN (**j**; female/male, sham, *n* = 10/9; TmX, *n* = 10/9) or primary tumor (**k**; female/male, sham, *n* = 10/9; TmX, *n* = 8/7). **l**, B16-OVA tumor growth in middle-aged mice after sham surgery or TmX performed when mice were 6 weeks old (female/male, sham, *n* = 10/9; TmX, *n* = 10/9). Data are shown as mean ± s.e.m., with each data point representing a single LN or tumor from an individual animal. Statistics were calculated using the two-sided MWU (**b** (right), **c**–**e** and **g**–**k**), two-sided Fisher’s exact test (**a**), two-sided Spearman correlation (**b**, left) and two-way repeated measures ANOVA (**l**). Source data

**Extended Data Fig. 7:**
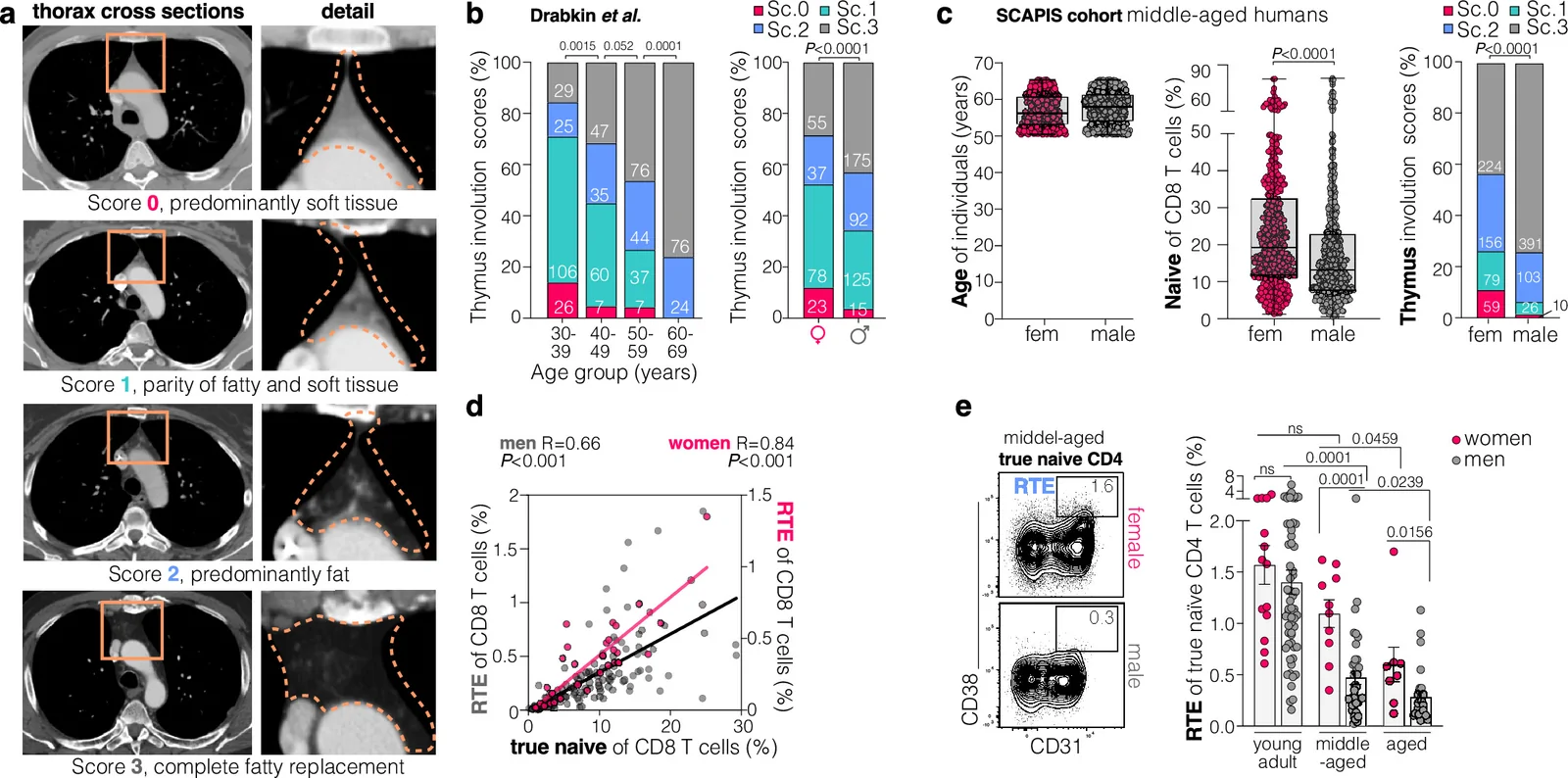
Human thymus and circulating recent thymic emigrants. **a**, Representative images of CT scans and scores to evaluate the involution of human thymuses, score 0: predominantly soft tissue; score 1: parity of fatty and soft tissue; score 2: predominantly fat; score 3: complete fatty replacement^9,10^. **b**, Data published by Drabkin et al.^13^ confirming age-related thymus involution using the scoring system as described in **a**. Numbers in columns indicate number of participants per group. **c**, Age in years (all middle-aged, 50-65 years old), proportion of naive CD8+T cells within the CD8+T cell compartment in blood samples and thymus involution scores of the SCAPIS cohort of middle-aged individuals (women n = 530 and men n = 518). Box plots show median, 25^th^-75^th^ percentiles. **d**, correlation of the frequency of the true naive subset and of RTE relative to CD8+T cells in public data set^12^. **e**, Representative flow cytometry plot and quantification of CD4 RTE and true naive CD4 T cells in human blood (women/men young adults n = 14/60, middle-aged n = 10/38, aged n = 8/27), public data set^12^. Gating strategy in Supplementary Data Fig. 5. Data shown as mean ± SEM with data points representing individual human subjects. Statistics calculated with Fisher’s exact test (b,c), Pearson correlation (d), two-sided MWU test (c,e), ns=nonsignificant. Source data

Given the significant loss of naive CD8^+^ T cells via T_CM_ and T_VM_ cell differentiation, we next asked why this decline is not adequately replenished over time. Although no direct causal link has been established between thymic involution and T_VM_ cell differentiation, the increased abundance of T_VM_ cells under lymphopenic conditions and during aging likely reflects how progressive naive T cell loss makes thymic insufficiency functionally relevant^50,51^. Consistent with published data, we observed thymic involution in humans with significant sex differences, particularly in young adults and middle-aged individuals (Fig. 5a), matching the decline in LN size and naive CD8^+^ T cell numbers measured in both mice and humans (Fig. 2a,h). Using data from a subsample of the Swedish CArdioPulmonary bioImage Study (SCAPIS) cohort^52^, which included thymic health and naive CD8^+^ T cell counts in middle-aged individuals, we showed a significant correlation between poor thymic health and lower numbers of naive CD8^+^ T cells (Fig. 5b). Using data from Drabkin et al.^53^ and the SCAPIS cohort^54^, we also showed age- and sex-related thymic involution using similar computed tomography-based analysis methods (Extended Data Fig. 7a–c). Across individuals, the thymic involution score and recent thymic emigrant (RTE) prevalence strongly correlated with the frequency of naive CD8^+^ T cells, linking thymic atrophy to peripheral naive T cell supply (Fig. 5b,c and Extended Data Fig. 7d). The lack of recovery from the sex-biased loss in naive CD8^+^ T cells results from poorer thymic health in male participants than in age-matched female participants. Interestingly, although CD4^+^ T cell RTEs also declined with age and showed a similar sex bias, naive CD4^+^ T cell numbers remained relatively stable (Extended Data Fig. 7e), consistent with their lower conversion into memory-like cells and increased longevity^37^. In humans, naive CD4^+^ T cell numbers remain stable through midlife, likely due to a compensatory reduction in CD4^+^ T cell loss rate as thymic output wanes^37^, whereas naive CD8^+^ T cells exhibit a more pronounced numerical decline due to T_VM_ cell conversion, with thymic compensation insufficient to maintain their population^35^.

**Extended Data Fig. 8:**
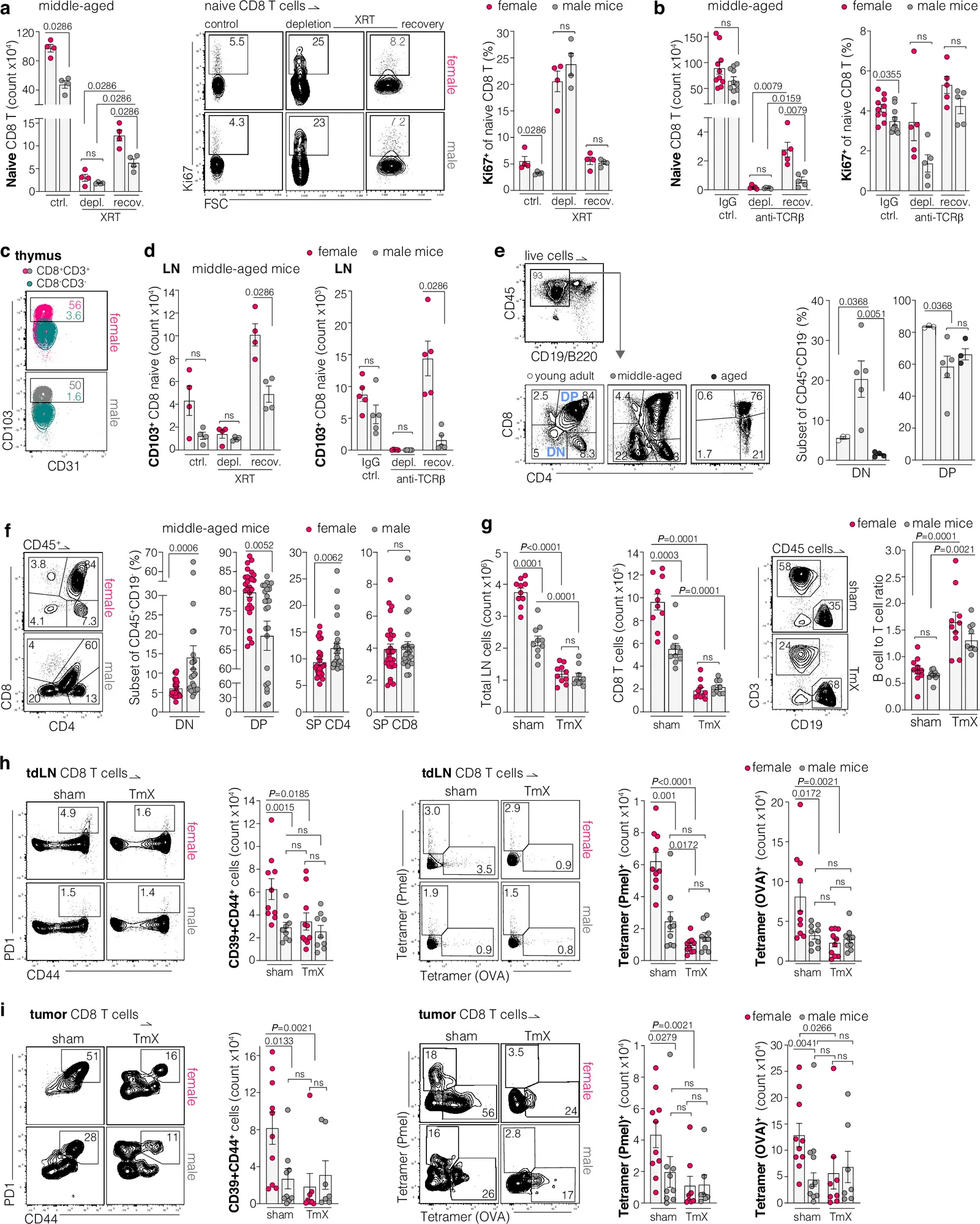
T cell recovery after experimental lymphodepletion, alterations of thymocytes during aging and T cells after thymectomy. **a**, Flow cytometry analysis of naive CD8+T cells and proliferation of naive T cells (Ki67^+^) in middle-aged untreated control mice or after irradiation (XRT, sublethal dose with 4 Gy) induced lympho-depletion (depl., day 7) and after a period of recovery (recov., day 28), n = 4 group. **b**, Naive CD8+T cell counts and Ki67 expression in middle-aged mice treated with IgG control (n = 10 group) or anti–TCRβ antibody (n = 5/group). Shown are depletion at day 7 and recovery at day 19. **c**, Representative flow cytometry plots of CD103 expression in CD8^+^CD3^+^ single-positive (SP) thymocytes, marking recent thymic emigrant (RTE) precursors. CD8⁻CD3⁻ cells serve as gating controls. **d**, Frequencies and absolute numbers of CD103^+^ naive CD8+T cells in inguinal LNs of middle-aged mice at depletion (day 7) and recovery phases after XRT (n = 4/group) or anti–TCRβ (n = 5/group) treatment. **e**, Flow cytometry analysis of thymocytes (CD45^+^CD19^−^/B220^−^) in male mice of different age groups (young adult n = 3, middle-aged n = 5, aged n = 4); including double negative (DN) and double positive (DP) cells. **f**, Flow cytometry analysis of thymocytes (CD45^+^CD19^−^) in middle-aged mice (female n = 27, male n = 26); including DN and DP cells and single positive (SP) cells. **g**, Quantification of total LN cells, T cells, CD8+T cells and the T cell to B cell ratio in ingLNs of middle-aged sham (n = 10/sex) and TmX (female n = 10, male=9) treated mice. **h**, Flow cytometry analysis of CD8+T cells in tdLNs of sham (female n = 10, male=9) and TmX (female n = 10, male=9) mice with B16-OVA tumors. Corresponding to Fig. 5i,j,k. **i**, Flow cytometry analysis of CD8+T cells from the primary B16-OVA tumor of sham (female n = 10, male=9) and TmX (female n = 8, male=7) mice. Corresponding to Fig. 5i,j,k. Data shown as mean ± SEM with data points representing individual mice. Data shown as mean ± SEM with data points representing single LNs or thymuses of individual animals. Statistics calculated with two-sided MWU test (a,b,d,e,f,g,h,i), ns=nonsignificant. Source data

Mimicking the aging effect on LNs with experimental lymphodepletion, naive CD8^+^ T cells recovered faster in middle-aged female mice than in male mice (Fig. 5d). The lymphopenia induced a temporary increase in homeostatic proliferation of naive CD8^+^ T cells in LNs that was similar between sexes (Extended Data Fig. 8a,b). However, several naive CD8^+^ T cells during recovery comprised CD103^high^ RTEs^55^, with a higher prevalence in female mice (Extended Data Fig. 8c,d). Like in humans, we found significant sex differences in thymic cellularity of mice (Fig. 5e and Extended Data Fig. 8e,f). Performing thymectomies (TmX) on young adult mice after the initial establishment of the T cell pool (Fig. 5f) significantly reduced RTEs in LNs (detected by signal joint T cell receptor excision circles (sjTREC)), eliminated the sex differences found in middle-aged sham surgery-treated animals and abolished the female advantage in LN cellularity and naive CD8^+^ T cell numbers (Fig. 5g,h and Extended Data Fig. 8g). These data mirror the pattern observed in humans, where sex differences are evident when thymic tissue is preserved (a score from 0 to 2) but lost following complete thymic involution (score 3; Fig. 5b, right). Interestingly, disrupting the thymus-derived supply of T cells by TmX in 6-week-old mice after the initial establishment of the T cell pool had no impact on the total T_VM_ cell counts (Fig. 5i). These findings are in line with a model in which T_VM_ cells, largely established from early-life thymic emigrants^56^, persist independently of thymic output. Their stable numbers after TmX in young adults reflect a tightly regulated balance of naive-to-T_VM_ cell conversion, shaped by homeostatic constraints such as IL-15 availability and the transient nature of T_VM_ cell residency in LNs, as these cells either die or exit the LN, ultimately contributing to LN contraction during aging.

As a result, the loss of naive CD8^+^ T cell replenishment following TmX neutralized sex differences in tumor-specific recognition and CD8^+^ T cell-mediated tumor suppression in middle-aged mice (Fig. 5j–l and Extended Data Fig. 8h,i), essentially degrading female anticancer immune function in middle age. Collectively, these results demonstrate that the supply of LNs with de novo-generated naive CD8^+^ T cells from the thymus remains relevant even later in life in mice and suggest that thymic output continues to contribute to immune maintenance in middle-aged humans, albeit to a progressively lesser extent in men (Extended Data Fig. 1f).

### Therapeutic thymus regeneration replenishes naive T cells and invigorates tumor recognition

Thymic atrophy with age results not only from diminished bone marrow-derived lymphoid progenitors and stromal cell senescence but also from the suppressive effects of sex hormones, particularly androgens^18^. Although direct evidence is lacking for the extent to which thymopoiesis in humans can be restored through androgen blockade, clinical data suggest that androgens negatively influence naive CD8^+^ T cell levels^57–59^. Sex steroid ablation (SSA) has shown potential to increase thymopoiesis and TIL presence in melanoma and advanced prostate cancer^3,60,61^. In mice, SSA partially reverses thymic involution and regenerates the T cell compartment in younger animals, but has minimal effect at advanced age^62,63^.

**Fig. 6:**
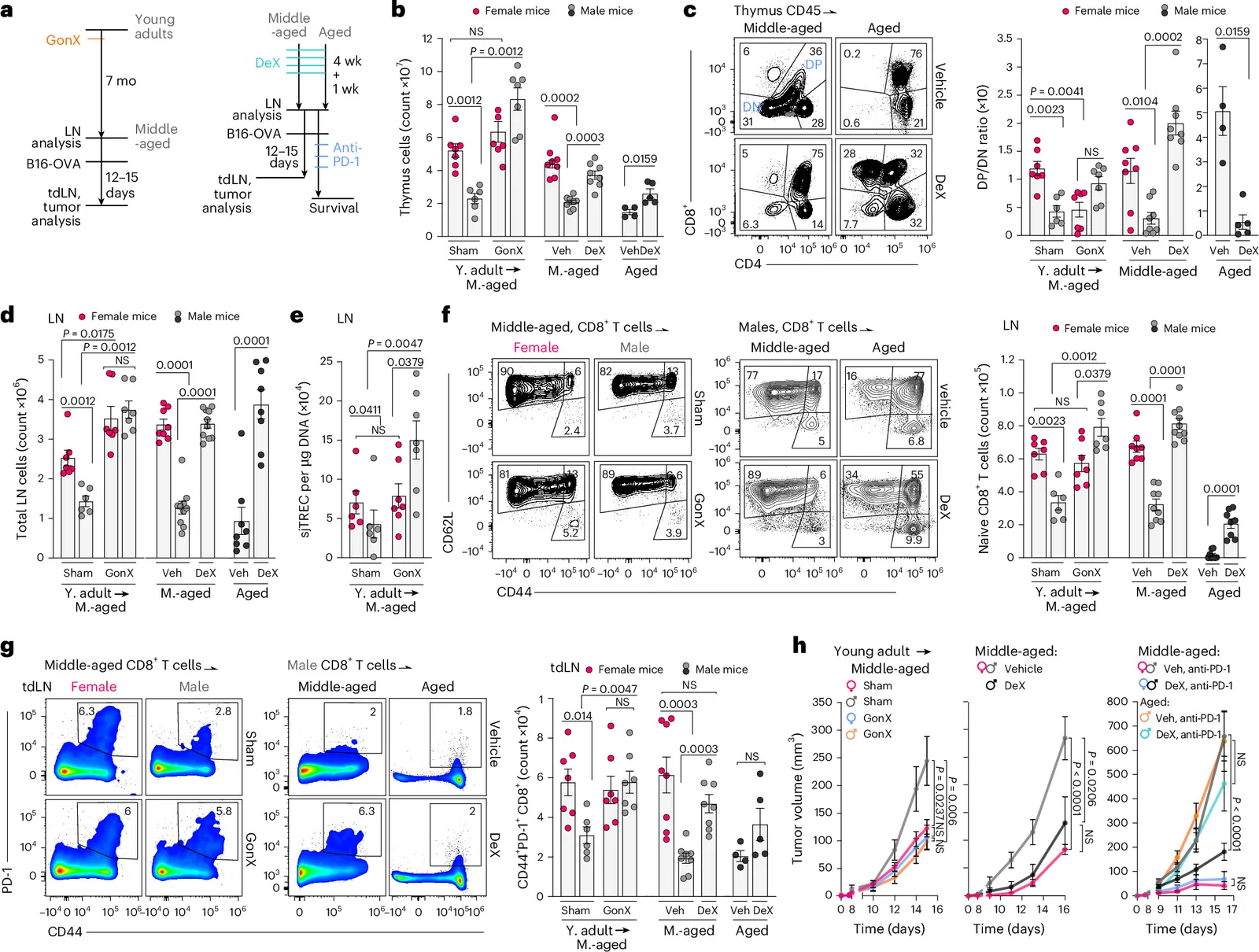
Therapeutic thymus regeneration replenishes naive T cells and reinvigorates tumor recognition. **a**, Experimental setup. Left: young adult and middle-aged mice underwent GonX (ovariectomy for female mice or orchiectomy for male mice). Right: middle-aged and aged mice were treated with vehicle (veh) or DeX before analysis of tumor-naive LNs or implantation of B16-OVA tumors treated with or without anti-PD1. **b**, Total count of thymocytes in mice after sham surgery (female, *n* = 7; male, *n* = 6) or GonX (female, *n* = 7; male, *n* = 7) and vehicle (female, *n* = 8; male *n* = 8) or DeX (male, *n* = 8) treatment in middle-aged and aged male mice (vehicle, *n* = 4; DeX, *n* = 5). **c**, Flow cytometry analysis of thymocytes and double-positive (DP) to double-negative (DN) ratio. **d**, IngLN cellularity after treatment as described in **a**. **e**, sjTREC quantification in lymphocytes of LNs after sham surgery (female, *n* = 6; male, *n* = 6) or GonX (female, *n* = 7; male, *n* = 7). **f**, Flow cytometry analysis of naive CD8^+^ T cells from ingLNs of mice. The gating strategy is shown in Supplementary Fig. 4. **g**, Flow cytometry analysis of CD8^+^ T cells from tdLNs. The gating strategy is shown in Supplementary Fig. 2. **h**, B16-OVA tumor growth in sham (female, *n* = 7; male, *n* = 6) and GonX (female, *n* = 7; male, *n* = 7) middle-aged mice (left), vehicle (*n* = 8/sex)- or DeX (male, *n* = 8)-treated middle-aged mice (middle) and vehicle (female, *n* = 11; male, *n* = 13)- or DeX (female, *n* = 11; male, *n* = 11)- and anti-PD-1-treated middle-aged mice and aged male mice (right; vehicle, *n* = 8; Dex, *n* = 7). Data are from one independent analysis and are shown as mean ± s.e.m., with each data point representing a single LN or tumor from an individual animal. Statistics were calculated using two-sided MWU (**b**–**g**) and two-way repeated measures ANOVA (**h**) to assess overall effects across time and group. Source data

**Extended Data Fig. 9:**
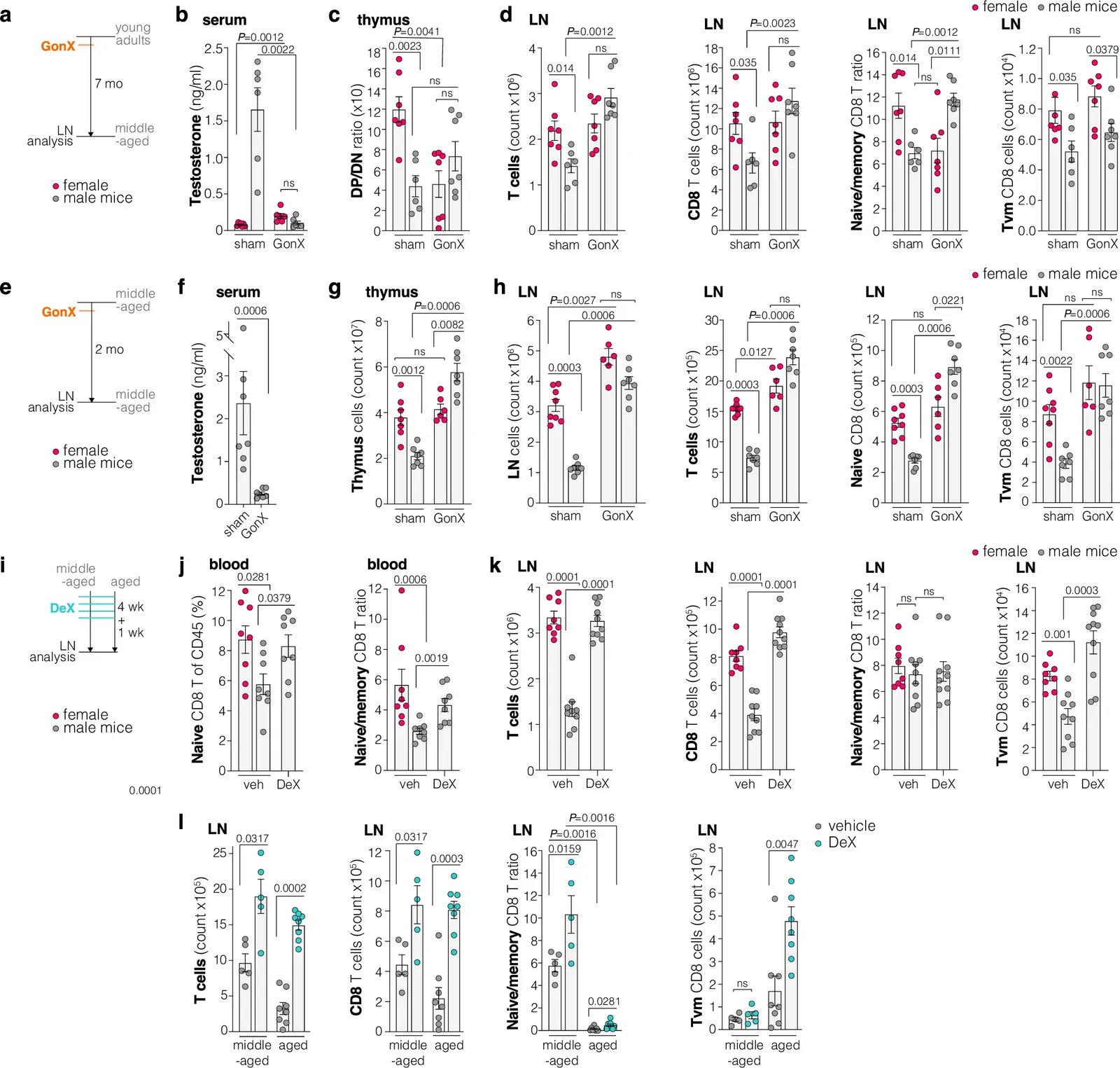
Consequences of sex steroid ablation for the thymus and lymph nodes. **a**, Experimental setup, Gonadectomy (GonX) in adult mice and analysis middle-aged. **b**, Analysis of serum testosterone levels (female/male sham n = 7/6, GonX n = 6/6). **c**, Flow cytometry analysis of thymocytes (CD45^+^CD19^−^/B220^−^); ratio of double positive (DP) and double negative (DN) cells (female/male sham n = 7/6, GonX n = 7/7). **d**, Flow cytometry analysis and quantification of T cells from tumor-naive LNs (female/male sham n = 7/6, GonX n = 6/6). **e**, Experimental setup, GonX in middle-aged mice and analysis two months after surgery. **f**, Analysis of serum testosterone levels (sham n = 7, GonX n = 7). **g**, Number of total thymocytes (female/male sham n = 7/7, GonX n = 6/7). **h**, LN cellularity and flow cytometry analysis of T cells from tumor-naive LNs (female/male sham n = 7/6, GonX n = 5/6). **i**, Experimental setup, middle-aged and aged mice were treated with vehicle (veh) or Degarelix (DeX). **j**, Flow cytometry analysis of naive and memory CD8+T cells in blood of middle-aged mice (n = 8/group). **k**, Flow cytometry analysis of T cells in tumor-naive LNs from middle-aged mice (female/male vehicle n = 8/9, male DeX n = 10). **l**, Flow cytometry analysis of T cells from tumor-naive LNs in male mice (middle-aged vehicle/DeX n = 5/5, aged vehicle/DeX n = 8/8). Data shown as mean ± SEM with data points representing single LNs, blood samples or thymuses of individual animals. Statistics calculated with two-sided MWU test (b,c,d,f,g,h,j,k,l), ns=nonsignificant. Source data

To test whether therapeutic SSA could restore naive CD8^+^ T cell availability, improve LN function and drive better tumor recognition, we applied both long-term and short-term strategies in mice: (1) surgical gonadectomy (GonX) in early adulthood or middle age and (2) pharmacologic SSA using the Food and Drug Administration-approved gonadotropin-releasing hormone antagonist degarelix (DeX) in middle-aged and aged male mice (Fig. 6a). Of note, long-term GonX in female mice (estrogen depletion via ovariectomy) did not significantly alter thymic cellularity, RTE frequency in LNs (sjTREC content) or naive CD8^+^ T cell numbers (Fig. 6b,e,f). By contrast, both surgical and chemical inhibition approaches for androgens led to increased thymus cellularity and normalized thymocyte development, as reflected by restored double-positive to double-negative ratios in male mice (Fig. 6b,c and Extended Data Fig. 9a–c). Importantly, SSA resulted in robust repopulation of LNs, with elevated numbers of RTEs (Fig. 6d,e). This translated into a significant restoration of naive CD8^+^ T cell numbers in middle-aged male mice (Fig. 6f and Extended Data Fig. 9d–j). In aged male mice, DeX treatment also increased naive CD8^+^ T cells, but to a lesser extent, and absolute numbers remained reduced relative to middle-aged mice (Fig. 6f and Extended Data Fig. 9i,l).

**Extended Data Fig. 10:**
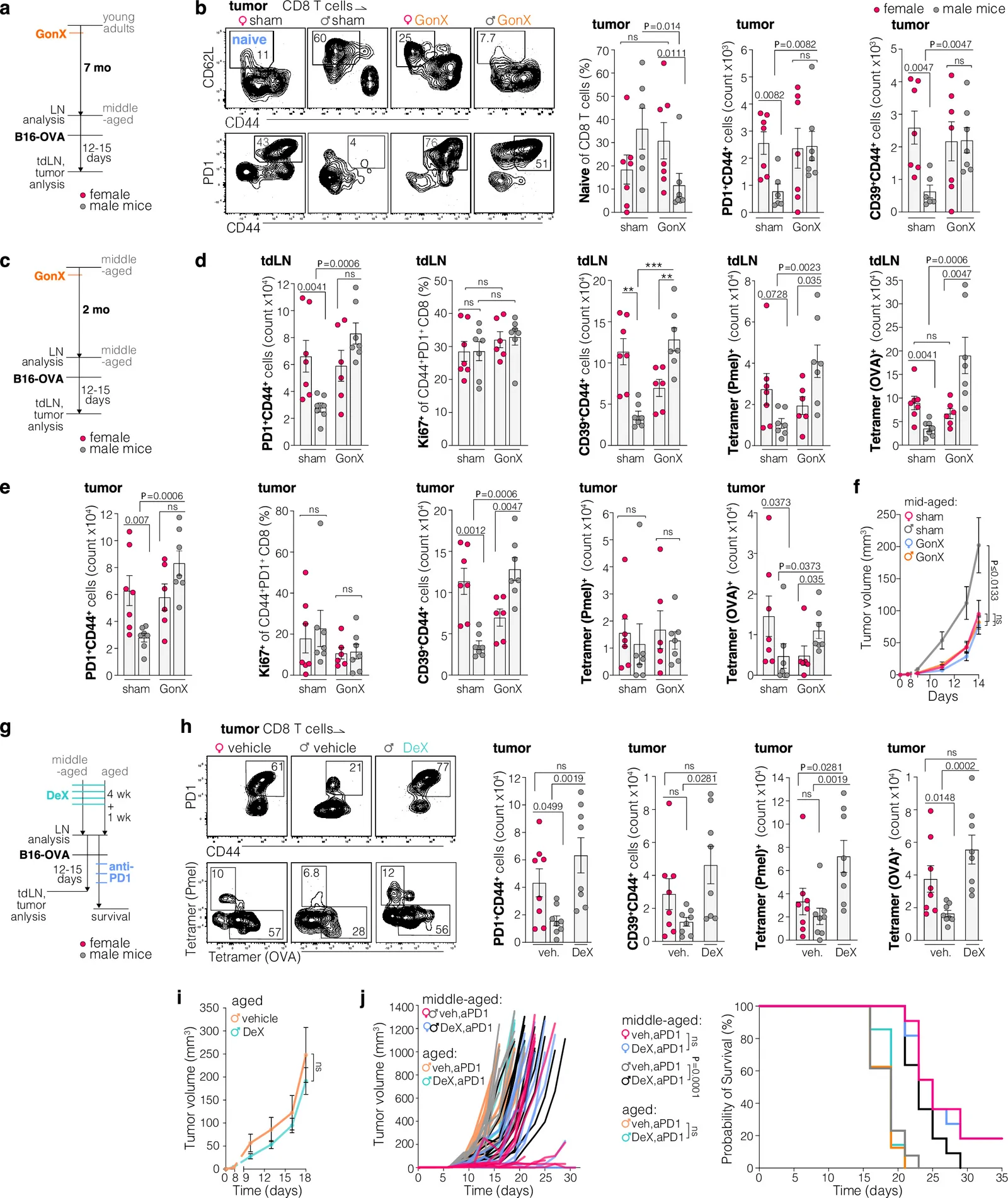
Consequences of sex steroid ablation tumor recognition in tdLNs and the primary tumor. **a**, Experimental setup, gonadectomy (GonX) in young adults followed by B16-OVA tumor implantation and analysis in middle-aged mice. **b**, Flow cytometry analysis of T cells in tumors (female/male sham n = 7/6, GonX n = 7/7). **c**, Experimental setup, GonX in middle-aged mice and analysis or B16-OVA tumor implantation two months after surgery. **d**, Flow cytometry analysis of tdLNs (female/male sham n = 7/7, GonX n = 6/7) and **e**, TILs (female/male sham n = 7/7, GonX n = 6/7). **f**, B16-OVA tumor growth in mice after sham or GonX surgery, n = 7/group. **g**, Experimental setup, left, young adult and middle-aged mice were gonadectomized (GonX, ovariectomy for female mice, orchiectomy in male mice) or, right, middle-aged and aged mice were treated with vehicle (veh) or Degarelix (DeX) before analysis of tumor-naive LNs, or implantation of B16-OVA tumors with anti-PD1 antibody treatment. **h**, Flow cytometry analysis of TILs (female/male vehicle n = 8/8, male DeX n = 7). **i**, B16-OVA tumor growth in vehicle or DeX treated aged mice (vehicle n = 4, DeX n = 5). **j**, B16-OVA tumor growth and survival probability in middle-aged and aged mice after treatment with DeX and anti-PD1 (female/male middle-aged vehicle n = 11/13, DeX n = 11/11; aged vehicle n = 8, DeX n = 7). Data shown as mean ± SEM with data points representing single LNs or tumor of individual animals. Statistics calculated with two-sided MWU test (b,g,e,h), two-way repeated measures ANOVA (f,i), or Log-rank test (j) ns=nonsignificant. Source data

Functionally, these changes eliminated sex-based differences in tumor recognition and response. Following SSA, middle-aged male mice exhibited increased frequencies of antigen-experienced CD8^+^ T cells in the tdLNs, including CD44^+^PD-1^+^ and CD39^+^ subsets, which mark repeatedly stimulated tumor-reactive T cells. Further, tumor-antigen-specific (tetramer^+^) CD8^+^ TILs were also increased, mirroring responses seen in female mice. Correspondingly, tumor growth dynamics equalized between sexes (Fig. 6g,h and Extended Data Fig. 10a–i), with SSA-treated male mice controlling tumor growth better than control-treated male mice. Preconditioning of middle-aged male mice with DeX also restored numbers of naive CD8^+^ T cells in LNs and increased the therapeutic response to subsequent anti-PD-1 immunotherapy (Fig. 6h, right, and Extended Data Fig. 10j).

Together, these findings demonstrate that SSA can reinvigorate thymic output, replenish the naive CD8^+^ T cell pool and enhance antitumor immunity in middle-aged male mice, thereby eliminating functional sex differences. These data also show that improving the naive CD8^+^ T cell pools in middle-aged male mice can increase responses to anti-PD-1 immunotherapy. However, the diminished efficacy of this intervention in aged male mice highlights the narrowing therapeutic window for immune regeneration later in life.

## Discussion

Our study highlights sex- and age-related disparities in naive CD8^+^ T cell homeostasis and its connection to functional antigen recognition in LNs, illuminating a previously underappreciated link between dynamics of naive T cell abundance, thymic involution and impaired cancer immunity during aging. Age-related thymic involution and its consequences for peripheral T cell homeostasis have been studied for decades, with foundational work establishing that progressive loss of thymic output leads to a numerical and functional decline in naive T cells and contraction of TCR diversity with age^14,15^. More recent clinical evidence has linked thymic atrophy directly to increased cancer risk, loss of immune checkpoint blockade responsiveness and mortality in humans^14,15,64^. We demonstrated that male mice and men experience an earlier and more profound decline in naive CD8^+^ T cell abundance, which resulted in fewer activated tumor-reactive effectors and compromised anticancer immunity in middle age. Although T cell infiltration in tumors generally correlates positively with prognosis, the functional status and antigen specificity of TILs are critical determinants of clinical outcomes. Our findings align with reports demonstrating that increased frequencies of naive-like TILs, often associated with age^24^, correlate with ineffective anticancer responses and poor therapeutic outcomes, highlighting the need for functional rather than numerical assessments of TIL populations in clinical contexts^22,25,26^. Although aged mice exhibit a significant reduction in TCR diversity of up to 80%, which leads to measurable deficits in immune functionality, it remains less clear whether a reduction in T cell clones has equivalent consequences in humans^65,66^. Notably, although LNs in aged humans maintain relative clonal diversity^66^, our data suggest that effective immune surveillance depends on the absolute number of unique T cell clones available in draining LNs. Recent studies^10,12^ have shown that tdLNs continuously generate stem-like CD8^+^ T cell populations that serve as a renewable source of effector cells and are enriched for high-affinity clones. In this context, the size and diversity of the naive T cell pool represent a key upstream determinant of the landscape from which these stem-like CD8^+^ T cell populations are derived. The reduced naive T cell availability reduces the stochastic probability of antigen recognition and limits the generation of high-affinity, stem-like T cell clones capable of sustaining effective responses. In this framework, the tumor serves primarily as a source of locoregional antigen, while the LN naive T cell pool determines the likelihood that a cognate clone will be activated^12^. Tumor immunogenicity acts as a modulating variable in this relationship, as a higher density of neoantigens and antigens with specific recognition motifs^67^ can partially compensate for a reduced naive precursor pool, as demonstrated in the context of poorly versus highly immunogenic tumors^68^.

A central insight of our study is the dual mechanism driving naive CD8^+^ T cell loss in men: accelerated, antigen-agnostic conversion into T_VM_ cells combined with an earlier decline in thymic output (Extended Data Fig. 1f). Prior work has shown that total peripheral T cell numbers, largely maintained by the CD4^+^ T cell compartment, remain relatively stable with age through homeostatic proliferation and increased cell lifespan^37^, whereas CD8^+^ T cells progressively decline^33,34,38,39^. In line with a recent study^14^, our data in mice and humans refine this paradigm by demonstrating that naive CD8^+^ T cells are selectively depleted, in part through differentiation into T_VM_ cells. The selective naive CD8^+^ T cell depletion is insufficiently compensated in males because of earlier thymic involution, which is consistent with the human data (Figs. 2g,h and 5a–c, and Extended Data Figs. 4c and 7a–d). This selective vulnerability of CD8^+^ T cells is likely explained by the propensity of more self-reactive CD5^high^ CD8^+^ T cells to undergo differentiation into T_VM_ cells. Consistent with this concept, CD4^+^ T cells exhibit a substantially lower propensity for antigen-agnostic memory differentiation than CD8^+^ T cells^40,69,70^. Together, these findings suggest that maintaining an abundant and clonally diverse pool of naive CD8^+^ T cells becomes increasingly dependent on de novo thymic output with age.

Our results support the critical role of thymic output in maintaining naive CD8^+^ T cell populations throughout adulthood. The observed sex disparity in thymic involution during the transition from young adulthood to middle age suggests a hormonal contribution, with androgens exerting potent inhibitory effects on thymopoiesis^18^. In our study, therapeutic thymus regeneration via androgen blockade restored naive CD8^+^ T cell numbers, enhanced cancer antigen recognition and reversed sex-related differences in cancer immunity in middle-aged male mice (Fig. 6). These data highlight the potential of therapeutic interventions targeting thymic regeneration, especially within a therapeutic window before advanced age-related changes such as thymus epithelial cell senescence, LN stromal fibrosis and structural degeneration irreversibly limit regeneration capacity^62,71,72^.

Beyond strategies aimed at enhancing thymic output, limiting premature T_VM_ cell differentiation may represent a complementary way to preserve naive CD8^+^ T cell numbers. Our finding that transient artificial induction of T_VM_ cells in female mice recapitulates male-like aging phenotypes suggests that early-life skewing toward T_VM_ cells can have durable impacts on immune homeostasis. Mechanistically, T_VM_ cell formation depends on IL-15 availability and clone-specific self-reactivity^40,41^, but how these factors are regulated in physiological settings remains incompletely understood. These findings motivate future studies to define the upstream regulators of T_VM_ cell differentiation and evaluate whether targeting these pathways can preserve naive CD8^+^ T cell pools during aging.

The human immune and thymus data were reproduced across independent cohorts, supporting the translational relevance of our findings. Although the computed tomography-based LN volume analyses are exploratory and subject to clinical and anatomical confounders, the observed age- and sex-associated trends are consistent with the immune phenotypes identified in both mice and humans. Together, these findings support further exploration of thymus-targeted interventions, such as short-term SSA treatment, in clinical settings where insufficient de novo T cell priming limits antitumor immunity.

Collectively, our study identifies accelerated thymic involution and antigen-agnostic differentiation of naive CD8^+^ T cells into T_VM_ cells as complementary mechanisms driving sex-biased immune aging. These findings identify preservation of thymic function and the naive CD8^+^ T cell pool as promising strategies to maintain LN immune competence and improve antitumor immunity during aging.

## Methods

### Compliance statement

All experiments were performed at the Massachusetts General Hospital (MGH) and approved by the Institutional Animal Care and Use Committee of the MGH under protocols 2023N000101, 2011N000085 and 2004N000123.

### Mice

WT C57BL/6 mice, *Lta*^−/−^ mice (B6.129s2-*Lta*^*tm1Dch*^/J from The Jackson Laboratory, 002258), CD45.1 congenic mice (B6.SJL-*Ptprc*^*a*^*Pepc*^*b*^/BoyJ from The Jackson Laboratory, 002014) and OT-1 (C57BL/6-Tg(*TcraTcrb*)1100Mjb/J) were used as animal models. All mice were bred and maintained in animal facilities of the MGH, with housing in accordance with procedures outlined in the National Institutes of Health’s (NIH)’s Guide for the Care and Use of Laboratory Animals. Mice received a standard pelleted rodent diet and tap water ad libitum and were maintained at 20–24 °C, with 70% humidity on a 12-h light/12-h dark cycle. Age- and sex-matched groups of adolescent (4–5 weeks), young adult (7–10 weeks), middle-aged (9–12 months) and aged (>18 months) female and male mice were used in each experiment unless specified otherwise.

### Animal models and tumor cell lines

The B16F10 melanoma cell line (ATCC) was cultured in DMEM supplemented with 10% heat-inactivated FBS (Gibco), 1% penicillin–streptomycin (Gibco) and 1% ʟ-glutamine (Gibco) at 37°C and 5% CO_2_. B16-OVA melanoma cells (ovalbumin-expressing B16F10) were kindly provided by S. Thomas (Georgia Institute of Technology). B16-SIINFEKL-zsGreen cells were generated in-house, as previously described^73^. To exclude sex-mismatched antigen effects, B16F10 cells were verified to lack Y-chromosome-encoded sequences, ensuring that tumor recognition was not influenced by sex-specific histocompatibility antigens (see ‘PCR to determine sex from genomic DNA’ and Extended Data Fig. 2a). All cell lines were used at a low passage number (below passage 15) and regularly tested for mycoplasma contamination using the MycoAlert Mycoplasma Detection Kit (Lonza). Only mycoplasma-negative cultures were used for in vivo experiments. For tumor engraftment, B16F10 or B16-OVA cells (5 × 10^5^) were injected intradermally into the lower flank. Animals were monitored daily, and tumor volumes were measured every 2–3 days. Tumor volume was estimated as tumor volume = width × length × (width / 2). For survival analyses, animals were killed based on approved humane endpoints once the tumor exceeded 1,000 mm^3^ or reached 2 cm in any diameter. The maximal tumor burden permitted by the approved animal protocol was not exceeded in any experiment.

### Study population

The SCAPIS is a general population-based prospective study^52^. The use of data from the SCAPIS was approved by the appropriate regional ethics review board, and all participants provided written informed consent. Analyses in the present study were performed using deidentified data from the SCAPIS cohort. Middle-aged participants (50 to 64-year-old women and men) underwent comprehensive clinical examination, including imaging and functional studies of the heart and lungs, which included the thymus, and completed extensive questionnaires to assess lifestyle as well as prior and current medication regimens. The SCAPIS Leukocyte subcohort of 1,078 participants was consecutively recruited from the SCAPIS center at Linköping University Hospital to establish an immune cell profile in whole fresh blood using flow cytometry^54^. Twenty-nine participants (2.7%) were excluded from analysis: 9 (0.8%) due to missing images, 6 (0.6%) with anterior mediastinal lymphadenopathy or tumor, 5 (0.5%) with prior surgery preventing evaluation, 2 (0.2%) with imaging artifacts and 7 (0.6%) for miscellaneous anatomical or technical reasons.

### Volume segmentation in computed tomography scans

Computed tomography scans of humans are from the Cancer Imaging Archive of the NIH^30,31^ and were analyzed using 3D Slicer (version 5.6.2; Supplementary Fig. 1c). These datasets did not include clinical data, and thus the health status of the individuals was unknown. To ensure that LN measurements reflected non-pathologic anatomical variation, we excluded all scans showing radiologic evidence of lymphadenopathy or other abnormalities that could influence LN size.

#### LN analysis

Body scans were cropped into the axillary or inguinal regions. Superficial skin areas were excluded. The segmentation threshold was adjusted to include LNs while excluding surrounding fatty tissue and skin. Using three-dimensional volume rendering, LNs were identified and surrounding tissues were removed manually until only individual LNs remained. The average LN volume per individual was used for statistical analysis. Datasets with one or more pathologically enlarged LN (>700 mm^3^) were excluded.

#### Spleen analysis

Scans were cropped to the region beneath the lungs to isolate the spleen. The segmentation threshold was adjusted to include the spleen while excluding surrounding fatty tissue. Three-dimensional volume rendering was used to identify the spleen and manually remove extraneous tissue.

#### Thorax analysis

Scans were cropped between the clavicles and the upper pelvis. The segmentation threshold was set to include all tissue within the thorax. Using three-dimensional volume rendering, objects not part of the human thorax (for example, the scanning table) were removed.

#### Thymus analysis

Thymus scans were assessed as described previously^54^. In brief, thymus fat and soft tissue content was visually analyzed and graded on a scale from 0 to 3. A score of score 0 indicates predominantly soft tissue, a score of 1 indicates parity between fatty and soft tissues, a score of 2 indicates predominantly fat, and a score of 3 indicates complete fatty replacement (Extended Data Fig. 7a).

#### Thymus analysis of the SCAPIS cohort

Computed tomography scans of the thorax were performed according to the guidelines of the SCAPIS protocols^52^. All scans were reviewed using a fixed window setting and evaluated by an experienced thoracic radiologist^54^.

### Organ resection procedures

All surgical procedures were performed under ketamine/xylazine (intraperitoneal) anesthesia. Procedures were performed under a dissecting microscope where applicable. Animals were monitored daily after surgery for signs of pain, infection, abnormal behavior and changes in body weight.

#### LN dissection

A 0.5-cm skin incision was made to expose the ingLN. Vessels were compressed with fine curved forceps, and the LN was excised. The skin was closed with wound clips, and mice recovered for ≥14 days.

#### Splenectomy

The spleen was accessed via an incision through the skin and peritoneum at the right flank. Vascular connections at the splenic hilum were ligated, and the spleen was removed. The peritoneum and skin were closed, and mice recovered for ≥14 days.

#### TmX

A semicircular skin incision along the upper thorax was followed by a partial sternal incision to expose the anterior mediastinum. Both thymic lobes were identified and removed by blunt dissection, taking care to avoid injury to blood vessels and the pericardium. Any remaining thymic tissue was removed by gentle aspiration. The chest wall was closed with absorbable suture, and the skin was closed with wound clips. Mice were allowed to recover and mature to middle age before experiments. Complete thymic removal and absence of thymic regeneration were verified visually during surgery and confirmed at experimental endpoint.

#### Orchiectomy

Mice were placed in dorsal recumbency, and the scrotal region was shaved and disinfected. The testis, epididymis and associated fat pad were gently exteriorized through a small scrotal incision. A ligature of absorbable suture was placed below the testis and epididymis, which were then excised. The scrotal incision was closed with absorbable suture. Successful orchiectomy was confirmed by measurement of serum testosterone levels by enzyme-linked immunosorbent assay (ELISA) at the experimental endpoint (Extended Data Fig. 9b,f). Mice recovered for ≥14 days before downstream analysis.

#### Ovariectomy

Mice were placed in ventral recumbency, and the dorsal skin overlying the ovaries was shaved and disinfected. Ovaries were accessed through bilateral midline incisions along the back. A ligature of absorbable suture was placed at the oviduct–uterus boundary, and the ovaries were excised. The peritoneum was closed with absorbable suture, and the skin was closed with wound clips. Successful ovariectomy was confirmed by atrophy of the uterine horns at the experimental endpoint. Mice recovered for ≥14 days before downstream analysis.

### Chemical SSA

DeX (MedChemExpress, 214766-78-6) was dissolved in a solution of 10% DMSO, 40% PEG3000, 5% Tween-80 and 45% saline and was administered subcutaneously at 0.25 mg once weekly for four consecutive weeks. Control mice received vehicle only.

### In vivo treatments

Mice were injected intraperitoneally with immune-modulating antibodies or cytokine complexes. For PD-1 blockade, monoclonal anti-PD-1 (clone RMP1-14, Bio X Cell) was administered at 10 mg per kg (body weight) three times every 3 days. For T cell depletion, monoclonal anti-TCRβ (clone H57-597, Bio X Cell) was given at 10 mg per kg (body weight) once. For CD8+ T cell depletion, anti-CD8α mAb (clone 53-6.7, Bio X Cell) was administered at 10 mg per kg (body weight), four times every 5 days. Efficient CD8^+^ T cell depletion was confirmed by flow cytometry analysis of peripheral blood before continuation of downstream experiments (Extended Data Fig. 2c). For IL-15 Cx treatment, IL-15 Cx (6 µg; Sino Biological, CT113-M08H) or vehicle (0.9% NaCl solution) was administered once per week for four weeks.

### Mouse immunization

Tumor cell lysates were prepared by subjecting 1 × 10^8^ cells per ml in sterile water to five freeze–thaw cycles (liquid nitrogen and 37 °C water). Fifty microliters of tumor cell lysate or DQ-OVA solution (Invitrogen, D12053) containing 10 µg of lipopolysaccharide (Sigma-Aldrich, L4774) as an adjuvant, or PR8 virus particles (1 × 10^5^ inactivated particles with 2 µg of polyinosinic:polycytidylic acid and 2 µg of monoclonal anti-CD40 (clone MR-1, Bio X Cell)) or respective controls were injected intradermally into the lower flank. Anti-CD40 was included to mimic CD4^+^ T cell help and promote efficient dendritic cell activation and crosspriming. Draining ingLNs and the contralateral (non-draining) ingLNs were analyzed 24 or 72 h after immunization.

### Tissue dissection and single-cell preparation

Mice were killed, and tissues were resected and mechanically dissociated. Individual LNs were mechanically dissociated by gently pressing them with a syringe plunger against the surface of a sterile Petri dish. Single-cell suspensions from the thymus were filtered through a 40-µm strainer. Single-cell suspensions from tumor and spleen were filtered through a 70-µm strainer. Cells in the resulting suspension were counted before any filtering or centrifugation to ensure reliable cell counts without technical artifacts. For flow cytometry analysis of APCs, LNs were treated with 1 mg ml^−1^ collagenase D (Sigma) and 0.1 mg ml^−1^ DNase I (Roche, in HBSS) for 30 min at 37 °C before mechanical dissociation. Blood and spleen samples underwent hemolysis with ammonium–chloride–potassium buffer (Fisher Scientific, 50-983-219).

### Flow cytometry

Single-cell suspensions were incubated with LIVE/DEAD Zombie dye (BioLegend, 423108) and anti-mouse CD16/CD32 in PBS for 15 min at 4 °C to block Fc receptors and stain for viability. When applicable, tetramers were added for 1 h at room temperature. Surface staining was then performed with fluorochrome-conjugated antibodies in flow buffer (2% fetal calf serum and 0.5 mM EDTA in PBS) for 20 min at room temperature (see Supplementary Table 1 for the list of antibodies). After washing, cells were fixed and permeabilized using the Foxp3/Transcription Factor Staining Set (eBioscience, 00-5523-00) for 30–60 min at 4 °C, followed by intracellular staining for ≥1 h at 4 °C. Data were acquired using an Aurora spectral flow cytometer (Cytek, five-laser configuration) and analyzed with FlowJo (v.10.1, TreeStar). Gating strategies are shown for mouse tumor samples (Supplementary Fig. 2), mouse lymphocytes (Supplementary Fig. 4), mouse thymocytes (Extended Data Fig. 8e) and human PBMCs (Supplementary Fig. 5).

### In vitro stimulation and proliferation assays

Sorted naive CD8^+^ T cells were stained with CFSE per the manufacturers’ instructions (Thermo Fisher, C34554) or left unstained and stimulated on plates coated with anti-CD3 (5 µg ml^−1^; Biolegend, 100340) and anti-CD28 (4 µg ml^−1^; Biolegend, 102116). The cells were incubated for 48 h in complete RPMI1640 medium supplemented with 10% FBS (Gibco), 1% penicillin–streptomycin (Gibco), 1% ʟ-glutamine (Gibco), 1% HEPES (Gibco) and 0.1% β-mercaptoethanol (Sigma) at a concentration of 5 × 10^5^ cells per well in a 96-well, flat-bottom anti-CD3/CD28-coated plate. For the last 4 h of incubation, brefeldin A (Biolegend, 420601) was added. Intracellular staining for IFNγ, IL-2 and TNF was performed as described above.

### mRNA sequencing and analysis

Magnetically sorted CD8^+^ T cells (StemCell, 19853) were used to isolate naive CD8^+^ T cells, defined as CD44⁻CD49d⁻CD122^−^, and T_VM_ cells, defined as CD44^+^CD49d⁻CD122^+^. Cell sorting was performed on a Sony MA900 cell sorter, and a minimum of 200,000 cells per population were collected. A total of 100,000 sorted cells were immediately resuspended in 50 µl of Zymo DNA/RNA Shield and stored at room temperature until further processing. The remaining 100,000 cells per sample were activated on anti-CD3/CD28-coated 96-well plates (Ultra LEAF Purified anti-mouse CD3ε, 5 µg ml^−1^; Ultra LEAF Purified anti-mouse CD28 4 µg ml^−1^) and incubated for 5 h before resuspending in 50 µl of Zymo DNA/RNA Shield. Three biological replicates were collected per group. Total RNA extraction, library preparation, sequencing and data processing were performed by Plasmidsaurus. Briefly, total RNA was extracted from sorted cells preserved in Zymo DNA/RNA Shield using a bead-based extraction approach. RNA concentration was assessed by fluorescence-based quantification before library preparation. Sequencing libraries were generated using a 3′-end counting approach: mRNA was converted to cDNA via reverse transcription using a poly(dT)VN primer, followed by second-strand synthesis, tagmentation, indexing and amplification. Unique molecular identifiers (UMIs) and unique dual indices were incorporated to enable PCR deduplication and prevent index hopping. Single-end sequencing (~90-base pair read length) was performed on an Illumina platform. Raw FASTQ files were generated and demultiplexed using BCL Convert (v4.3.6) and fqtk (v0.3.1). Read quality filtering was performed with FastP (v0.24.0), including poly-X tail trimming, 3′ quality-based trimming, a minimum Phred score of 15 and a minimum read length of 50 bp. Filtered reads were aligned to the mouse reference genome (GRCm39_114) using STAR (v2.7.*) with non-canonical splice junction removal. BAM files were coordinate sorted with samtools (v1.21), and PCR/optical duplicates were removed by UMI-based deduplication using UMICollapse (v1.1.0). Alignment quality metrics were assessed with RustQC (v0.2.1) and summarized using MultiQC (v1.33). Gene-level counts were quantified with featureCounts (Subread v2.1.1) using strand-specific counting, with exons and 3′ untranslated regions as feature identifiers grouped by gene_id. Sample-level quality and reproducibility were assessed by principal component analysis and pairwise Pearson correlation of trimmed mean of *M*-value-normalized counts. Differential gene expression analysis was performed using Edge Python (v0.2.5). Genes with a false discovery rate of <0.05 and an absolute log_2_ fold change of >1 were considered differentially expressed. Genes used for senescence gene sets included those associated with cell cycle arrest (*Cdkn1a, Cdkn2a, Cdkn1b, Cdkn2b, Rb1, Trp53*), inflammatory secretion/senescence-associated secretory phenotype (*Il6, Il1a, Il1b, Cxcl1, Ccl2, Ccl5, Tnf, Igfbp3, Igfbp7, Mmp3, Mmp9*), DNA damage response (*H2ax, Atr, Atm, Chek1, Chek2, Brca1, Trp53bp1*), resistance to apoptosis (*Bcl2, Bcl2l1, Mcl1, Birc5, Cflar*), oxidative stress (*Nox4, Hmox1, Sod1, Sod2, Cat, Gpx1*) and terminal differentiation (*Klrc1, Klrc2, Klrk1, Klrb1c, Klrd1*). The bulk RNA-seq dataset has been deposited in the Gene Expression Omnibus data repository (GSE335471).

### Immunohistochemistry

Tissues were fixed in 4% paraformaldehyde at 4°C overnight and incubated in 30% sucrose (Sigma-Aldrich) overnight at 4°C. Samples were embedded in optimal cutting temperature compound (Tissue-Tek), frozen and cryosectioned (10 µm). Sections were washed in PBS, blocked (5% goat serum and 0.25% Triton X-100 in PBS) for 1 h at room temperature and incubated with primary antibodies overnight at 4°C. After washing, sections were incubated with secondary antibodies (Jackson ImmunoResearch), counterstained with DAPI and mounted in Vectashield Plus Antifade Mounting Medium (Vector Laboratories, H-1900-2). Images were acquired using an AxioScan Z.1 microscope (Zeiss) and analyzed with ImageJ.

### ELISA

Serum testosterone levels were measured using a testosterone ELISA kit (Crystal Chem, 80552) following the manufacturer’s instructions. For IL-15 Cx measurements, individual ingLNs were weighed and homogenized in 100 µl of RIPA buffer. Fifty microliters of lysate per sample were used for ELISA (Thermo Fisher Scientific, BMS6023) according to the manufacturer’s protocol.

### PCR to determine sex from genomic DNA

Genetic sex of tumor cell lines was determined as previously described^74^. DNA was extracted (DNA extraction columns from Zymo Research) from cultured tumor cells (B16F10, female controls: 4T1, Yumm3.3) or splenocytes from male mice. PCR was performed using primer pairs listed in Supplementary Table 2 with Hot Start Taq Plus polymerase (Qiagen, 203605). The following parameters were used: 95 °C for 2 min, 35 cycles of 94 °C for 30 s, 56 °C (*SX* and *Ube* primers) and 55 °C (*Zfy* primers) for 30 s and 72 °C for 30 s, followed by final elongation at 72 °C for 5 min. PCR products were resolved on 2% agarose gels and visualized with GelRed (Biotium).

### sjTREC PCR detection in mice

Genomic DNA from LN cells was extracted using the DNeasy Blood & Tissue Kit (Zymo Research) per the manufacturer’s instructions. qPCR with primers and probe specific for sjTREC (Supplementary Table 2) was performed using PrimeTime Gene Expression Master Mix (IDT, 1055770). The following parameters were used: 95 °C for 3 min, 45 cycles of 95 °C for 15 s and 60 °C for 60 s, followed by a final elongation at 72 °C for 5 min. Standard curves were generated using a plasmid containing the sjTREC sequence (a gift from K. Gustafsson, MGH). sjTREC copy number per microgram of DNA was calculated from the standard curve.

### Real-time PCR

LNs were homogenized in 100 µl of RLT lysis buffer. mRNA was extracted using the RNeasy plus kit (Qiagen) according to the manufacturer’s instructions. RNA reverse transcription was performed using an iScript cDNA synthesis kit (Bio-Rad, 1708890). Real-time PCR mix with SYBR Green was purchased from Bio-Rad Laboratories. The primers for real-time PCR are listed in Supplementary Table 2.

### TCR sequencing

CD8^+^ T cells were isolated from individual ingLNs using the EasySep Mouse CD8^+^ T Cell Isolation Kit (StemCell, 19753). Sorted cells were submitted to iRepertoire for TCR sequencing and bioinformatic analysis. This included RNA isolation, reverse transcription, amplification of *TRA* and *TRB* CDR3 regions and sequencing to assess T cell clonality and diversity.

#### Shannon entropy

Shannon entropy was calculated to determine TCR diversity. The frequency of each clonotype was used to compute probabilities (*p*_i_ = frequency_i/*N*_total), and entropy (*H*) was calculated as

$$H=\mathop{\sum }\limits_{i=1}^{N}{p}_{i}{\log }_{2}{p}_{i}$$

where *N* is the total number of unique clonotypes.

#### Shannon entropy approximations

The average values of total TCRβ (TRB) and unique TRB of all six samples (three female and three male mice) were used to calculate theoretical values of samples with similar characteristics and a certain diversity (100% and 50% unique TCRs). Entropy was computed using the formula above. Probabilities for unique clonotypes were calculated as *p*_unique_ = 1 / *N*_total_, whereas probabilities for expanded clonotypes were based on their observed frequencies. The total entropy was obtained by summing the contributions from unique and expanded clonotypes.

(Case A, 100%) maximum diversity means each sequence is unique and appears exactly once, calculated with *H*=log_2_*N*;

(Case B, 50%, with a single highly expanded clonotype):

$${H}_{{\rm{unique}}}=-{N}_{{\rm{unique}}}\times {p}_{{\rm{unique}}}\times {\log }_{2}{p}_{{\rm{unique}}}$$

equation for expanded clonotypes: $${H}_{{\rm{expanded}}}=-{p}_{{\rm{expanded}}}$$$$\times {\log }_{2}{p}_{{\rm{expanded}}}$$

equation for combined entropy: ${H}_{{\rm{total}}}={H}_{{\rm{unique}}}+{H}_{{\rm{expanded}}}$

(Case C, 50%, with duplicates evenly distributed among ten expanded clonotypes):

$${N}_{{\rm{expanded}}}={N}_{{\rm{unique}}}/10$$

$${P}_{{\rm{expanded}}}={N}_{{\rm{expanded}}}/{N}_{{\rm{total}}}$$

$${H}_{{\rm{unique}}}=-{N}_{{\rm{unique}}}\times {p}_{{\rm{unique}}}\times {\log }_{2}{p}_{{\rm{unique}}}$$

$${H}_{{\rm{expanded}}}=-10\times {p}_{{\rm{expanded}}}\times {\log }_{2}{p}_{{\rm{expanded}}}$$

$${H}_{{\rm{total}}}={H}_{{\rm{unique}}}+{H}_{{\rm{expanded}}}$$

### Reanalysis of spectral flow data

Spectral flow data from Bohacova et al.^36^ (Synapse: syn53238646) were analyzed using the gating strategy described in Supplementary Fig. 5, with the aim to quantify true naive CD8^+^ and CD4^+^ cells and with an emphasis to exclude all non-true naive T cells that might interfere with the subsequent quantification of RTEs^36^. The following markers were used in live cells: CXCR5^−^CD19^−^ (excludes B cells and T follicular helper cells), CD14^−^CD123^−^ (excludes myeloid cells), NKp80^−^CD56^−^ (excludes natural killer cells), TCRγδ^−^CD3^+^ (excludes γδT cells, includes all other T cells), gating on CD8^+^ T cells (CD8^+^CD4^−^): CD25^−^PD-1^−^FAS^−^GZMB^−^ (excludes cells expressing activation markers), CD27^+^CD45RA^+^ (true naive CD8^+^ T cells), CD38^high^ (RTE). CD4^+^ T cells (CD8^−^CD4^+^): FAS^−^CXCR3^−^ (excludes cells expressing activation markers), CD127^+^CD25^−^ (excludes regulatory T cells), CD27^+^CD45RA^+^ (true naive CD8^+^ T cells), CD38^high^ (RTE). Data were analyzed with FlowJo (v.10.1, TreeStar).

### Reanalysis of scRNA-seq data

All scRNA-seq data were analyzed with Seurat (v.5).

Human PBMC public scRNA-seq data^35^ from Terekhova et al.^35^ (Synapse: syn49637038) were filtered for CD8^+^ T cells and assigned a ‘Naive score’ using the UCell package with positive and negative modulator genes (*LEF1*^+^, *TCF7*^+^, *KLF2*^+^, *BACH2*^+^, *FOXP1*^+^, *GZMB*^−^, *PRDM1*^−^, *TBX21*^−^, *FAS*^−^, *EOMES*^−^).

Human LN cells from public scRNA-seq data^75^ were filtered for CD8^+^ T cells and assigned a ‘Naive score’ as described above. APCs from the same dataset were analyzed using the cell subset attribution from the original publication. scTCR-seq data from T cells from LNs of the same dataset were analyzed using the scRepertoire^76^ (v2.3.0) package in R.

Human melanoma datasets were obtained from Durante et al.^20^ (GSE139829), Sade-Feldman et al.^21^ (GSE120575), Li et al.^22^ (GSE123139) and Zhang et al.^23^ (GSE215121). Cells from individual participants were filtered for CD8^+^ T cells, and the percentage of naive, cytotoxic and exhausted CD8^+^ T cells was attributed to module scores using the UCell package (naive: *SELL*, *CCR7*; cytotoxic: *GZMA*, *GZMB*, *PRF1*, *IFNG*, *PTPN6*, *GNLY*; exhausted: *HAVCR2*, *TOX*, *TIGIT*).

Human thymocyte public scRNA-seq data from Park et al.^46^ (https://doi.org/10.5281/zenodo.3711134)^77^ and Cordes et al.^47^ (GSE195812) were assigned for subsets using the Ucell package with positive and negative modulator genes (double-negative 1: *CD34*^+^, *HES1*^+^, *IL7R*^+^, *TRAC*^−^; double-negative 2: *HES1*^+^, *PTCRA*^+^, *TRAC*^−^; double-negative 3: *NOTC1*^+^, *PTCRA*^+^, *CD8A*^−^, *CD4*^−^; double-negative 4: *RAG1*^+^, *BCL11B*^+^, *PTCRA*^+^, *CD8A*^−^, *CD4*^−^; double-positive: *RAG1*^+^, *CD8A*^+^, *CD4*^+^; single-positive CD8: *CD8A*^+^, *CD8B*^+^, *TRAC*^+^, *CD4*^−^; single-positive CD4: *TRAC*^+^, *CD4*^+^; regulatory T cell: *FOXP3*^+^, *IL2RA*^+^, *IKZF2*^+^; mature naive: *CCR7*^+^, *TRAC*^+^, *FOXP1*^+^, *KLF2*^+^, *CD8A*^+^, *CD4*^+^, *CD69*^−^, *IL2RA*^−^; γδ T cells: *TRDC*^+^, *SOX13*^+^, *TRAC*^−^; *CD8A*^−^, *CD8B*^−^, *CD4*^−^.

### Immune scores

Immune scores were derived from transcriptomic data of melanoma tumors from The Cancer Genome Atlas using the Estimation of Stromal and Immune cells in Malignant Tumor tissues using Expression data platform developed at MD Anderson Cancer Center, University of Texas.

### Statistics and reproducibility

Groups were compared using Prism software (GraphPad, v10). The statistical methods used are described in the figure legends and include two-sided MWU, unpaired two-sided *t-*test, two-sided log-rank test, two-way ANOVA, two-sided Fisher’s exact test, two-sided Spearman correlation and two-sided Pearson correlation. Tumor growth curves were analyzed using two-way repeated measures ANOVA with Geisser–Greenhouse correction, with the interaction between time and group used to assess differences in growth dynamics between experimental groups. Multiple pairwise comparisons were not performed unless explicitly indicated. Data distribution was assumed to be normal, but this was not formally tested. In mouse experiments, each data point represents an independent biological replicate from an individual mouse or an individual tissue (for example, an LN, tumor, blood sample or thymus) obtained from one mouse, as specified in the figure legends. In the SCAPIS data, a sex effect controlling for age was evaluated using a general linear model. Statistical significance is indicated in the figures as *P* values. No statistical methods were used to predetermine sample sizes, but our sample sizes are similar to those reported in previous publications^73^. No data were excluded from the analyses. Mice were assigned to experimental groups within each sex and age group by simple randomization. The investigators were not blinded to allocation during experiments or outcome assessment.

### Reporting summary

Further information on research design is available in the Nature Portfolio Reporting Summary linked to this article.

## Supplementary information

Supplementary Information Supplementary Figs. 1–7 and Tables 1 and 2.

Reporting Summary

Peer Review File

## Source data

Source Data Fig. 1 Statistical source data.

Source Data Fig. 2 Statistical source data.

Source Data Fig. 3 Statistical source data.

Source Data Fig. 4 Statistical source data.

Source Data Fig. 5 Statistical source data.

Source Data Fig. 6 Statistical source data.

Source Data Extended Data Fig. 1 Statistical source data.

Source Data Extended Data Fig. 2 Statistical source data.

Source Data Extended Data Fig. 3 Statistical source data.

Source Data Extended Data Fig. 4 Statistical source data.

Source Data Extended Data Fig. 5 Statistical source data.

Source Data Extended Data Fig. 6 Statistical source data.

Source Data Extended Data Fig. 7 Statistical source data.

Source Data Extended Data Fig. 8 Statistical source data

Source Data Extended Data Fig. 9 Statistical source data.

Source Data Extended Data Fig. 10 Statistical source data.

## Data Availability

All data supporting the findings of this study are available in the main text, the Supplementary Information and from the corresponding authors. The bulk RNA and TCR sequencing datasets have been deposited in the Gene Expression Omnibus under accession numbers GSE335471 and GSE344576. Source data are provided with this paper.
