# Supplementary material for: Lymph node contraction links sex-biased naive CD8+ T cell decline to compromised antigen recognition during middle age
Source: Nat Aging. 2026 Oct 9;6(10):2126–42. doi: 10.1038/s43587-026-01238-4 (PMC13661286; doi:10.1038/s43587-026-01238-4)
Supplement: Supplementary file 1 [file 43587_2026_1238_MOESM1_ESM.pdf]

nature aging

Supplementary information https://doi.org/10.1038/s43587-026-01238-4

Lymph node contraction links sex-biased naive CD8^{+} T cell decline to compromised antigen recognition during middle age

In the format provided by the authors and unedited

Supplementary Information

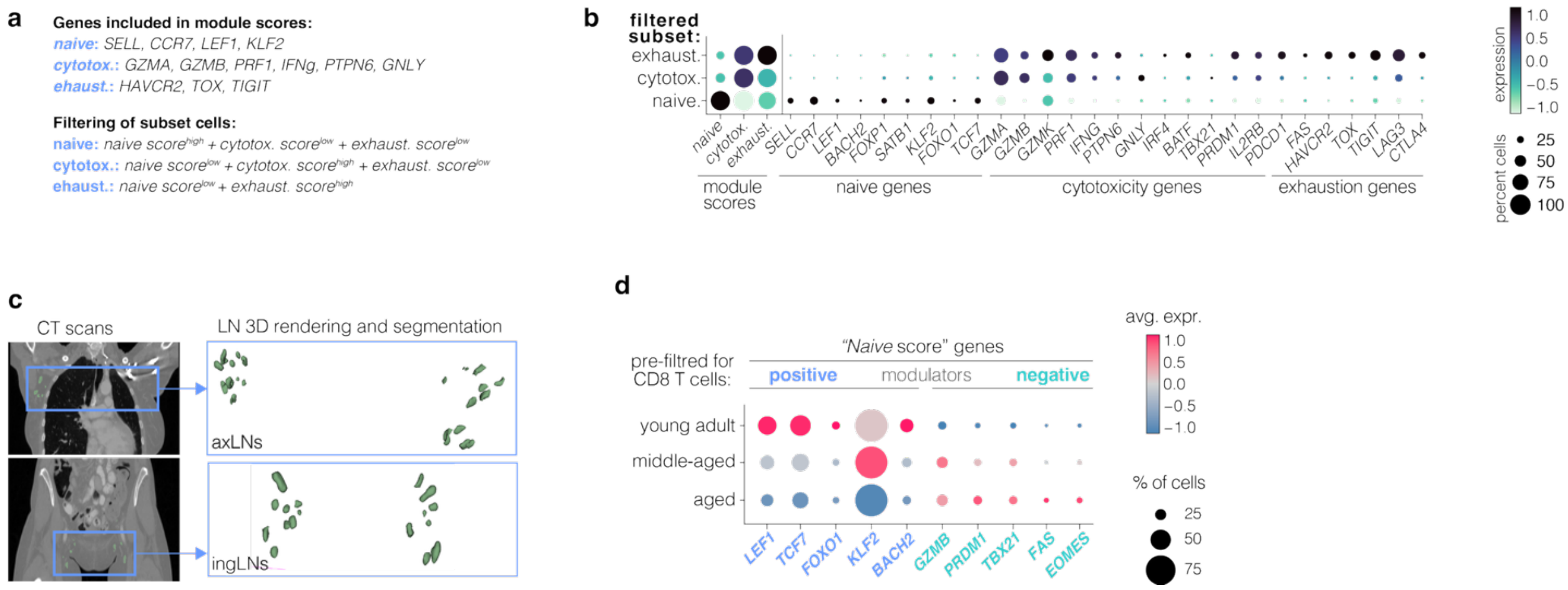

Supplementary Fig. 1. a, Gene sets used to compute module scores for naive, cytotoxic, and exhausted CD8 T cell states. Integration of these scores was used to classify CD8 T cell subsets within tumor datasets. **b,** Module score distributions and expression of canonical marker genes for each filtered CD8 T cell subset. Integrated data from Durante *et al.*[1], Sade-Feldman *et al.*[2], Li *et al.*[3], Zhang *et al.*[4]. **c**, Schematic representation of LN segmentation, 3D rendering, and quantification of LN volumes based on CT scans from Roth *et al.* and Rister *et al.*[5,6]. **d**, Expression levels of genes included in the "*Naive Score*" with genes that positively or negatively modulate the score.

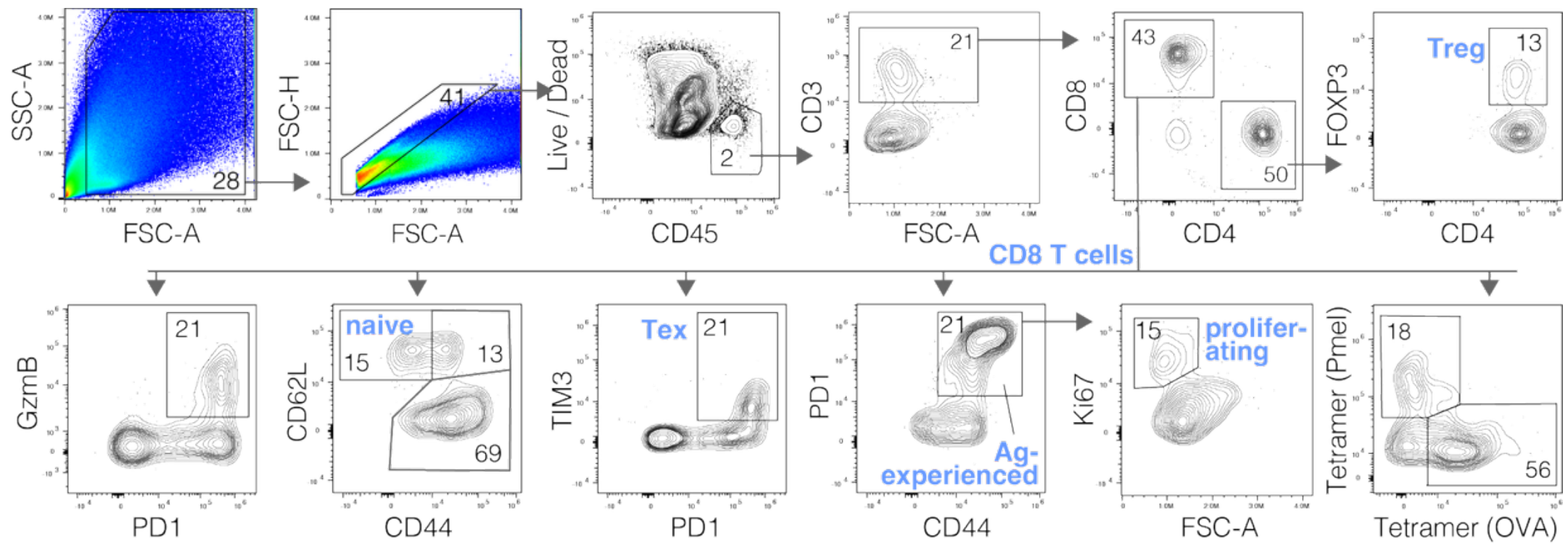

Supplementary Fig. 2. a, Flow cytometry gating strategy of lymphocytes in the primary tumor; regulatory T cells (Treg), exhausted T cells (Tex).

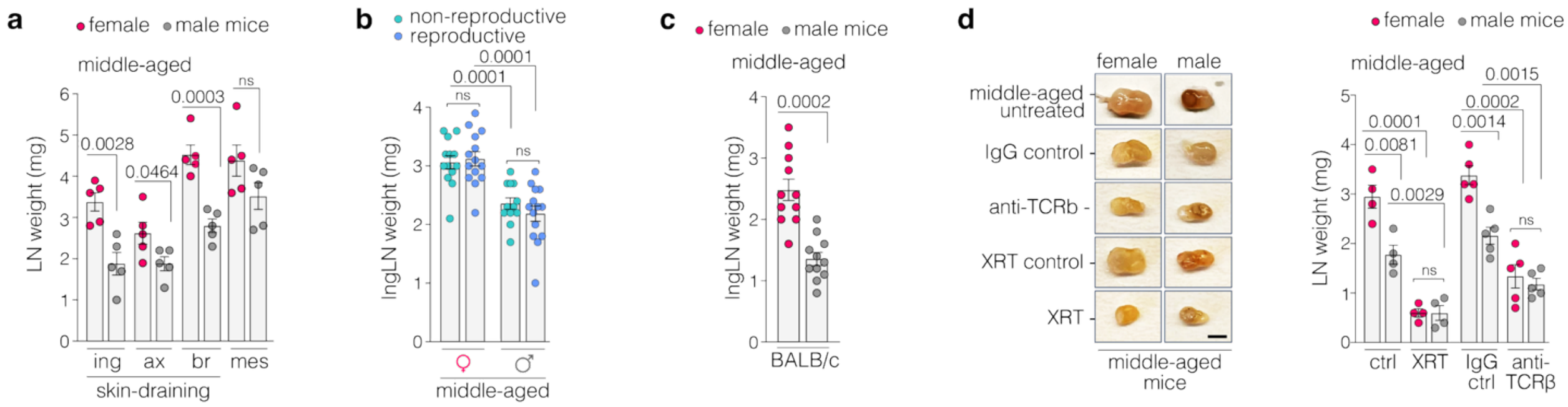

Supplementary Fig. 3. a, LN weights in different age groups of female and male mice, including inguinal (ing), axillary (ax), brachial (br), and mesenteric (mes) LNs. **c,** Weights of inguinal LNs in non-reproductive and reproductive (retired breeder) female and male mice. **d,** LN weights in middle-aged female and male BALB/c mice. **e,** Representative photographs and weight quantification of inguinal LNs from middle-aged mice treated with irradiation (XRT, sublethal dose of 4 Gy) or anti-TCRβ antibody-mediated T cell depletion. Scale bar 200 µm. Data shown as mean ± SEM with data points representing single LNs of individual animals. Statistics calculated with MWU test (b,c) or unpaired two-sided t test (a,d).

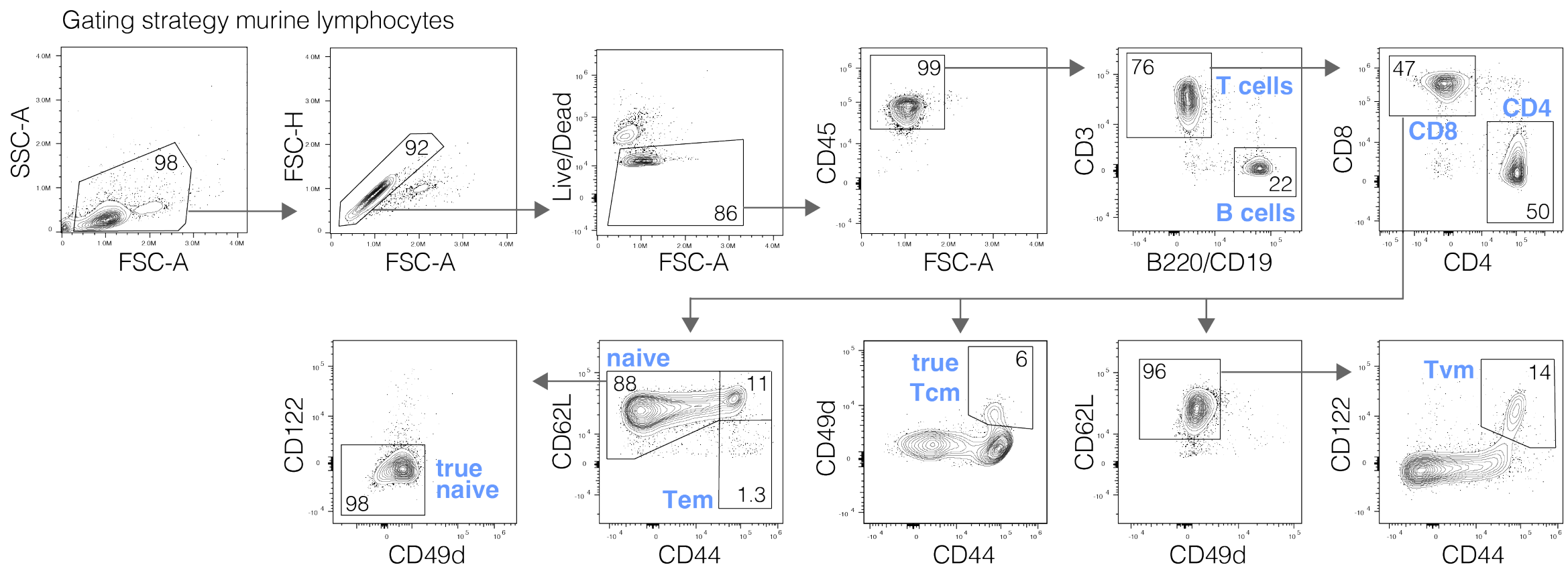

Supplementary Fig. 4. Flow cytometry gating strategy for murine lymphocytes, including central memory T cells (Tcm), effector memory T cells (Tem), and virtual memory T cells (Tvm).

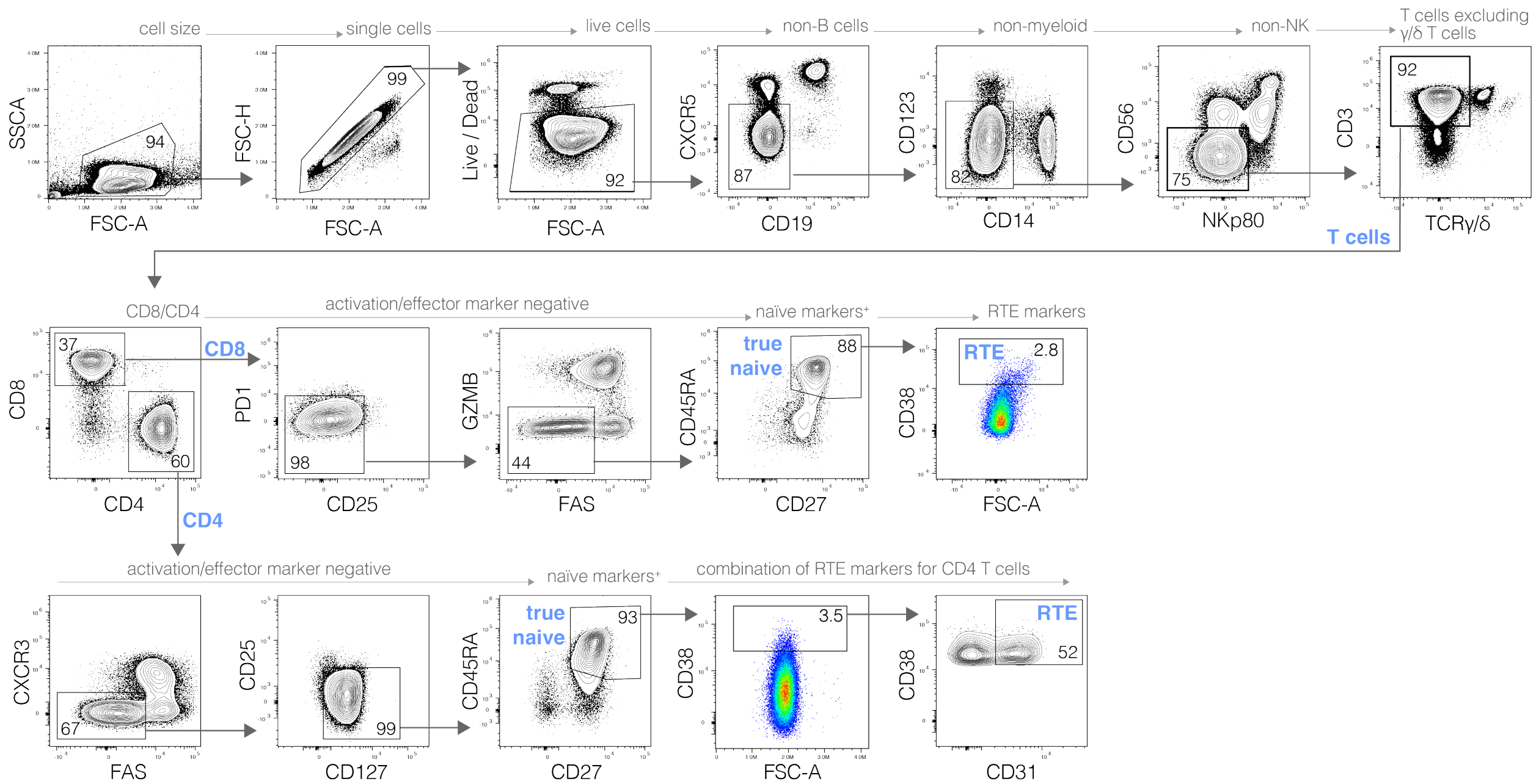

Supplementary Fig. 5. Flow cytometry gating strategy for human PBMCs, including recent thymic emigrants (RTEs), with data derived from Bohacova & Terekhova *et al.*[7].

a

t = *time step* (1 time step = 3 weeks = month x 4.345/3)
N_t = fraction of naive cells remaining at time t
k_{sex} = conversion probability per step (sex specfic)
C_t = fraction converted into Tvm
$s_{sex}(t)$ = thymic input at time t

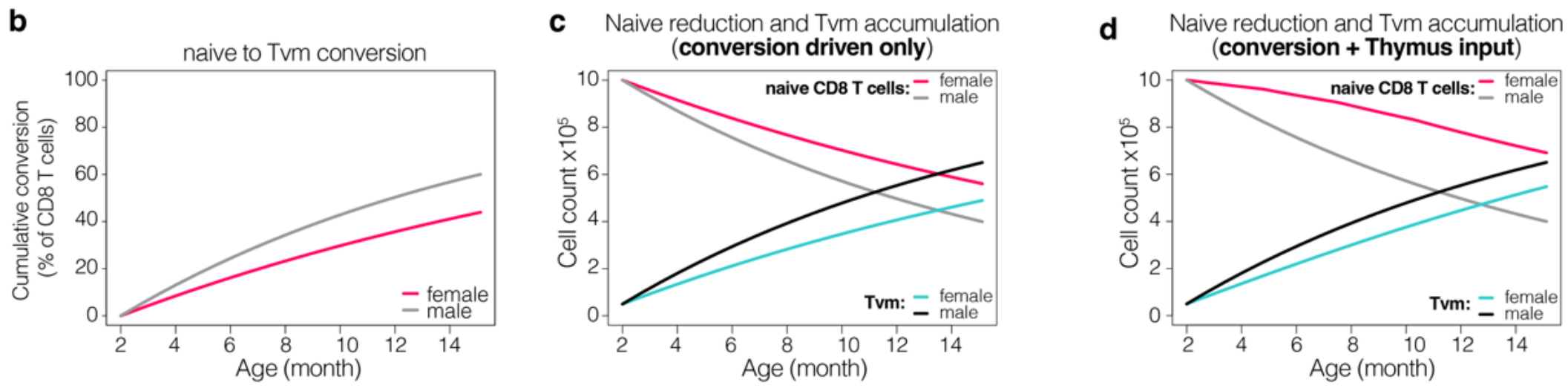

Core assumptions (b):
- fixed fraction (k) of naive converts per time step
- no replenishment of naive T cells
- V_0 = 5x10^5 Tvm cells
- k_{female} = 0.03
- k_{male} = 0.03 × (1.1 / 0.7) = 0.047

$N_{t+1} = N_t \times (1-k)$
$C_t = 1-(1-k)^t$
Conversion (%) $= 100 \times (1-(1-k)^t)$
$k_{female} = 0.03$
$C_{female}(t) = 1-(1-0.03)^t$
$k_{male} = 0.03 \times (1.1 / 0.7) = 0.047$
$C_{male}(t) = 1 - (1 - 0.047)^t$

Core assumptions (c):
- fixed fraction (k) of naive converts per time step
- no replenishment of naive T cells
- Tvm conversion 1:1 naive reduction
- N_0 = 10^6 naive cells
- V_0 = 5x10^5 Tvm cells
- k_{female} = 0.03
- k_{male} = 0.03 × (1.1 / 0.7) = 0.047

$N_{t+1} = N_t \times (1-k)$
$V_t+1 = V_t + k_{sex} \times N_t$
naive pool: $N_t = N_0 \times (1-k)^t$
Tvm pool: $V_t = V_0 + N_0 \times (1-(1-k)^t)$
$N_t + V_t = N_0 + V_0$

Core assumptions (d):
- fixed fraction (k) of naive converts per time step
- Tvm conversion 1:1 naive reduction
- Replenishment of naive T cells (thymus input)
- N_0 = 10^6 naive cells
- V_0 = 5x10^5 Tvm cells
- k_{female} = 0.03
- k_{male} = 0.03 × (1.1 / 0.7) = 0.047
- s_{female} = 20,000 (t≤4); s_{male}×0.7 (4<t≤8); s_{male}×0.4 (8<t≤12); s_{male}×0.15 (t>12)
- s_{male} = 5,000 (t≤4); s_{male}×0.3 (4<t≤8); s_{male}×0.15 (8<t≤12); s_{male}×0 (t>12)

$N_{sex}(t+1) = N_{sex}(t) + s_{sex}(t) - k_{sex} \times N_{sex}(t)$
$V_{sex}(t+1) = V_{sex}(t) + k_{sex} \times N_{sex}(t)$

Supplementary Fig. 6. Conceptual framework for cumulative naive CD8 T cell to virtual memory T cell conversion. a, Definition of parameters. **b, *Cumulative conversion over time.*** To assess whether the observed sex differences in conversion propensity are sufficient to account for progressive divergence in naive CD8 T cell maintenance, we implemented a minimal discrete-time model of cumulative naive-to-Tvm conversion based on models of compounding. In this framework, a fixed fraction k of the remaining naive pool converts to Tvm cells per time interval, such that the naive fraction evolves according to $N_{t+1} = N_t(1 - k)$, yielding $N_t = (1 - k)^t$. The cumulative fraction of converted cells is therefore $C_t = 1 - (1 - k)^t$. Based on published adoptive transfer experiments (White et al., *Nature Communications* 2016), we set k_{female} = 0.03 per 3-week interval for females. To account for the increased frequency of conversion-prone cells in male subjects, we scaled this rate by the observed ratio of CD5^{high} naive CD8 T cells **(Fig. 3f)**, yielding k_{male} 0.03*(1.1/0.7) = 0.047. This minimal model demonstrates that modest differences in per-interval conversion rates produce substantial sex-biased divergence in cumulative conversion over time. **c*, Naive reduction and Tvm accumulation (conversion driven only).*** We modeled naive CD8 T cell decline as a discrete-time process in which a fixed fraction k of the naive pool converts to Tvm cells per interval, in the absence of thymic input. This yields $N_{t+1} = N_t(1 - k)$ and $V_{t+1} = V_t + k*N_t$, resulting in exponential decay of the naive pool and corresponding cumulative accumulation of Tvm cells. **d*, Naive reduction and Tvm accumulation (conversion + Thymic input).*** To incorporate sex-biased thymic support, we extended the conversion model by adding a time-dependent thymic input term to the naive compartment. Naive CD8 T cell dynamics were modeled as $N_{t+1} = N_t + s_t - k*N_t$, with corresponding Tvm accumulation given by $V_{t+1} = V_t + k*N_t$. Thymic input was modeled as a gradually declining function s with more sustained thymic input in female compared to male subjects.

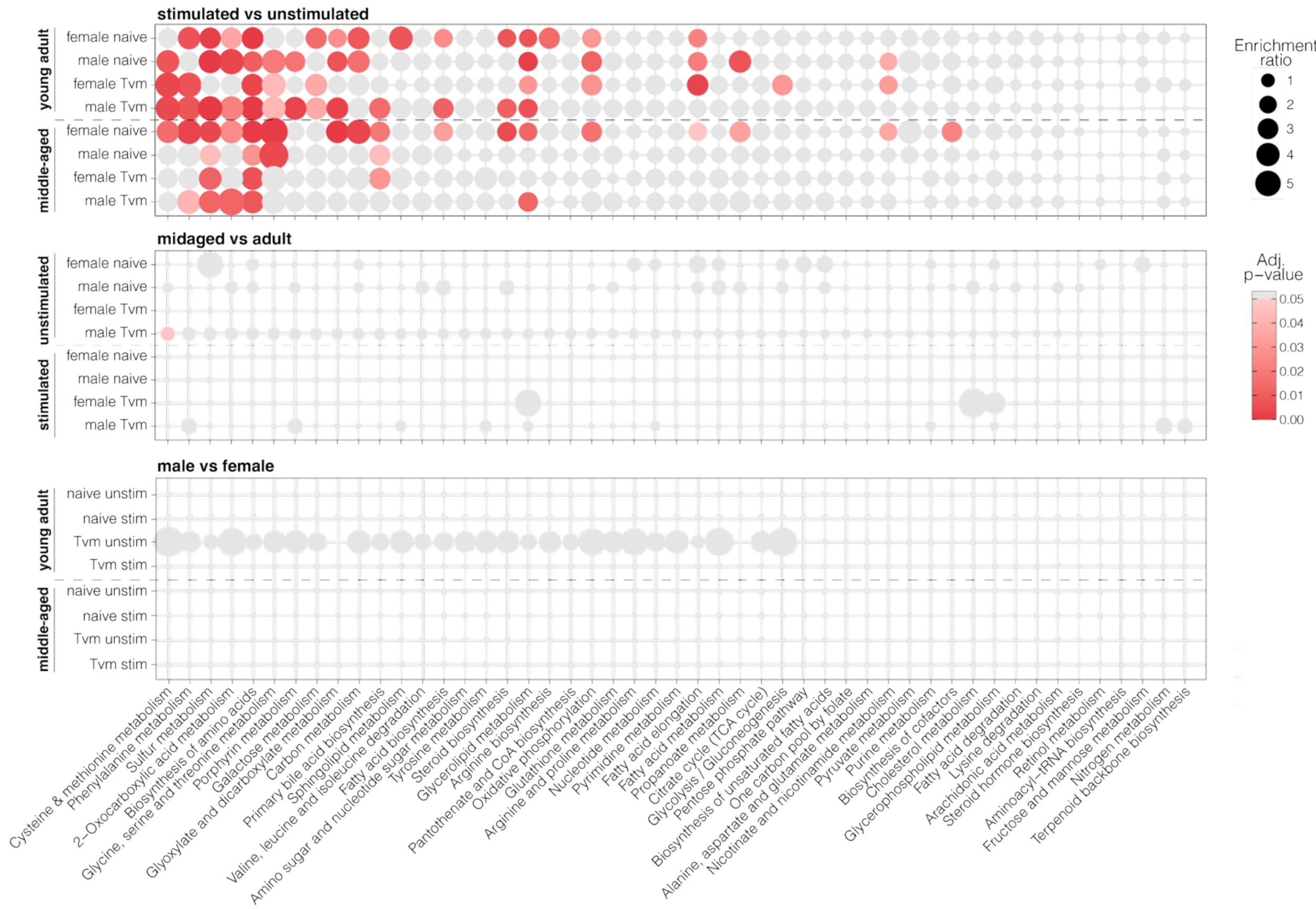

Supplementary Fig. 7. KEGG metabolic pathway enrichment analysis. Tvm and naive CD8 T cells from young adult and middle-age female and male mice were analyzed after 5 hours of CD3/CD28 in vitro stimulation (n=3/group). Dot size indicates enrichment ratio: (differentially expressed genes in pathway / total differentially expressed genes genes) / (all genes in pathway / all genes in genome); color indicates FDR-adjusted p-value (grey = not significant, red = adj. $p < 0.05$). Statistics calculated with BH-corrected hypergeometric test p-value.

Supplementary Table 1. Antibodies, tetramers and dyes.

Epitope	Fluorophore	Reactivity	Manufacturer	Clone	Cat. #	RRID
Annexin-V	PE		BioLegend		640907	
B220	AF647	mouse	BioLegend	RA3-6B2	103229	AB_389330
B220	APC/Cy7	mouse	BioLegend	RA3-6B2	103223	AB_313006
B220	PE	mouse	BioLegend	RA3-6B2	103207	AB_312992
CD11b	AF 700	mouse	BioLegend	M1/70	101222	AB_493705
CD11c	APC	mouse	BioLegend	N418	117309	AB_313778
CD122	PE	mouse	BioLegend	TM-β1	123209	AB_940617
CD122	PE/Cy5	mouse	BioLegend	TM-β1	123219	AB_2715961
CD127	PE/Cy5	mouse	BioLegend	A7R34	135015	AB_1937261
CD127	FITC	mouse	BioLegend	A7R34	135007	AB_1937231
CD16/CD32	purified	mouse	BioLegend	93	101301	AB_312800
CD19	FITC	mouse	BioLegend	6D5	115505	AB_313640
CD19	AF700	mouse	BioLegend	1D3/CD19	152414	AB_2922474
CD25	PE	mouse	BioLegend	PC61	102007	AB_312856
CD3	FITC	mouse	BioLegend	17A2	100203	AB_312661
CD3	PE/Dazzl494	mouse	BioLegend	17A2	100246	AB_2565882
CD3	PerCP	mouse	BioLegend	17A2	100217	AB_1595492
CD39	PE/Cy7	mouse	BioLegend	Duha59	143805	AB_2563393
CD4	APC/Cy7	mouse	BD Bioscience	GK1.5	561830	AB_10897172
CD4	BV605	mouse	BioLegend	RM4-5	100547	AB_11125962
CD4	BV650	mouse	BioLegend	RM4-5	100551	AB_2563053
CD4	APC	mouse	BioLegend	RM4-5	100515	AB_312718
CD44	APC	mouse	BioLegend	IM7	103011	AB_312962
CD44	BV750	mouse	BioLegend	IM7	103037	AB_10900641
CD44	PE	mouse	BioLegend	IM7	103023	AB_312958
CD45	BV510	mouse	BioLegend	30-F11	103137	AB_2561392
CD45	PE/Dazzl493	mouse	BioLegend	30-F11	103145	AB_2564002
CD45	AF700	mouse	BioLegend	30-F11	103127	AB_493714
CD45	PE	mouse	BioLegend	30-F11	103105	AB_312970
CD45	PerCP	mouse	BioLegend	30-F11	103129	AB_893343
CD49d	FITC	mouse	BioLegend	R1-2	103605	AB_313036
CD49d	PE	mouse	BioLegend	R1-2	103607	AB_313039
CD5	AF700	mouse	BioLegend	53-7.3	100635	AB_2687001
CD62L	PE	mouse	BioLegend	MEL-14	104407	AB_313094

CD62L	BV421	mouse	BioLegend	MEL-14	104435	AB_10900082
CD62L	AF700	mouse	BioLegend	MEL-14	104426	AB_493719
CD69	FITC	mouse	BioLegend	H1.2F3	104505	AB_313108
CD69	AF700	mouse	BioLegend	H1.2F3	104539	AB_2566304
CD8a	Spark-UV387	mouse	BioLegend	53-6.7	100797	AB_2922451
CD8a	PE/Cy7	mouse	BioLegend	53-6.7	100721	AB_312760
CD8a	Spark-YG593	mouse	BioLegend	QA17A07	155023	AB_2892316
CD8a	FITC	mouse	BioLegend	53-6.7	100705	AB_312744
CD8a	PE	mouse	BioLegend	53-6.7	100707	AB_312747
F4/80	PerCP/Cy5.5	mouse	BioLegend	BM8	123127	AB_893484
FoxP3	PerCP/Cy5.5	mouse	eBioscience	FJK-16s	45577382	AB_914351
Gp100 (Pmel)	PE	mouse	abcam	EP4863(2)	ab246731	AB_2732921
Ki67	BV650	mouse	BioLegend	11F6	151215	AB_2876504
Ki67	PE/Cy7	mouse	BD Bioscience	B56	561283	AB_10716060
Ki67	BV510	mouse	BioLegend	B56	563462	AB_2738221
MHC-I:SIINFEKL	PE	mouse	BioLegend	25-D1.16	141603	AB_10895905
MHC-I:SIINFEKL	APC	mouse	BioLegend	25-D1.16	141605	AB_11219402
MHC-II	AF647	mouse	BioLegend	M5/114.15.2	107617	AB_493525
Nur77	AF594	mouse	BioLegend	1E10A15	653504	AB_2563321
Nur77	PE	mouse	Miltenyi	REA704	130111417	AB_2653071
PD1	BV785	mouse	BioLegend	29F.1A12	135225	AB_2563680
PD1	PE	mouse	BioLegend	29F.1A12	135205	AB_1877231
PD1	APC	mouse	BioLegend	29F.1A12	135209	AB_2159183
TCRb	BV711	mouse	BioLegend	H57-597	109243	AB_2629564
TCRb (InVivoMAb)	purified	mouse	BioXcell	H57-597	#BE0102	AB_10950158
Tetramer gp100 (25-33)	AF647	mouse	NTCF (NIH)	EGSRNQDWL	#71998	
Tetramer OVA (257-264)	BV421	mouse	NTCF (NIH)	SIINFEKL	#73246	
Tim-3	BV711	mouse	BioLegend	RMT3-23	119727	AB_2716208
Tim-3	APC/Fire750	mouse	BioLegend	RMT3-23	119737	AB_2810367
Zombie-Red Live Dead			BioLegend		423109	
Zombie-UV Live Dead			BioLegend		423108	

Gzmb	APC	mouse	BioLegend	QA16A02	372203	AB_2687027
Gzmb	FITC	mouse	BioLegend	QA16A02	372205	AB_2687029
B220	BV711	mouse	BioLegend	RA3-6B2	103255	AB_2563491
IL-2	PerCP/Cy5.5	mouse	Biolegend	JES6-5H4	503821	AB_961403
IFNg	APC	mouse	Biolegend	XMG1.2	505809	AB_315403
TNFa	FITC	mouse	Biolegend	MP6-XT22	506303	AB_315424
CD3	purified	mouse	Biolegend	145-2C11	100340	AB_2616673
CD28	purified	mouse	Biolegend	37.51	102115	AB2810330

Supplementary Table 2. Antibodies, tetramers and dyes.

**Gene**	**5'-...-3'**	**Sequence**	**Source**
sjTREC (mouse)	primer fw.:	CCAAGCTGACGGCAGGTTT	Gustafsson *et al.* [14]
	primer rev.:	AGCATGGCAAGCAGCACC	
	probe:	FAM/TGCTGTGTG/ZEN/CCCTGCCCTGCC/3IABkFQ	
SX (mouse)	primer fw.:	GATGATTTGAGTGGAAATGTGAGGTA	McFarlane *et al.*[15]
	primer rev.:	CTTATGTTTATAGGCATGCACCATGTA	
Zfy (mouse)	primer fw.:	GACTAGACATGTCTTAACATCTGTCC	McFarlane *et al.*[15]
	primer rev.:	CCTATTGCATGGACTGCAGCTTATG	
Ube (mouse)	Primer Fw.:	TGGTCTGGACCCAAACGCTGTCCACA	McFarlane *et al.*[15]
	Primer Rev.:	GGCAGCAGCCATCACATAATCCAGATG	
TREC (human)	Primer Fw.:	CACATCCCT TTCAACCATGCT	Sandstedt et al. [16]
	Primer Rev.:	GCCAGCTGCAGGGTTTAG G	
	Probe:	ACACCTCTGTTTTTGTAAAGGTGC CCACT	
Il15 (mouse)	primer fw.:	ACATCCATCTCGTGCTACTTGT	PrimerBank
	primer rev.:	GCCTCTGTTTTAGGGAGACCT	PrimerBank

Supplementary references

1. Durante, M. A. *et al.* Single-cell analysis reveals new evolutionary complexity in uveal melanoma. *Nat Commun* **11**, 496 (2020).
2. Sade-Feldman, M. *et al.* Defining T Cell States Associated with Response to Checkpoint Immunotherapy in Melanoma. *Cell* **175**, 998-1013.e20 (2018).
3. Li, H. *et al.* Dysfunctional CD8 T Cells Form a Proliferative, Dynamically Regulated Compartment within Human Melanoma. *Cell* **176**, 775-789.e18 (2019).
4. Zhang, C. *et al.* A single-cell analysis reveals tumor heterogeneity and immune environment of acral melanoma. *Nat Commun* **13**, 7250 (2022).
5. Roth, H. R. *et al.* A New 2.5D Representation for Lymph Node Detection using Random Sets of Deep Convolutional Neural Network Observations. *Med Image Comput Comput Assist Interv* **17**, 520–527 (2014).
6. Rister, B., Shivakumar, K., Nobashi, T., & Rubin, D. L. CT-ORG: ADataset of CT volumes with multiple organ segmentations. *The Cancer Imaging Archive (TCIA)* 10.7937/tcia.2019.tt7f4v7o (2019).
7. Bohacova, P. *et al.* Multidimensional profiling of human T cells reveals high CD38 expression, marking recent thymic emigrants and age-related naive T cell remodeling. *Immunity* **0**, (2024).
