# Supplementary material for: Lymph node contraction links sex-biased naive CD8+ T cell decline to compromised antigen recognition during middle age
Source: Nat Aging. 2026 Oct 9;6(10):2126–42. doi: 10.1038/s43587-026-01238-4 (PMC13661286; doi:10.1038/s43587-026-01238-4)
Supplement: Supplementary file 2 [file 43587_2026_1238_MOESM2_ESM.pdf]

Corresponding author(s): Lutz Menzel, Timothy P. Padera

Last updated by author(s): Aug 21, 2026

Reporting Summary

Nature Portfolio wishes to improve the reproducibility of the work that we publish. This form provides structure for consistency and transparency in reporting. For further information on Nature Portfolio policies, see our Editorial Policies and the Editorial Policy Checklist.

Statistics

For all statistical analyses, confirm that the following items are present in the figure legend, table legend, main text, or Methods section.

n/a	Confirmed	
☐	☒	The exact sample size (*n*) for each experimental group/condition, given as a discrete number and unit of measurement
☐	☒	A statement on whether measurements were taken from distinct samples or whether the same sample was measured repeatedly
☐	☒	The statistical test(s) used AND whether they are one- or two-sided<br>*Only common tests should be described solely by name; describe more complex techniques in the Methods section.*
☐	☒	A description of all covariates tested
☒	☐	A description of any assumptions or corrections, such as tests of normality and adjustment for multiple comparisons
☐	☒	A full description of the statistical parameters including central tendency (e.g. means) or other basic estimates (e.g. regression coefficient) AND variation (e.g. standard deviation) or associated estimates of uncertainty (e.g. confidence intervals)
☐	☒	For null hypothesis testing, the test statistic (e.g. *F*, *t*, *r*) with confidence intervals, effect sizes, degrees of freedom and *P* value noted<br>*Give P values as exact values whenever suitable.*
☒	☐	For Bayesian analysis, information on the choice of priors and Markov chain Monte Carlo settings
☒	☐	For hierarchical and complex designs, identification of the appropriate level for tests and full reporting of outcomes
☐	☒	Estimates of effect sizes (e.g. Cohen's *d*, Pearson's *r*), indicating how they were calculated

Our web collection on statistics for biologists contains articles on many of the points above.

Software and code

Policy information about availability of computer code

Data collection	Flow cytometry analysis was performed using a Cytek Aurora Spectral flow cytometer (5 lasers). Microscopy was performed using a AxioScan Z.1. qPCR was performed using Stratagene MXPro 3005 (Agilent). TCR sequencing was performed by the iRepertoire company.
Data analysis	Microscopy images were analyzed with Image J (Fiji, v.2.16.0). Flow cytometry data were analyzed with FlowJo (v.10, Treestar). Diagrams and statistical significances were generated using Prism10 (GraphPad). scRNA-seq data were analysed using R-studio (2025.05.1) and Seurat v.5.1.0. CT scans were analyzed using 3D Slicer (v.5.6.2)

For manuscripts utilizing custom algorithms or software that are central to the research but not yet described in published literature, software must be made available to editors and reviewers. We strongly encourage code deposition in a community repository (e.g. GitHub). See the Nature Portfolio guidelines for submitting code & software for further information.

Data

Policy information about availability of data

All manuscripts must include a data availability statement. This statement should provide the following information, where applicable:

- Accession codes, unique identifiers, or web links for publicly available datasets
- A description of any restrictions on data availability
- For clinical datasets or third party data, please ensure that the statement adheres to our policy

All data are available in the main text or the supplementary materials.
The bulk mRNA and TCR sequencing data set has been deposited the Gene Expression Omnibus data repository (accession number: GSE335471, GSE344576).

Research involving human participants, their data, or biological material

Policy information about studies with human participants or human data. See also policy information about sex, gender (identity/presentation), and sexual orientation and race, ethnicity and racism.

Reporting on sex and gender	All experiments and analyses of murine and human data includes females and males of not stated otherwise.
Reporting on race, ethnicity, or other socially relevant groupings	not applicable
Population characteristics	not applicable
Recruitment	not applicable
Ethics oversight	not applicable

Note that full information on the approval of the study protocol must also be provided in the manuscript.

Field-specific reporting

Please select the one below that is the best fit for your research. If you are not sure, read the appropriate sections before making your selection.

☒ Life sciences ☐ Behavioural & social sciences ☐ Ecological, evolutionary & environmental sciences

For a reference copy of the document with all sections, see nature.com/documents/nr-reporting-summary-flat.pdf

Life sciences study design

All studies must disclose on these points even when the disclosure is negative.

Sample size	no formal sample size power calculation was performed, but standard sample size selection in the field of research.
Data exclusions	To ensure that LN measurements reflected non-pathologic anatomical variation, we excluded all scans showing radiologic evidence of lymphadenopathy or other abnormalities that could influence LN size.
Replication	Mouse experiments were performed using independent biological replicates (individual mice). Key findings were repeated with independent experiments or supported by independent complementary experiments where appropriate; exceptions are indicated in the corresponding figure legends.
Randomization	Mice were grouped for sex, age and randomly assigned to treatment groups when possible. No other randomization was performed.
Blinding	For lymph node volume segmentation and thymus health analysis in CT scans, experimentators were blinded for the age of individuals.

Reporting for specific materials, systems and methods

We require information from authors about some types of materials, experimental systems and methods used in many studies. Here, indicate whether each material, system or method listed is relevant to your study. If you are not sure if a list item applies to your research, read the appropriate section before selecting a response.

Materials & experimental systems

n/a	Involved in the study
☐	☒ Antibodies
☐	☒ Eukaryotic cell lines
☒	☐ Palaeontology and archaeology
☐	☒ Animals and other organisms
☐	☒ Clinical data
☒	☐ Dual use research of concern
☒	☐ Plants

Methods

n/a	Involved in the study
☒	☐ ChIP-seq
☐	☒ Flow cytometry
☒	☐ MRI-based neuroimaging

Antibodies

Antibodies used	B220 (RA3-6B2, BioLegend, AB_389330, 1:100), CD11b (M1/70, BioLegend, AB_493705, 1:100), CD11c (N418, BioLegend, AB_313778, 1:200), CD122 (TM-β1, BioLegend, AB_940617, 1:200), CD127 (A7R34, BioLegend, AB_1937261, 1:100), CD16/CD32 (93, BioLegend, AB_312800, 1:100), CD19 (6D5, BioLegend, AB_313640, 1:200), CD25 (PC61, BioLegend, AB_312856, 1:200), CD3 (17A2, BioLegend, AB_312661, 1:200), CD39 (Duha59, BioLegend, AB_2563393, 1:200); CD4 (GK1.5, BioLegend, AB_10897172, 1:200); CD44 (IM7, BioLegend, AB_312962, 1:200); CD45 (30-F11, BioLegend, AB_2561392, 1:200); CD49d (R1-2, BioLegend, AB_313036, 1:100); CD5 (53-7.3, BioLegend, AB_2687001, 1:200), CD62L (MEL-14, BioLegend, AB_313094, 1:100); CD69 (H1.2F3, BioLegend, AB_313108, 1:200); CD8a (53-6.7, BioLegend, AB_2922451, 1:200); F4/80 (BM8, BioLegend, AB_893484, 1:100); FoxP3 (FJK-16s, eBioscience, AB_914351, 1:100); Gp100(Pmel) (EP4863(2), abcam, AB_2732921, 1:100); Ki67 (B56, BD, AB_10716060, 1:50); MHC-I:SIINFEKL (25-D1.16, BioLegend, AB_10895905); MHC-II (M5/114.15.2, BioLegend, AB_493525, 1:200); Nur77 (REA704, Miltenyi, AB_2653071, 1:50); Nur77 (1E10A15, BioLegend, AB_2563321, 1:50); PD1 (29F.1A12, BioLegend, AB_2563680, 1:200); TCRb (H57-597, BioLegend, AB_2629564, 1:100); TCRb(InVivoMAb) (H57-597, BioXcell, AB_10950158); Tim-3 (RMT3-23, BioLegend, AB_2716208, 1:200); Gzmb (QA16A02, BioLegend, AB_2687027, 1:100), IL-2 (JES6-5H4, Biolegend, 503821, AB_961403, 1:100), IFNg (XMG1.2, Biolegend, AB_315403, 1:100), TNFa (MP6-XT22, Biolegend, AB_315424, 1:100), CD3 purified (145-2C11, Biolegend, AB_2616673, 5 μg/ml), CD28 purified (37.51, Biolegend, AB2810330, 4 μg/ml)
Validation	All antibodies are established and well validated commercially available clones and routinely QC-checked by the manufacturer

Eukaryotic cell lines

Policy information about cell lines and Sex and Gender in Research

Cell line source(s)	B16F10 were originally purchased from ATCC (#CRL-6475). B16F10-OVA cells were a gift from Dr. Susan Thomas, Georgia Institute of Technology
Authentication	Cells were not authenticated but B16F10 and B16F10-OVA formed visible black melanoma tumors and were gp100(PMEL) positive in flow cytometry.
Mycoplasma contamination	Cells were routinely tested for mycoplasma contamination and remained negative throughout the study.
Commonly misidentified lines (See ICLAC register)	NA

Animals and other research organisms

Policy information about studies involving animals; ARRIVE guidelines recommended for reporting animal research, and Sex and Gender in Research

Laboratory animals	Wild type C57BL/6 mice, Lta-/- (lymphotoxin alpha knockout mice B6.129s2-Ltatm1Dch/J from the Jackson Laboratory, Strain# 002258) and OT-1 (C57BL/6-Tg(TcraTcrb)1100Mjb/J) were used for animal models.
Wild animals	not applicable
Reporting on sex	All experiments and analysis of murine and human data includes females and males of not stated otherwise.
Field-collected samples	not applicable
Ethics oversight	All experiments were performed at the Massachusetts General Hospital (MGH) and approved by the Institutional Animal Care and Use Committee (IACUC) of the MGH under the protocol numbers 2023N000101, 2011N000085 and 2004N000123.<br>The use of data from the SCAPIS study was approved by the appropriate regional ethics review board, and all participants provided written informed consent.

Note that full information on the approval of the study protocol must also be provided in the manuscript.

Clinical data

Policy information about clinical studies

All manuscripts should comply with the ICMJE guidelines for publication of clinical research and a completed CONSORT checklist must be included with all submissions.

Clinical trial registration	Swedish CArdioPulmonary bioImage Study (SCAPIS)
Study protocol	Bergström, G. et al. The Swedish CArdioPulmonary BioImage Study: objectives and design. J Intern Med 278, 645–659 (2015).
Data collection	Middle-aged participants (50–64 years old women and men) underwent comprehensive clinical examination including imaging and functional studies of the heart and lungs—that included the thymus—and completed extensive questionnaires to assess lifestyle as well as prior and current medication regiments. The SCAPIS Leukocyte subcohort of 1078 participants was consecutively recruited from the SCAPIS center at Linköping University Hospital to establish an immune cell profile in whole fresh blood using flow cytometry73. Twenty-nine participants (2.7%) were excluded from analysis: 9 (0.8%) due to missing images, 6 (0.6%) with anterior mediastinal lymphadenopathy or tumor, 5 (0.5%) with prior surgery preventing evaluation, 2 (0.2%) with imaging artefacts, and 7 (0.6%) for miscellaneous anatomical or technical reasons.
Outcomes	Age and Sex information, Thymus scores, naive CD8 T cell data from PBMCs

Plants

Seed stocks	not applicable
Novel plant genotypes	not applicable
Authentication	not applicable

Flow Cytometry

Plots

Confirm that:

☐ The axis labels state the marker and fluorochrome used (e.g. CD4-FITC).

☒ The axis scales are clearly visible. Include numbers along axes only for bottom left plot of group (a 'group' is an analysis of identical markers).

☒ All plots are contour plots with outliers or pseudocolor plots.

☒ A numerical value for number of cells or percentage (with statistics) is provided.

Methodology

Sample preparation	Mice were euthanized, and tissues were resected and mechanically dissociated. Single-cell suspensions from the thymus were filtered through a 40 μm strainer. Single-cell suspensions from tumor and spleen were filtered through a 70 μm strainer. Individual LNs were mechanically dissociated by gently pressing them with a syringe plunger against the surface of a sterile petri dish. Cells in the resulting suspension were counted before any filtering or centrifugation to ensure reliable cell counts without technical artifacts. For flow cytometry analysis of antigen presenting cells, LNs were treated with 1 mg/ml Collagenase-D (Sigma) and 0.1 mg/ml DNAse-I (Roche, in HBSS) for 30 min at 37°C before mechanical dissociation. Blood and spleen samples underwent hemolysis with ACK buffer (Thermo Fisher Scientific).<br>Single-cell suspensions were incubated with LIVE/DEAD Zombie dye (BioLegend) and anti-mouse CD16/32 in PBS for 15 min at 4°C to block Fc receptors and stain for viability. When applicable, tetramers were added for 1 hour at room temperature. Surface staining was then performed with fluorochrome-conjugated antibodies in flow buffer (2% FCS, 0.5 mM EDTA in PBS) for 20 min at room temperature
Instrument	Aurora (Cytek, 5 Lasers)
Software	SpectroFlow (v.3.1.2), FlowJo v.10
Cell population abundance	not applicable, no cell sorting (FACS) was performed
Gating strategy	For human PBMCs:<br>FSC-A/SSC-A; FSC-A/FSC-H; FSC-A/live cell marker negative: The following markers were used in live cells: CXCR5-/CD19- (exclude B cells, T follicular helper cells), CD14-/CD123- (exclude myeloid cells), NKp80-/CD56- (exclude NK cells), TCR γ/ δ -/

CD3+ (exclude γ/δ T cells, include all other T cells), gating on CD8 T cells: CD25-/PD1-/FAS-/GZMB- (exclude cells expressing activation markers), CD27+/CD45RA+ (true naive CD8 T cells), CD38high (RTE). CD4 T cells: FAS-/CXCR3- (exclude cells expressing activation markers), CD127+/CD25- (excluding regulatory T cells), CD27+/CD45RA+ (true naive CD8 T cells), CD38high (RTE).

Mouse cells:
FSC-A/SSC-A; FSC-A/FSC-H; FSC-A/live cell marker negative; FSC-A/CD45+; CD3+/B220- or CD19-; CD4/CD8; CD44/CD62L

☒ Tick this box to confirm that a figure exemplifying the gating strategy is provided in the Supplementary Information.
