# Supplementary material for: Lymph node contraction links sex-biased naive CD8+ T cell decline to compromised antigen recognition during middle age
Source: Nat Aging. 2026 Oct 9;6(10):2126–42. doi: 10.1038/s43587-026-01238-4 (PMC13661286; doi:10.1038/s43587-026-01238-4)
Supplement: Supplementary file 3 [file 43587_2026_1238_MOESM3_ESM.pdf]

nature portfolio

# Peer Review File

# Lymph node contraction links sex-biased naive CD8 T cell decline to compromised antigen recognition during middle age

Corresponding Author: Dr Lutz Menzel

**This file contains all reviewer reports in order by version, followed by all author rebuttals in order by version.**

Version 0:

Reviewer comments:

Reviewer #1

(Remarks to the Author)
In this study, Lutz et al present a study investigating the sex-biased decline of naïve CD8+ T cells driven by accelerated, antigen-agnostic differentiation into virtual memory cells. This mechanism is proposed to underlie the age- and sex-associated differential reduction in naïve CD8+ T cell numbers. Through the use of SPx, Lta KO, IL-15Cx, and androgen ablation models, the authors elucidate a naïve T decline and Tvm formation within lymph nodes that compromises anti-tumor immunity in middle-aged male mice and human. While the study is novel and the data presented are generally solid in support of the authors' claims, I have concerns regarding the overstatement of certain conclusions, which require additional experimental support. Additionally, mechanistically study is lacking about how androgen or IL-15Cx regulated the sex-biased Tvm generation and naïve CD8T decline which should be explored.

Major comments:
1 The authors demonstrate that IL-15 and androgen signaling contribute to the decline of naïve CD8+ T cells and the formation of virtual memory T cells. However, direct evidence demonstrating how IL-15 and androgen mechanistically drive Tvm formation is lacking. Notably, IL-15 protein levels are lower in young males and show no difference between middle-aged females and males, a finding that appears inconsistent with the observed changes in naïve CD8+ T cell populations across different ages and sexes.

2 Strong homeostatic TCR signals induce formation of self-tolerant virtual memory CD8 T cells. It remains to be determined whether IL-15 or androgens drive the reduction in TCR clonal diversity observed in male CD8+ T cells. Furthermore, does TCR clonal affinity contribute to lymph node contraction?

3 It remains unclear whether the observed sex-biased lymph node contraction is driven by a decline in CD8+ T cells or by structural damage to the lymph node itself.

4 While the authors convincingly demonstrate the involvement of IL-15, androgens, and antigen-agnostic differentiation in Tvm generation from CD5hi clones, the upstream mechanisms controlling IL-15 production itself remain unexplored. Determining how sex and age influence IL-15 levels would significantly strengthen the study's conclusions.

5 The authors attribute the age-related increase in Tvm cells to a cumulative divergence mechanism. However, this interpretation is not convincing given the modest initial difference in CD5hi naïve CD8+ T cell frequency between young females and males (~0.7% vs. 1.1%). To substantiate this claim, the authors should provide longitudinal data or genetic evidence, such as using conditional CD5 knockout mice.

6 Do female-derived CD5hi naïve CD8+ T cells rapidly convert into virtual memory T cells upon adoptive transfer into male mice?

7 Does middle aged male TVM cells have a unique proliferative and metabolic phenotype which contribute to the sex-biased LN contraction and CD8T ageing.

Minor comments:
1 Extended Data Fig. 1B and Extended Data .6F was not called out in text.

2 The authors should ensure that any tumor cell line is properly designated and described upon its first use in an experiment. Additionally, a more comprehensive and transparent description of the materials and methods employed throughout the study is required.

3 There are two concerns regarding Extended Figure 3e. First, the tetramer staining for OVA-specific CD8+ T cells is of poor quality, making it difficult to identify positive cells. The authors should optimize the staining protocol or consider using a different antibody. Second, there is an inconsistency between the reported frequencies and absolute cell numbers. While Pmel-1+ cells constitute approximately 5% of tumor-draining lymph node (tdLN) CD8+ T cells compared to only 0.6-0.9% for OVA+, the average absolute cell numbers for these two populations are reported as similar. The authors should re-examine the raw data to validate this discrepancy.

4 n Figure 3B, there is a discrepancy between the presented data and the statistical analysis. The representative plot on the left shows nearly identical percentages of CD122+CD44+ Tvm cells in middle-aged females (14%) and males (13%), yet the statistical graph on the right indicates a significant difference between the two groups.

Reviewer #2

(Remarks to the Author)
Menzel et al present an interesting manuscript in which the experiments are well performed and the results important for our understanding of ageing, sex differences in immunity, and when interventions to promote immune health can be effective. The involution of the thymus is one of the best described age-associated immune traits, and yet multiple groups have shown that in advanced aged thymus rejuvenation is insufficient to enhance immune responses. In this manuscript, Padera and colleagues clearly show that impaired thymus function in mid-life males contributes to poor immunity, and that this can be corrected by limiting androgen availability. The mechanistic work is done in mice, with nice human correlate data included to demonstrate that these findings are likely relevant for human health. Overall, this study brings an important new finding to our understanding of thymus involution, and when targeting this can be beneficial for health. There are some minor changes that should be considered prior to publication.

-In the title “aging” should be replaced with “mid-life” (or similar) to better represent the conclusions
-In the abstract consider replacing “tumor recognition” with “response to checkpoint blockade” (or similar) as this is the most compelling pre-clinical data.
-Include the age of the mice in months in the first paragraph of the results when describing “middle-aged” to make it easier to determine the age of the animals used. Including this information in the figure legends will improve readability.
-The extrapolation of mouse results to human lymph nodes leading to the conclusion “which indicates that, despite confounding factors, imaging based LN volume measurements in humans reflect the underlying cellularity. The structure and composition of mouse and humans lymph nodes are different.
-Clarify why an anti-CD40 antibody was used during PR8 immunisation, as this is an unusual immunisation strategy.
-Figure 2b, the data are not continuous for age, but are in discrete groups which means linear regression is not the ideal way to analyse them statisitcally.
-In graphs with multiple comparisons has multiple testing correction been performed? The figure legend implies not.
-In 6h, Interquartile Ranges (25th–75th percentiles) or 95% Confidence Intervals of the median, rather than SEM is a more appropriate way to represent the variance of this data type (assuming non-normal distribution because MWU is the test used).

Reviewer #3

(Remarks to the Author)
A. Summary of the key results:
This study explores sex- and age-dependent changes in naïve $CD8^+$ T cell dynamics and their impact on anti-tumor immunity. The authors show that male mice experience an earlier and more pronounced decline in naïve $CD8^+$ T cells compared to females, accompanied by lymph node contraction and reduced antigen recognition capacity. Mechanistically, this phenotype is associated with both reduced thymic output and increased differentiation into virtual memory T (Tvm) cells. Importantly, androgen blockade partially restores thymic function, expands the naïve $CD8^+$ T cell pool, and improves tumor control in middle-aged males. These findings provide a coherent framework linking immune aging, sex differences, and tumor immunity.

B. Originality and significance:
The manuscript addresses a timely and important question regarding sex differences in immune aging and their relevance to cancer immunity. The integration of thymic involution, Tvm differentiation, and lymph node remodeling is conceptually appealing.
While individual components of the model have been described previously, the study provides a valuable integrative perspective, particularly in highlighting sex-biased timing and functional consequences for tumor immunity. The work,

therefore, represents a meaningful extension of existing knowledge with potential translational implications.

C. Data & methodology:
The study employs a broad range of experimental approaches, including murine tumor models, flow cytometry, TCR analyses, and reanalysis of human datasets. The overall design is comprehensive, and the data are generally of good quality.
The main area where the manuscript could be strengthened is in clarifying mechanistic relationships:
1.Role of naïve $CD8^+$ T cells in tumor immunity:
The data support a strong association between reduced naïve $CD8^+$ T cell numbers and impaired tumor control. It would be helpful to further clarify to what extent this reduction is directly limiting for anti-tumor responses versus reflecting broader immune remodeling.
2.Relationship between thymic output and Tvm differentiation:
The proposed model combining reduced thymic output and increased Tvm differentiation is compelling. Additional clarification—either experimentally or through discussion—of how these processes are linked would improve the conceptual clarity.
3.Tvm functional properties:
The study demonstrates system-level consequences of Tvm expansion. Providing additional insight into the functional characteristics of Tvm cells (e.g., effector potential or contribution to immune regulation) would further strengthen the mechanistic interpretation.
4.Human data interpretation:
The human analyses are supportive and increase translational relevance. A clearer discussion of their limitations (e.g., cross-sectional nature, potential confounders) would improve interpretability.

D. Appropriate use of statistics and treatment of uncertainties
The statistical analyses appear appropriate overall. To further strengthen the rigor, the authors may consider:
1)Providing more emphasis on effect sizes alongside statistical significance
2)Clarifying how multiple comparisons were handled where applicable.
3)Expanding discussion of variability in human datasets
These points are relatively minor but would enhance transparency.

E. Conclusions: robustness, validity, reliability
The conclusions are generally supported by the data and are biologically plausible. Some aspects of the interpretation—particularly regarding mechanistic causality and translational implications—could be presented with slightly more caution to better reflect the current level of evidence.

F. Suggested improvements
The manuscript is already strong, and the following suggestions are intended to further enhance clarity and impact:
1.Further support for the role of naïve $CD8^+$ T cells:
Additional evidence (experimental or discussion-based) clarifying their contribution to tumor immunity would strengthen the central message.
2.Expanded characterization of Tvm cells:
Additional functional insights (if available) or discussion of their potential roles would improve mechanistic depth.
3.Clarification of the proposed model:
A more explicit conceptual model describing the interplay between thymic involution, Tvm differentiation, and lymph node changes would improve readability.
4.Contextualization of human findings:
Briefly outlining limitations and potential confounders would strengthen translational interpretation.
5.Broader validation:
Inclusion of an additional tumor model would be helpful, but is not essential.

G. References: appropriate credit to previous work
The manuscript cites relevant literature. It would be beneficial to more explicitly position the current findings within the broader context of:
1)Immune aging and thymic involution
2)Virtual memory T cell biology
3)Sex differences in immune responses
to better highlight the study's contribution.

H. Clarity and context
The manuscript is generally well written and clearly structured. The central message is understandable.
Minor improvements that could further enhance clarity include:
1)Presenting the mechanistic framework more explicitly.
2)Simplifying some dense sections in the Results.
3)Slightly tempering speculative statements in the Discussion.

Additional suggestion on presentation clarity
To further enhance the clarity of the proposed mechanistic framework, the authors may consider including a schematic diagram or workflow-style figure summarizing the overall experimental design and key biological relationships.

In particular, a visual representation integrating thymic involution, naïve $CD8^+$ T cell decline, Tvm differentiation, lymph node contraction, and their collective impact on tumor immunity would greatly improve readability and help readers better follow the central model of the study.
Such a schematic would also be useful in clearly distinguishing experimentally supported relationships from more conceptual or inferred connections.

Overall assessment:
This is a well-designed and insightful study that provides an integrated view of sex-biased immune aging and its impact on tumor immunity. The manuscript would benefit from modest improvements in mechanistic clarification and contextual framing, but the core findings are compelling.

Recommendation: Major Revision

Version 1:

Reviewer comments:

Reviewer #1

(Remarks to the Author)
The authors have fully addressed my comments, and the revised manuscript demonstrates notable improvements.

Reviewer #2

(Remarks to the Author)
The authors have addressed the points raised in the first round of review.

**Open Access** This Peer Review File is licensed under a Creative Commons Attribution 4.0 International License, which permits use, sharing, adaptation, distribution and reproduction in any medium or format, as long as you give appropriate credit to the original author(s) and the source, provide a link to the Creative Commons license, and indicate if changes were made.
In cases where reviewers are anonymous, credit should be given to 'Anonymous Referee' and the source.
The images or other third party material in this Peer Review File are included in the article's Creative Commons license, unless indicated otherwise in a credit line to the material. If material is not included in the article's Creative Commons license and your intended use is not permitted by statutory regulation or exceeds the permitted use, you will need to obtain permission directly from the copyright holder.
To view a copy of this license, visit https://creativecommons.org/licenses/by/4.0/

## Rebuttal letter

**Reviewer #1 (Remarks to the Author):**

**In this study, Lutz et al present a study investigating the sex-biased decline of naive CD8+ T cells driven by accelerated, antigen-agnostic differentiation into virtual memory cells. This mechanism is proposed to underlie the age- and sex-associated differential reduction in naive CD8+ T cell numbers. Through the use of SPx, Lta KO, IL-15Cx, and androgen ablation models, the authors elucidate a naive T decline and Tvm formation within lymph nodes that compromises anti-tumor immunity in middle-aged male mice and human. While the study is novel and the data presented are generally solid in support of the authors' claims, I have concerns regarding the overstatement of certain conclusions, which require additional experimental support. Additionally, mechanistically study is lacking about how androgen or IL-15Cx regulated the sex-biased Tvm generation and naive CD8T decline which should be explored.**

**Major comments:**

**1 The authors demonstrate that IL-15 and androgen signaling contribute to the decline of naive CD8+ T cells and the formation of virtual memory T cells. However, direct evidence demonstrating how IL-15 and androgen mechanistically drive Tvm formation is lacking. Notably, IL-15 protein levels are lower in young males and show no difference between middle-aged females and males, a finding that appears inconsistent with the observed changes in naive CD8+ T cell populations across different ages and sexes.**

We thank the reviewer for highlighting this important point. We agree that our data do not establish a direct mechanistic role for IL-15 or androgen signaling as primary drivers of virtual memory (Tvm) differentiation. Our findings support a model in which these factors modulate the susceptibility of the naive CD8 T cell pool to antigen-agnostic differentiation.

We wish to clarify a potential source of confusion in our original manuscript. IL-15 is required as a permissive baseline signal for Tvm maintenance and expansion, but is not itself the driver of sex- or age-dependent differences in naive CD8 T cell decline via Tvm formation (Sosinowski et al., 2013; White et al., 2017, 2016). In our study, the IL-15/IL-15Rα complex was used as an experimental tool to uniformly lower the activation threshold for Tvm differentiation across a physiologically diverse, endogenous TCR repertoire, thereby demonstrating that preferential conversion of more self-reactive clones into Tvms is sufficient to drive naive CD8 T cell attrition. This experimental perturbation was not intended to model the endogenous mechanism underlying sex differences, but as proof of concept that enhanced Tvm conversion early in life induces cumulative subset shifts that become manifest in the naive CD8 T cell pool over time.

The observation that steady-state IL-15 protein levels are lower in young males but do not differ between sexes in middle age initially appears inconsistent with the observed sex-biased naive CD8 T cell decline. However, IL-15 mRNA levels do not differ between sexes at either age, suggesting that the differences in protein abundance are unlikely to reflect transcriptional regulation. Rather than differential production, we hypothesize that the transient reduction in IL-

15 protein in young males may instead reflect increased local consumption by an expanding Tvm population. To avoid overstating the significance of this finding, we have moved the IL-15 protein data to the Extended Data figures, where it is presented alongside the mRNA data for direct comparison **(Extended Data Fig. 9f,g)**. We have also added a brief conclusion to describe these data.

To further clarify the conceptual framework, we have included a new schematic illustration that separates the mechanistic branches of the model, highlighting the distinct roles of thymic output, IL-15-mediated permissive signaling, and TCR self-reactivity in shaping naive CD8 T cell declines and Tvm formation (**Rebuttal letter Fig. 1** and **Fig.6i of the revised manuscript)**.

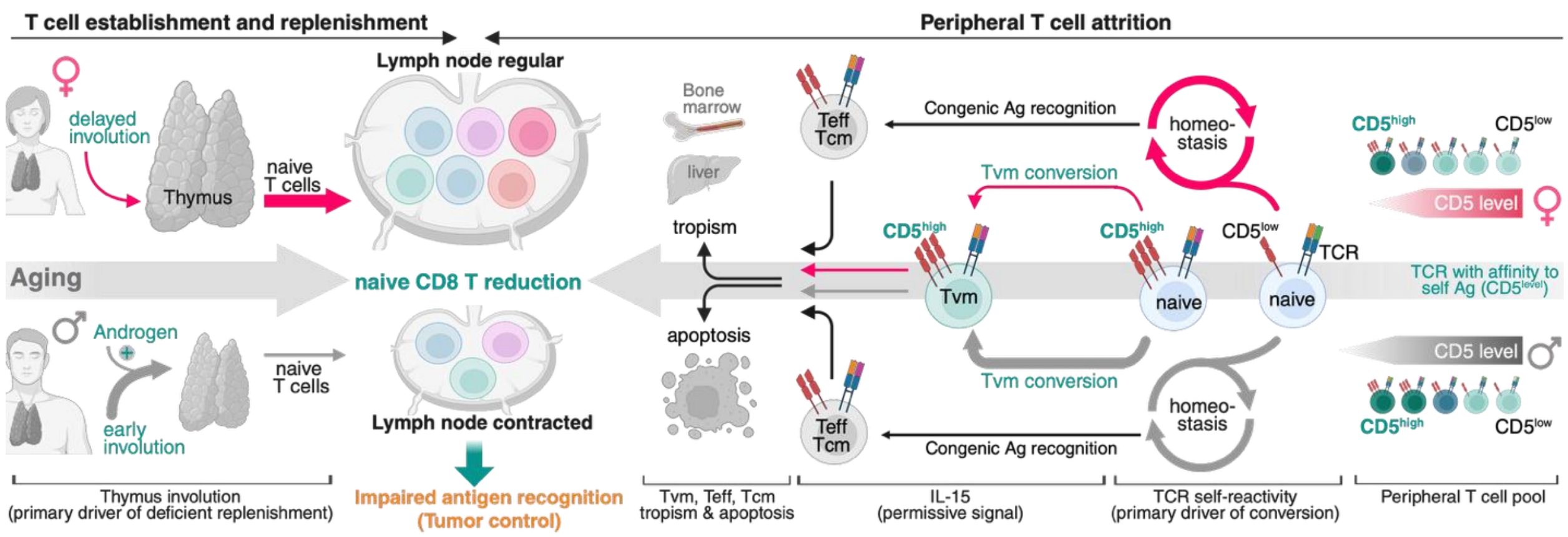


**Rebuttal letter Fig. 1. and Fig. 6i of the revised manuscript.** Conceptual model linking sex-biased thymic involution and TCR self-reactivity–driven Tvm conversion to naive CD8⁺ T cell loss, LN contraction, and impaired antigen recognition.

**2 Strong homeostatic TCR signals induce formation of self-tolerant virtual memory CD8 T cells. It remains to be determined whether IL-15 or androgens drive the reduction in TCR clonal diversity observed in male CD8+ T cells.**

We agree that IL-15 and androgen signaling should not be interpreted as primary drivers of naive T cell to Tvm conversion. Rather, our data and other studies (Sosinowski et al., 2013; White et al., 2017, 2016) support a model in which intrinsic TCR self-reactivity (indicated by CD5) represents the dominant determinant of susceptibility to antigen-agnostic differentiation.

**Furthermore, does TCR clonal affinity contribute to lymph node contraction?**

Our OT-I experiments directly address these points raised by the reviewer. First, OT-I T cells are well established to display high TCR self-reactivity compared to the polyclonal repertoire in wild type mice (Drobek et al., 2018; White et al., 2017), as evidenced by the elevated CD5 expression of OT-I cells. The CD5$^{high}$ phenotype makes OT-I cells well-suited to experimentally amplify the TCR self-reactivity axis in an otherwise physiological setting. Skewing the TCR repertoire toward higher self-reactivity was sufficient to drive accelerated Tvm formation and naive CD8 T cell loss **(Fig. 3d of the revised manuscript)**.

Moreover, directly relevant to the question of whether TCR self-affinity contributes to LN contraction, OT-I mice exhibit a strong lymph node contraction between young-adults and middle-aged **(Rebuttal letter Fig. 2, Fig. 3f of the revised manuscript)**. Notably, these effects are largely comparable between females and males in the OT-I setting, with only modest residual differences, indicating that uniform TCR affinity in both sexes substantially reduces the sex-biased divergence observed in the polyclonal repertoire

Regarding the IL-15 complex experiments, as detailed in our response to comment 1, IL-15 perturbation does not alter TCR specificity but lowers the activation threshold for Tvm differentiation within a physiologically diverse endogenous TCR repertoire, further supporting the view that IL-15 acts as a permissive signal rather than a primary driver.

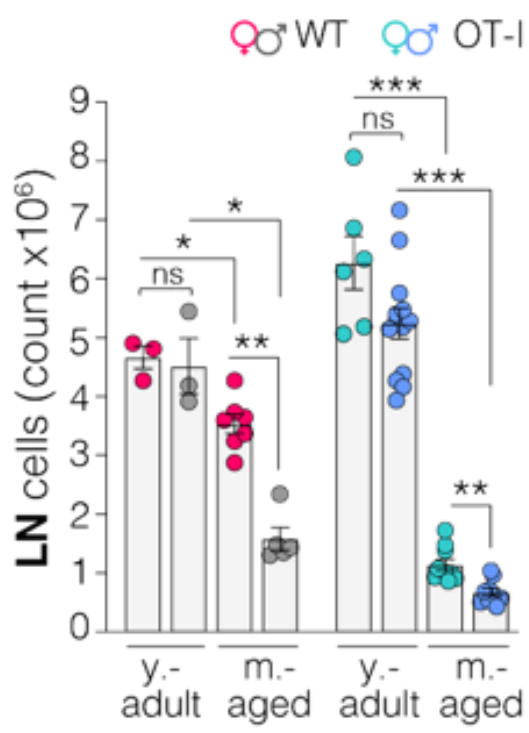


**Rebuttal letter Fig. 2. and Fig. 3f of the revised manuscript.** Quantification of total cell count of inguinal LNs of young adults (y.-adults) and middle-aged (m.-aged) WT and OT-I mice. Statistics calculated with MWU test, ns=nonsignificant, *p<0.05, **p<0.01, ***p<0.001.

**3 It remains unclear whether the observed sex-biased lymph node contraction is driven by a decline in CD8+ T cells or by structural damage to the lymph node itself.**

We thank the reviewer for this important point. To comprehensively address whether LN contraction in middle-aged males reflects structural pathology rather than lymphocyte-driven attrition, we have substantially expanded our histological analysis of LN architecture **(Extended Data Fig. 6a)**.

The compartmental architecture of LNs, including discrete T cell zones, B cell follicles, and medullary regions, is well preserved in middle-aged mice of both sexes, with no evidence of structural disorganization **(Extended Data Fig. 6a, top left)**. To evaluate the integrity of naive T cell entry sites and intranodal migration cues, we quantified high endothelial venules (HEVs) using PNAd staining and assessed the CCL21 chemokine gradient within the T cell zone. HEV abundance and CCL21 expression were comparable between middle-aged females and males **(Extended Data Fig. 6a, top middle and right)**.

Third, to characterize the fibroblastic reticular cell (FRC) network, we stained for podoplanin (Pdpn), collagen IV (Col IV), and desmin (Des), alongside collagen I (Col I) and alpha-smooth muscle actin (aSMA) as markers of broader stromal remodeling. None of these markers differed significantly between sexes, and the FRC network appeared structurally intact in both females and males, with preserved interfollicular and T cell zone FRC network structures. This is

consistent with published data showing that FRC numbers and T cell zone architecture are largely preserved at steady state in mice of comparable age (Masters et al., 2019; Turner and Mabbott, 2017). We also quantified blood (CD31) and lymphatic (Lyve1) endothelial cells, as well as the abundance of dendritic cells (CD11c), and found no significant differences between sexes or age groups **(Extended Data Fig. 6a)**. Together, these data provide evidence that the stromal and vascular infrastructure of LNs is intact and equivalent between middle-aged females and males, and does not account for the observed sex-biased LN contraction.

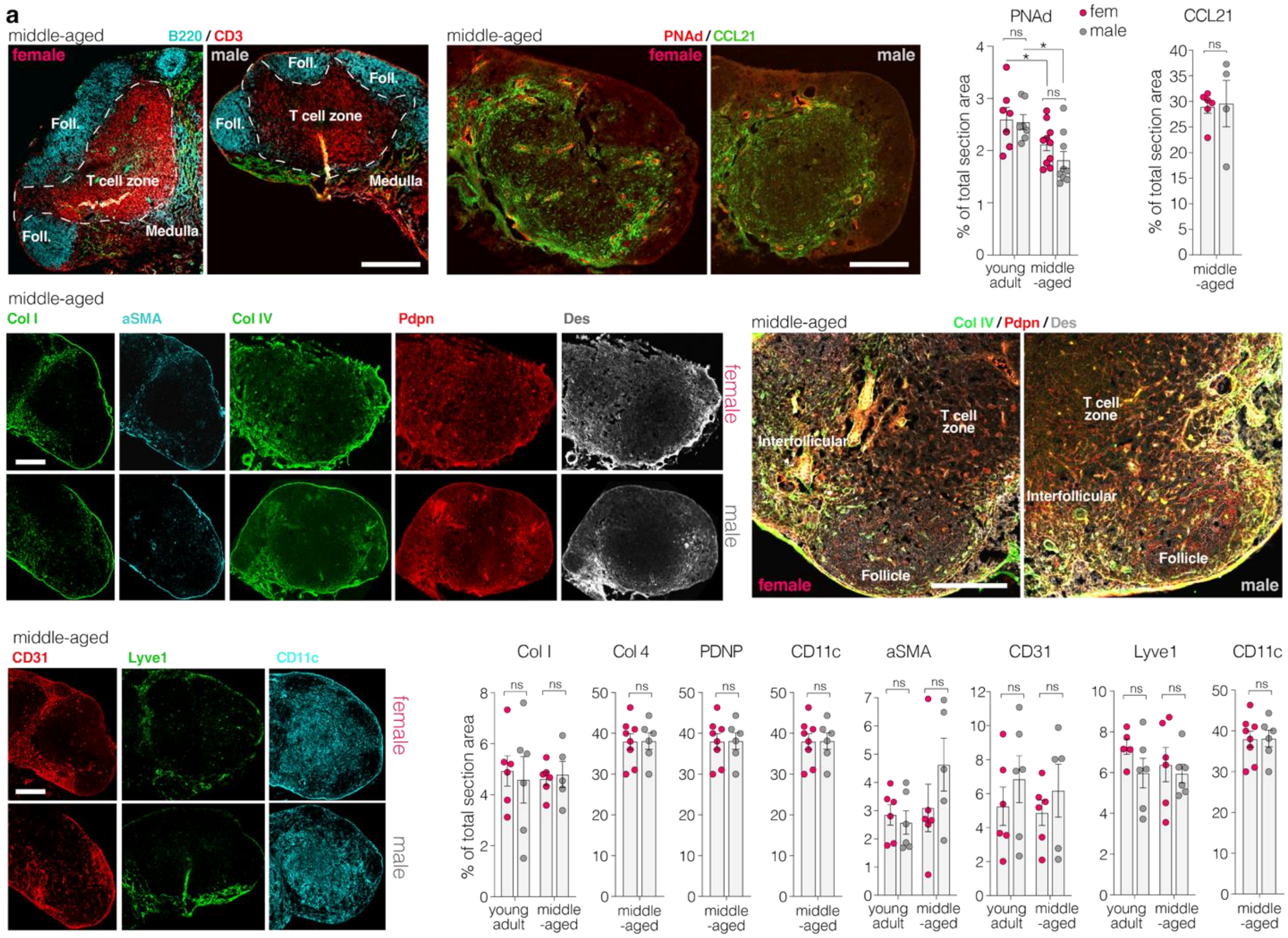


**Rebuttal letter Fig. 3. and Extended Data Fig. 6a of the revised manuscript. Inguinal LN architecture in young-adults and middle-aged mice. a,** Representative images and quantification of inguinal LN sections; B220 (B cells), CD3 (T cells), peripheral node adressin (PNAd, high endothelial venules), C-C motif chemokine ligand 21 (CCL21), collagen I (Col I), alpha smooth muscle actin (αSMA), Col IV, podoplanin (Pdpn), desmin (Des), CD31 (PECAM-1, endothelial cells), lymphatic vessel endothelial hyaluronan receptor-1 (Lyve-1, lymphatic endothelial cells), CD11c (dendritic cells). Scale bar: 200 µm and 50 µm (FRC network images). Data shown as mean ± SEM with data points representing single LNs of individual animals. Statistics calculated with MWU test, ns=nonsignificant, *$p<0.05$.

Besides the direct histological assessment, some of our experimental models directly perturb T cell populations without targeting stromal compartments, which consistently result in LN contraction. These include antibody-mediated T cell depletion, irradiation-induced lymphodepletion **(Extended Data Fig. 6e)**, the OT-I model with skewed TCR self-reactivity **(Fig. 3f)**, and IL-15/IL-15Rα complex-induced Tvm formation and naive T cell loss **(Fig. 4g)** and shown in **Rebuttal letter Fig. 4a-d**.

We would note that thymic rejuvenation strategies, including androgen ablation, effectively restore naive T cell numbers and improve immune function in young adult and middle-aged mice, but show diminishing efficacy in truly old/aged animals, which is when LN fibrosis and structural

deterioration become more prominent (Thompson et al., 2019) **(Rebuttal letter Fig. 4a,b and Fig. 6 of the revised manuscript)**. The fact that androgen ablation is effective in our middle-aged model argues against significant structural lymph node pathology at this stage. We have revised the discussion to clarify that while lymphocyte-driven contraction is the dominant early mechanism, progressive stromal alterations are likely to become increasingly relevant at more advanced ages. Together, these findings support a lymphocyte-based mechanism underlying LN contraction.

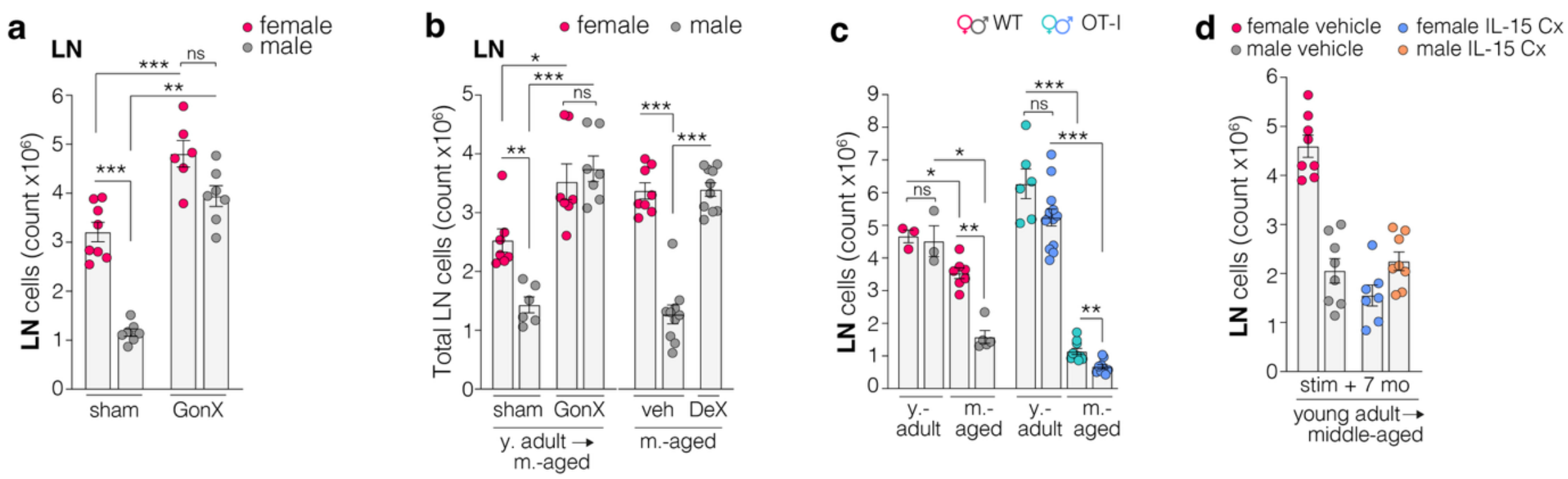


**Rebuttal letter Fig. 4.** a, Quantification of the total cell counts of ingLNs of mice with sham surgery or gonadectomy (GonX) in middle-aged with analysis two month after surgery, Extended Data Fig. 13h of the revised manuscript. b, Quantification of the total cell counts of ingLNs of mice with, left, sham surgery or gonadectomy (GonX) in young-adults (y.-adult) with analysis when mice are middle-aged, right, middle-aged mice were treated with vehicle (veh) or Degarelix (DeX)Extended Data Fig. 13h of the revised manuscript, c, Quantification of the total cell counts of ingLNs of WT and OT-I mice, young adults (y. adult) and middle-aged (m.-aged). Now included in Fig. 3b of the revised manuscript. d, Quantification of the total cell counts of ingLNs of mice treated with vehicle or IL-15 Cx; middle-aged (m.-aged). Now included in Extended Data Fig. 9h of the revised manuscript. Data shown as mean ± SEM with data points representing single LNs of individual animals. Statistics calculated with MWU test (a,b,c,d); ns=nonsignificant, *p<0.05, **p<0.01, ***p<0.001.

**4 While the authors convincingly demonstrate the involvement of IL-15, androgens, and antigen-agnostic differentiation in Tvm generation from CD5hi clones, the upstream mechanisms controlling IL-15 production itself remain unexplored. Determining how sex and age influence IL-15 levels would significantly strengthen the study's conclusions.**

We thank the reviewer for this comment. As detailed in our responses to comments 1 and 2, our model does not invoke IL-15 production as the upstream determinant of sex- or age-dependent differences in naive CD8 T cell decline. Rather, IL-15 serves as a permissive baseline signal required for Tvm conversion and maintenance, while the sex-biased divergence in early Tvm conversion has origins in increased TCR self-reactivity-driven peripheral differentiation in males. The observation that IL-15 mRNA levels do not differ between sexes, while protein levels show only transient and modest differences (**Extended Data Fig. 9f,g)**, further argues against differential IL-15 production as a meaningful upstream regulator of the observed increased Tvm formation in young males.

We agree that understanding what regulates IL-15 production across sex and age more broadly is an interesting question, but addressing it mechanistically would require a separate body of work that goes beyond the scope of the current study, especially since we do not see sex-related differences on the transcriptional level. We have clarified this point in the revised manuscript to

ensure the conceptual framework is presented with sufficient precision to avoid the confusion created by the initial manuscript.

**5 The authors attribute the age-related increase in Tvm cells to a cumulative divergence mechanism. However, this interpretation is not convincing given the modest initial difference in CD5hi naive CD8+ T cell frequency between young females and males (~0.7% vs. 1.1%). To substantiate this claim, the authors should provide longitudinal data or genetic evidence, such as using conditional CD5 knockout mice.**

We agree that the modest initial difference in CD5hi naive CD8 T cell frequency between young females and males (~0.7% vs. 1.1%) requires careful interpretation, and we appreciate the opportunity to clarify the evidence supporting the cumulative divergence model.

Genetic evidence for our model is provided by TCR transgenic systems (OT-I, gBT, F5), in which the T cell compartment is globally shifted toward lower or higher self-reactivity (Drobek et al., 2018; Haluszczak et al., 2009; White et al., 2016). In these models, increased TCR self-reactivity is sufficient to drive accelerated Tvm formation and loss of naive CD8 T cells (**Fig. 3b,c,d,e**). These data directly demonstrate that the magnitude of TCR self-reactivity strongly influences the rate of naive T cell attrition, as we demonstrate in our study**.**

A conditional CD5 knockout would not directly test our model, as CD5 serves as a proxy for TCR self-reactivity rather than a causal driver of Tvm differentiation. Removing CD5 would therefore eliminate the readout without altering the underlying parameter of interest, namely TCR signaling strength.

Second, while the initial frequency difference is modest, our data across age groups shows that this difference compounds progressively over time, with Tvm frequencies diverging substantially between sexes from young adult to middle-aged to aged animals (Fig. 3b). This trajectory is consistent with a cumulative process and not with a model in which the sex difference arises acutely at a single time point. Using models of compounding, we have computationally shown that even beginning with a difference in conversion of 0.7% vs. 1.1% significant decreases in naïve pools can occur over time (**Supplementary Data Fig. 1; Rebuttal Letter Figure 5**). Importantly, the ~0.7% vs. 1.1% difference refers to a subset within a compartment that constitutes over 85% of all CD8 T cells in young adults. Thus, even small relative shifts correspond to substantial differences in the absolute number of self-reactive clones available for Tvm conversion. As illustrated in **Fig. 3j**, small differences in conversion rates, when compounded over time, are sufficient to produce the magnitude of divergence observed at middle age. Taken together, the mechanistic evidence from TCR transgenic models, the progressive divergence observed across age groups, and the concordant human data support a cumulative divergence model as consistent with our findings.

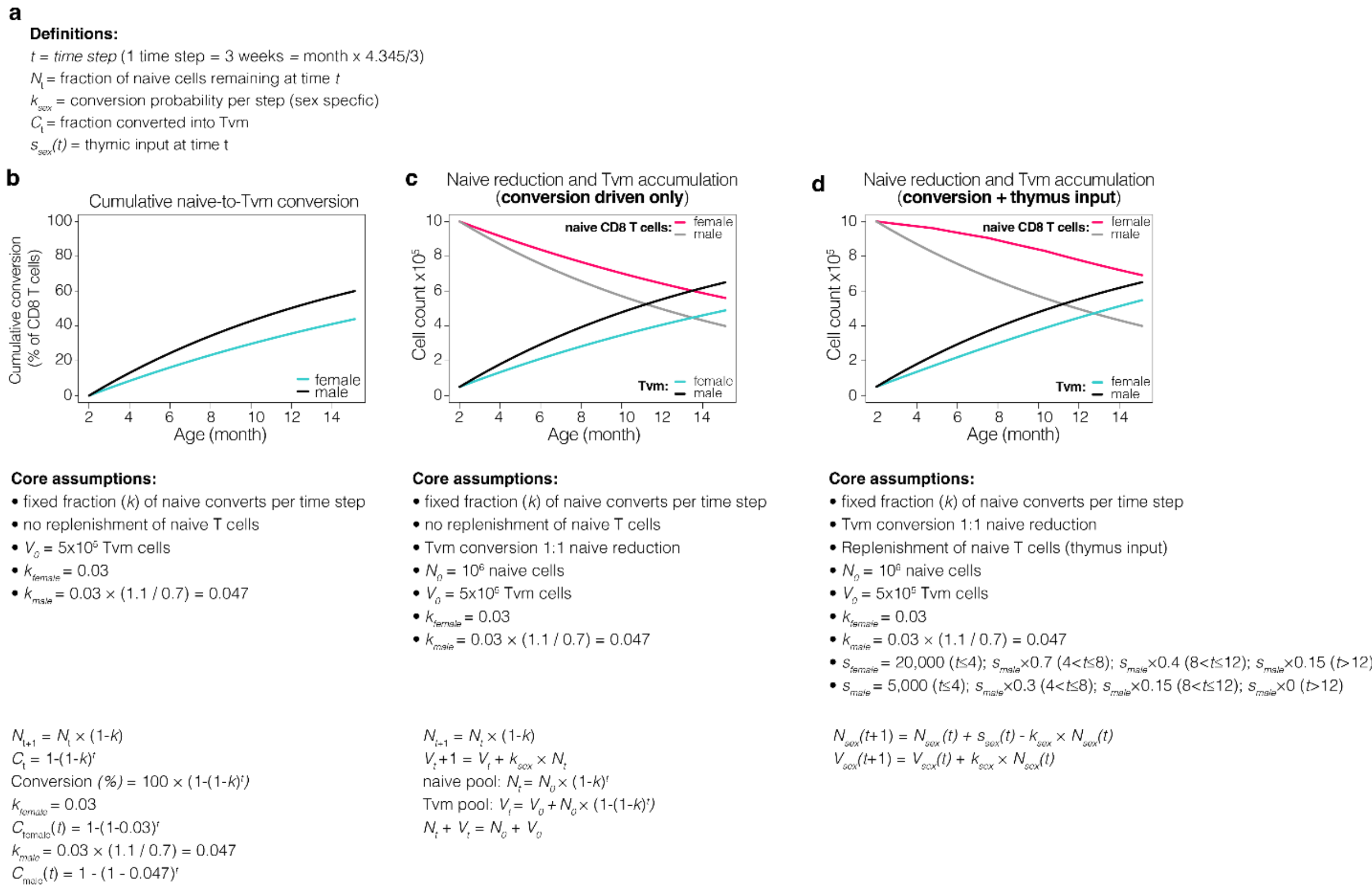


**Rebuttal letter Fig. 5 and Supplementary Data Fig. 1 of the revised manuscript. Conceptual framework for cumulative naive CD8 T cell to virtual memory T cell conversion as an illustrative example. a,** Definition of parameters. **b, *Cumulative conversion over time.*** To assess whether the observed sex differences in conversion propensity are sufficient to account for progressive divergence in naive CD8 T cell maintenance, we implemented a minimal discrete-time model of cumulative naive-to-Tvm conversion based on models of compounding. In this framework, a fixed fraction *k* of the remaining naive pool converts to Tvm cells per time interval, such that the naive fraction evolves according to $N_{t+1} = N_t(1 - k)$, yielding $N_t = (1 - k)^t$. The cumulative fraction of converted cells is therefore $C_t = 1 - (1 - k)^t$. Based on published adoptive transfer experiments (White et al., *Nature Communications* 2016), we set $k_{female} = 0.03$ per 3-week interval for females. To account for the increased frequency of conversion-prone cells in males, we scaled this rate by the observed ratio of $CD5^{high}$ naive CD8 T cells **(Fig. 3f)**, yielding $k_{male}$ 0.03*(1.1/0.7) = 0.047. This minimal model demonstrates that modest differences in per-interval conversion rates (0.03 vs. 0.047) produce substantial divergence in cumulative conversion over time. **c*, Naive reduction and Tvm accumulation (conversion driven only).*** We modeled naive CD8 T cell decline as a discrete-time process in which a fixed fraction *k* of the naive pool converts to Tvm cells per interval, in the absence of thymic input. This yields $N_{t+1} = N_t(1 - k)$ and $V_{t+1} = V_t + k*N_t$, resulting in exponential decay of the naive pool and corresponding cumulative accumulation of Tvm cells. **d*, Naive reduction and Tvm accumulation (conversion + Thymic input).*** To incorporate sex-biased thymic support, we extended the conversion model by adding a time-dependent thymic input term to the naive compartment. Naive CD8 T cell dynamics were modeled as $N_{t+1} = N_t + s_t - k*N_t$, with corresponding Tvm accumulation given by $V_{t+1} = V_t + k*N_t$. Thymic input was modeled as a gradually declining function *s* with more sustained thymic input in females compared to males.

## 6 Do female-derived CD5hi naive CD8+ T cells rapidly convert into virtual memory T cells upon adoptive transfer into male mice?

We thank the reviewer for this important suggestion and attempted the proposed adoptive transfer experiment. Briefly, lymphocytes from spleens and LNs of young adult CD45.1 donor mice were enriched for CD8 T cells by magnetic separation, followed by fluorescence-activated cell sorting to isolate naive CD8 T cells enriched for either $CD5^{low}$ or $CD5^{high}$ expression levels (**Rebuttal letter Fig. 6a)**. We note that this strategy yields an enrichment of $CD5^{low}$ and $CD5^{high}$ cells rather

than a clean, homogeneous population (**Rebuttal letter Fig. 6a)**. 1x10⁶ sorted cells per fraction were transferred intravenously into untreated congenic (CD45.2) hosts of the other sex, respectively.

Despite recording up to 25 million events per sample during flow cytometry analysis, we recovered insufficient numbers of donor-derived CD45.1 cells for reliable quantitative analysis. This might reflect the competitive disadvantage of transferred naive T cells in a replete, non-lymphopenic host, where endogenous T cells occupy available homeostatic niches (Hataye et al., 2006; Moses et al., 2003). We are transparent that repeating this experiment under the same conditions is unlikely to yield substantially better results, as the low recovery appears to be an inherent limitation of this approach in non-lymphopenic settings rather than a technical issue that could be resolved by protocol optimization.

We nonetheless present the data in the rebuttal figure, as the overall tendency is consistent with our model that donor $CD5^{high}$ naive CD8 T cells showed a greater propensity to acquire a Tvm phenotype ($CD44^{+}CD122^{+}CD49d^{-}$) compared to $CD5^{low}$ cells following transfer, regardless of donor or recipient sex **(Rebuttal letter Fig. 6c)**. This directional finding is consistent with the established role of TCR self-reactivity, reflected by CD5 expression, as the primary determinant of Tvm conversion (Drobek et al., 2018; White et al., 2016), and argues against a dominant role for the recipient hormonal environment in driving this process.

To more robustly address the underlying question of whether Tvm conversion is governed by TCR-intrinsic self-reactivity rather than the sex of the recipient environment, we want to point to our OT-I experiments **(Fig. 3c-f)**, in which skewing the entire CD8 T cell compartment toward high self-reactivity was sufficient to drive equivalent and accelerated Tvm formation in both female and male mice, independent of the sex. This is further supported by White et al. 2016, who directly demonstrated that $CD5^{high}$ naive cells preferentially convert into Tvm cells.

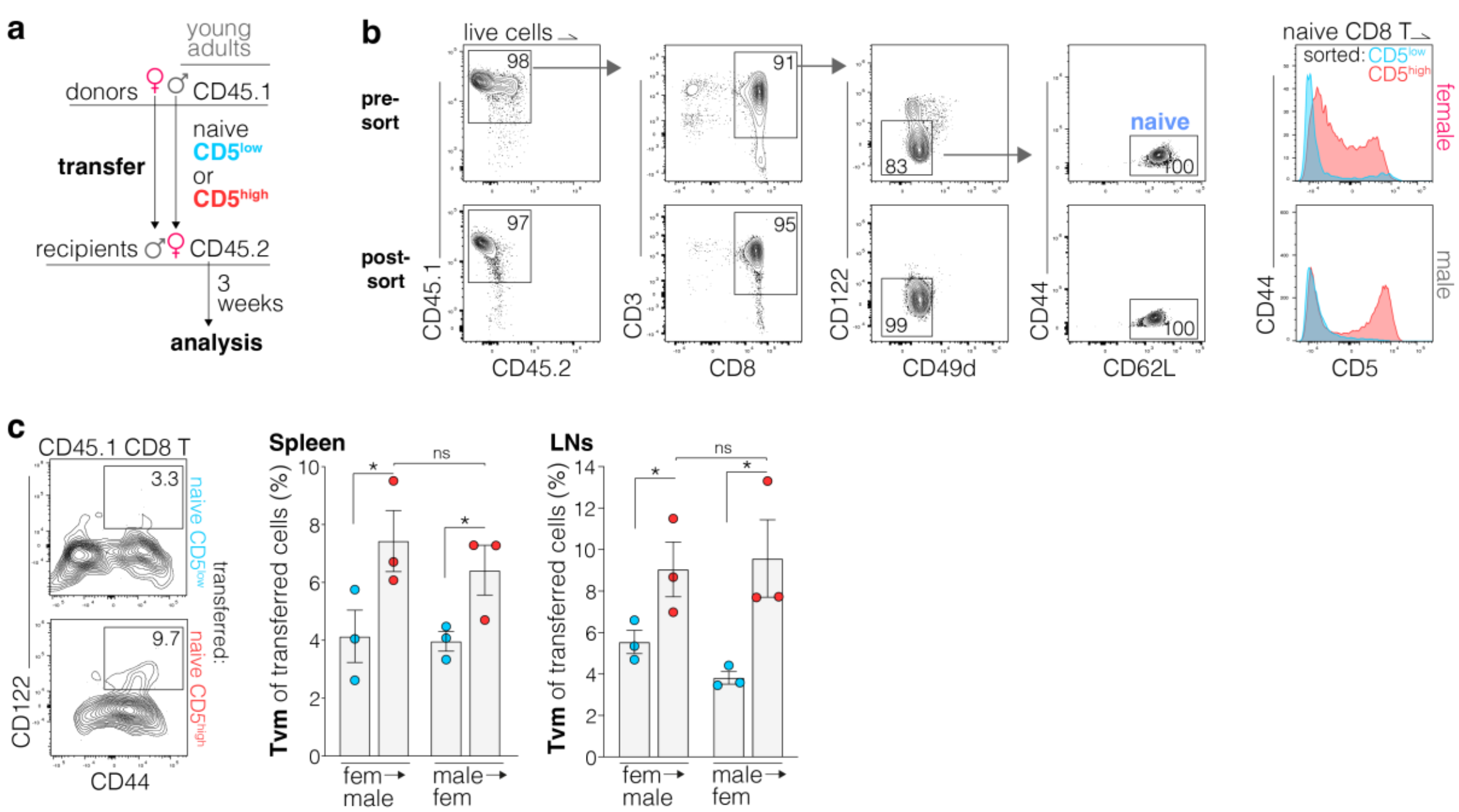


**Rebuttal letter Fig. 6 (for Reviewers only). a,** Adoptive naive T cell transfer from CD45.1 mice to congenic wild type mice of the other sex, both young adults. **b**, Naive $CD5^{high}$ (the top 20% of CD5 expression) or $CD5^{low}$ (the bottom 20% of CD5 expression) CD8 T cells were sorted from untreated CD45.1 mice, and 1x10⁶ of each population were adoptively

transferred into congenic WT recipients of the other sex. **c**, 3 weeks later, percentage of Tvm of donor CD8 T cells were analyzed in lymph nodes and spleen. Data shown as mean ± SEM with data points representing single LNs of individual animals. Statistics calculated with students t test; ns=nonsignificant, *p<0.05.

**7 Does middle aged male TVM cells have a unique proliferative and metabolic phenotype which contribute to the sex-biased LN contraction and CD8T ageing.**

We thank the reviewer for this insightful question. To address this question, we performed complementary proliferation and transcriptomic analyses.

Following 5 days of CD3/CD28 stimulation, middle-aged Tvm cells exhibited a significant age-associated decline in proliferative capacity that was independent of sex, whereas proliferation of naive CD8 T cells was preserved with aging. In contrast, Tvm cells from young adult mice displayed proliferative responses comparable to those of naive cells **(Rebuttal letter Fig. 7a)**.

To further define the underlying cellular state, we performed bulk RNA sequencing of sorted naive and Tvm CD8 T cells from young adult and middle-aged male and female mice, unstimulated or after short-term stimulation **(Rebuttal letter Fig. 7a).** Principal component analysis revealed that the overall transcriptional identity is primarily defined by naive vs Tvm (62% proportion of variance) and stimulation groups (17%). Age influences the distribution of Tvm but not naive cells **(Rebuttal letter Fig. 7c)**. Consistent with the higher apoptotic rate observed in Tvm cells **(Extended Data Fig. 9d)**, gene set scoring across senescence-associated hallmarks revealed that middle-aged Tvm cells upregulate apoptosis, senescence and terminal differentiation signatures compared to naive cells **(Rebuttal letter Fig. 7d)**. To address the reviewer's question regarding metabolic characteristics of Tvm cells, we performed KEGG pathway enrichment analysis across all groups. Metabolic pathway analysis revealed that transcriptional changes were almost exclusively driven by stimulation status rather than age or sex **(Rebuttal letter Fig. 7e).** While stimulation induced broad and significant metabolic reprogramming across all groups, comparisons between young adult and middle-aged mice revealed minimal age-dependent metabolic differences, and no sex-specific metabolic signature was detected in Tvm cells of either age.

Collectively, these data indicate that middle-aged Tvm cells are progressively lost through a combination of impaired proliferative self-renewal and increased senescence-driven cell death, while their functional metabolic state remains predominantly shaped by activation signals rather than intrinsic age- or sex-related reprogramming. Importantly, these transcriptional and functional characteristics are shared between male and female Tvm cells. As demonstrated in our manuscript, the sex-specific difference at least in part originates at the level of naive-to-Tvm conversion, which is determined by TCR self-reactivity as reflected by CD5 expression levels and differs between male and female mice.

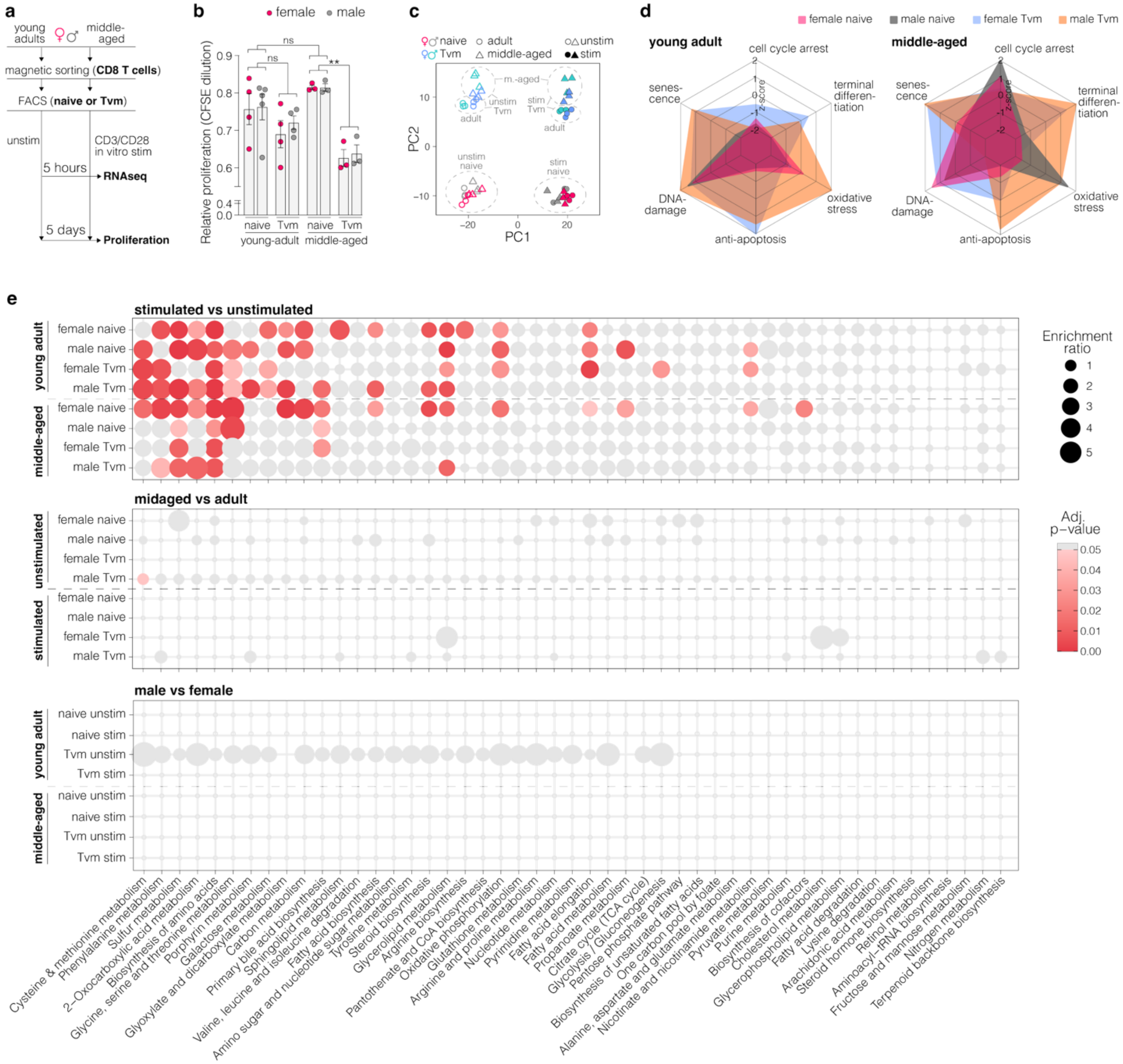


**Rebuttal letter Fig. 7, Fig.4 and Supplementary Data Fig. 2 of the revised manuscript. Transcriptional and functional characterization of TVM and naive CD8+ T cells across age and sex. a,** Experimental setup. Spleen and LN cells were enriched for CD8 T cells by magnetic separation, followed by fluorescence-activated cell sorting to isolate naive CD8 T and Tvm cells. Proliferation (indicated by CFSE dilution) and transcriptomic data (bulk RNA sequencing) were assessed in cells from young adult and middle-aged male and female mice. **b (Fig. 4b)**, Proliferation-associated CFSE mean fluorescence intensity dilution relative to unstimulated control cells after 5 days of CD3/CD28 in vitro stimulation. **c**, Principal component (PC) analysis of VST-normalized bulk RNA-seq counts across all 48 samples. Colors indicate cell type and sex; shapes indicate age. **d (Fig. 4c)**, Radar plots showing z-scored mean expression of senescence-associated gene sets in young adult and middle-aged naive and Tvm CD8 T cells across all groups. Gene sets described in material and methods. **e (Supplementary Data Fig. 2)**, KEGG metabolic pathway enrichment analysis. Dot size indicates enrichment ratio: (differentially expressed genes in pathway / total differentially expressed genes genes) / (all genes in pathway / all genes in genome); color indicates FDR-adjusted p-value (grey = not significant, red = adj. p < 0.05). Data shown as mean ± SEM with data points representing single LNs of individual animals. Statistics calculated with student's t test (b) or BH-corrected hypergeometric test p-value (e); ns=nonsignificant, **p<0.01.

## Minor comments:

**1 Extended Data Fig. 1B and Extended Data .6F was not called out in text.**

We thank the reviewer for pointing this out. **Extended Data Fig. 1b** is referenced in the Introduction (line 72). To improve clarity and ensure direct linkage to the corresponding results, we have now also explicitly cited the figure at the beginning of the Results section (line 95).

Extended Data Fig. 6f presents the flow cytometry gating strategy for murine lymphocytes. We have now explicitly referenced this panel in the Methods section (line 716) and in the corresponding figure legends for **Fig. 2, Fig. 3** and **Fig. 5** (lines 1206, 1222 and 1272).

**2 The authors should ensure that any tumor cell line is properly designated and described upon its first use in an experiment. Additionally, a more comprehensive and transparent description of the materials and methods employed throughout the study is required.**

We thank the reviewer for this comment. We have revised the manuscript to ensure that all tumor cell lines are defined upon first mention in the Results section (line 110, line 113, line 149). In addition, we have expanded the Materials and Methods section to provide a more comprehensive and transparent description of tumor models and cell lines (line 576-580).

**3 There are two concerns regarding Extended Figure 3e. First, the tetramer staining for OVA-specific CD8+ T cells is of poor quality, making it difficult to identify positive cells. The authors should optimize the staining protocol or consider using a different antibody. Second, there is an inconsistency between the reported frequencies and absolute cell numbers. While Pmel-1+ cells constitute approximately 5% of tumor-draining lymph node (tdLN) CD8+ T cells compared to only 0.6-0.9% for OVA+, the average absolute cell numbers for these two populations are reported as similar. The authors should re-examine the raw data to validate this discrepancy.**

We thank the reviewer for this important comment. Regarding the tetramer staining quality, we agree that the previously shown example in **Extended Data Fig. 3e** was not fully representative. We have replaced it with a clearer example that better reflects the percentage of positive cells and the related values to total positive cells per tetramer.

Importantly, tetramer$^{+}$ CD8 T cells in tumor-draining lymph nodes are typically rare, as antigen-specific T cells rapidly egress from the tdLN following activation and traffic to the tumor. This results in low-frequency populations that are inherently more difficult to identify compared to tumor samples. To ensure specificity and exclude technical artifacts, tetramer$^{+}$ populations were stringently gated as shown **(Extended Fig. 3a)**, ensuring that cells were not double-positive across tetramers. In addition, examples of tetramer staining within the primary tumor **(Extended Data Fig. 3b)** demonstrate robust signal separation, confirming that the staining protocol itself performs reliably.

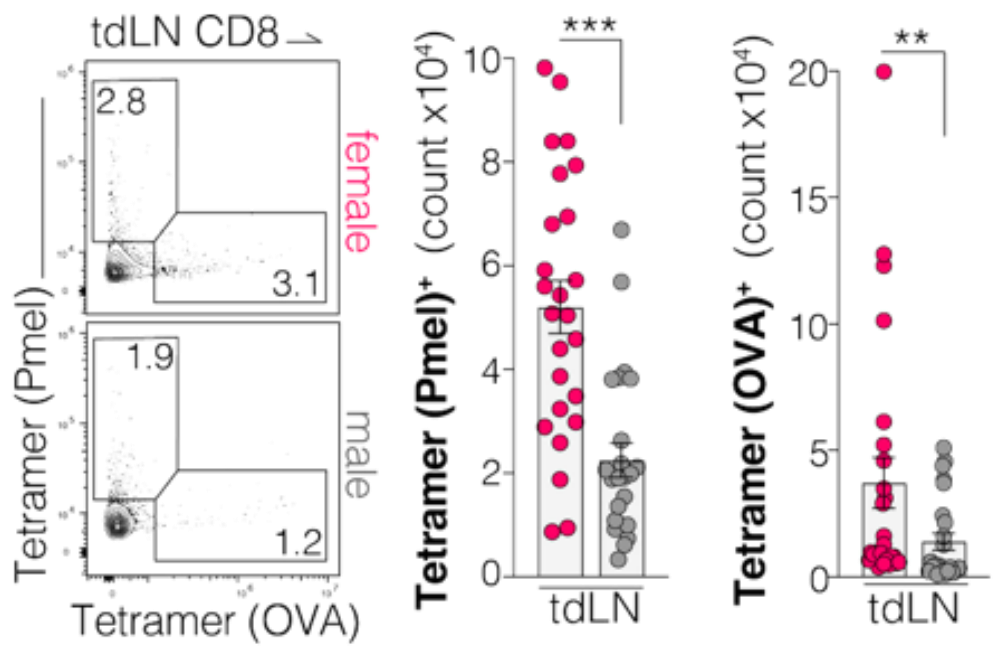


**Rebuttal letter Fig. 8. and Extended Data Fig. 3e of the revised manuscript.** Flow cytometry analysis of tetramer staining of CD8 T cells from tumor draining LNs. Data shown as mean ± SEM with data points representing individual animals. Statistics calculated with MWU test, **p<0.01, ***p<0.001.

**4 Figure 3B, there is a discrepancy between the presented data and the statistical analysis. The representative plot on the left shows nearly identical percentages of CD122+CD44+ Tvm cells in middle-aged females (14%) and males (13%), yet the statistical graph on the right indicates a significant difference between the two groups.**

We thank the reviewer for pointing this out. We agree that the previously shown representative plots of middle-aged females and male Tvms were not fully representative of the underlying dataset. We have therefore replaced them with examples that more accurately reflect the group-level distributions.

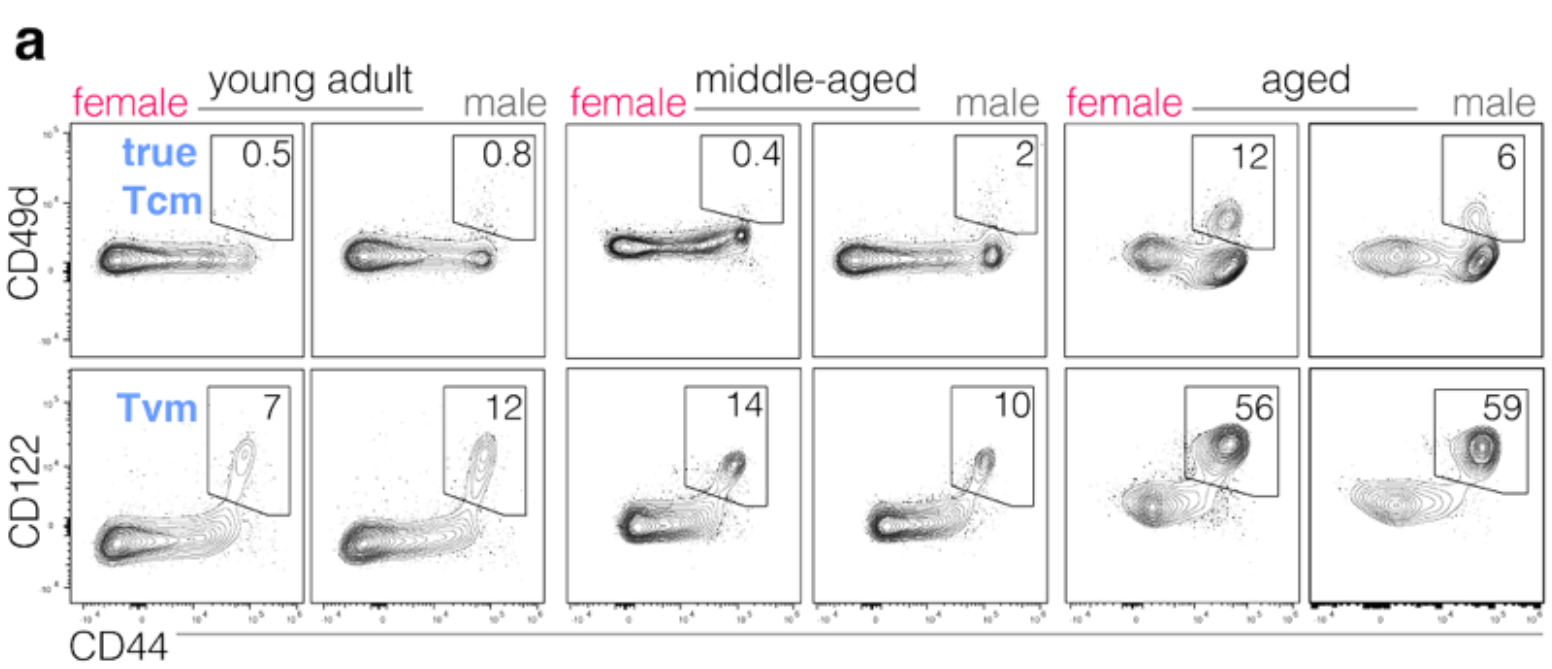


**Rebuttal letter Fig. 9. and Fig. 3b of the revised manuscript.** Tvm cells in ingLNs of WT mice. Gating strategy in **Extended Data Fig. 6f**.

**Reviewer #2 (Remarks to the Author):**

**Menzel et al present an interesting manuscript in which the experiments are well performed and the results important for our understanding of ageing, sex differences in immunity, and when interventions to promote immune health can be effective. The involution of the thymus is one of the best described age-associated immune traits, and yet multiple groups have shown that in advanced aged thymus rejuvenation is insufficient to enhance immune responses. In this manuscript, Padera and colleagues clearly show that impaired thymus function in mid-life males contributes to poor immunity, and that this can be corrected by limiting androgen availability. The mechanistic work is done in mice, with nice human correlate data included to demonstrate that these findings are likely relevant for human health. Overall, this study brings an important new finding to our understanding of thymus involution, and when targeting this can be beneficial for health. There are some minor changes that should be considered prior to publication.**

**-In the title "aging" should be replaced with "mid-life" (or similar) to better represent the conclusions**

We thank the reviewer for this suggestion. We agree that the key phenotype becomes most apparent during the transition to middle-aged/mid-life. Our data support a progressive, sex-biased divergence in naive CD8 T cell dynamics that begins earlier (young adults) and unfolds during aging to middle-aged. To better reflect this temporal aspect while maintaining the broader relevance of the findings, we have revised the title to:

"Lymph node contraction links sex-biased naive CD8 T cell decline to compromised antigen recognition during middle age."

**-In the abstract consider replacing "tumor recognition" with "response to checkpoint blockade" (or similar) as this is the most compelling pre-clinical data.**

We thank the reviewer for this suggestion. We have revised the abstract to include the impact on checkpoint blockade responses while maintaining a focus on the broader implications of our findings for antigen recognition across the lifespan.

Changes in the text, line 34-347: "Therapeutic thymus regeneration via androgen ablation repopulates naive CD8 T cells in lymph nodes, which reinvigorates cancer-specific T cell responses and enhances responsiveness to immune checkpoint blockade"

**-Include the age of the mice in months in the first paragraph of the results when describing "middle-aged" to make it easier to determine the age of the animals used. Including this information in the figure legends will improve readability.**

We thank the reviewer for this suggestion. We have now included the age of the mice in months when first introducing "middle-aged" animals in the Results, and have added this information to the relevant figure legends to improve clarity.

Changes in the text, line 108-110: "To assess CD8 T cell-mediated tumor control across age and sex, we compared tumor growth in young adult (7-10 weeks) and middle-aged (9-12 months) mice (**Extended Data Fig. 1b)**."

**-The extrapolation of mouse results to human lymph nodes leading to the conclusion "which indicates that, despite confounding factors, imaging-based LN volume measurements in humans reflect the underlying cellularity. The structure and composition of mouse and humans lymph nodes are different.**

We thank the reviewer for this comment. We agree that mouse and human lymph nodes differ in their structural organization and cellular composition, and we did not intend to imply otherwise. To avoid any ambiguity, we have removed the concluding statement about human imaging-based LN volume reflecting cellularity, as we do not have direct human LN cellularity data to support this claim. The mouse correlation data are presented on their own terms, and the human CT imaging data are described elsewhere as exploratory population-level observations.

**-Clarify why an anti-CD40 antibody was used during PR8 immunisation, as this is an unusual immunisation strategy.**

We thank the reviewer for this important comment. Agonistic CD40 stimulation is a well-established strategy to enhance T cell priming by licensing dendritic cells, mimicking CD4 T cell help, and promoting efficient cross-priming of CD8 T cells. This approach has been used in vaccination and tumor immunology settings to induce robust and reproducible T cell responses (Auten et al., 2012; Nimanong et al., 2017; Ostroumov et al., 2025).

Changes in the methods text, line 688-691: "…or PR8 virus particles ($1\times10^5$ inactivated particles with 2 µg poly(I:C) and 2 µg anti-CD40 mAb [clone MR-1, Bio X Cell]) or respective controls were injected intradermally into the lower flank. Anti-CD40 antibody was included to mimic CD4 T cell help and promote efficient DC activation and cross-priming."

**-Figure 2b, the data are not continuous for age, but are in discrete groups which means linear regression is not the ideal way to analyse them statisitcally.**

We thank the reviewer for this comment. We agree that age was analyzed in discrete groups. To address this, we have adjusted the statistical analysis accordingly and clarified this in the figure legend. Statistical comparisons are based on appropriate non-parametric analyses (Spearman correlation).

Changes in the text, line 1212: "Statistical calculation performed by Spearman correlation (b,d)"

**-In graphs with multiple comparisons has multiple testing correction been performed? The figure legend implies not.**

We thank the reviewer for this comment. Multiple comparisons were not performed unless explicitly indicated. Longitudinal datasets were analyzed using repeated measures ANOVA with Geisser-Greenhouse correction, using the interaction between time and experimental group to assess differences between groups.

Changes in the text, line 889-892: “Tumor growth curves were analyzed using two-way repeated measures ANOVA with Geisser-Greenhouse correction, with the interaction between time and group used to assess differences in growth dynamics between experimental groups. Multiple pairwise comparisons were not performed unless explicitly indicated.”

**-In 6h, Interquartile Ranges (25th–75th percentiles) or 95% Confidence Intervals of the median, rather than SEM is a more appropriate way to represent the variance of this data type (assuming non-normal distribution because MWU is the test used).**

We thank the reviewer for this statistical suggestion. We believe the reference to "Fig. 6h" may contain a cross-referencing error, as Fig. 6h shows tumor growth curves analyzed by two-way repeated measures ANOVA for which SEM is appropriate. We suspect the comment is directed at our human CT-based lymph node volume data **(Fig. 2a,b)** and human naive CD8 T cell count data (**Fig. 5b**), where the reviewer's point is well taken. These data are derived from a clinical imaging cohort and normal distribution cannot be assumed. We have therefore switched the representation of these datasets to interquartile ranges, which is more appropriate given the use of the Mann-Whitney U test, and have updated the figure legend accordingly.

**Reviewer #3 (Remarks to the Author):**

**A. Summary of the key results:**

**This study explores sex- and age-dependent changes in naive $CD8^+$ T cell dynamics and their impact on anti-tumor immunity. The authors show that male mice experience an earlier and more pronounced decline in naive $CD8^+$ T cells compared to females, accompanied by lymph node contraction and reduced antigen recognition capacity. Mechanistically, this phenotype is associated with both reduced thymic output and increased differentiation into virtual memory T (Tvm) cells. Importantly, androgen blockade partially restores thymic function, expands the naive $CD8^+$ T cell pool, and improves tumor control in middle-aged males. These findings provide a coherent framework linking immune aging, sex differences, and tumor immunity.**

**B. Originality and significance:**

**The manuscript addresses a timely and important question regarding sex differences in immune aging and their relevance to cancer immunity. The integration of thymic involution, Tvm differentiation, and lymph node remodeling is conceptually appealing. While individual components of the model have been described previously, the study provides a valuable integrative perspective, particularly in highlighting sex-biased timing and functional consequences for tumor immunity. The work, therefore, represents a meaningful extension of existing knowledge with potential translational implications.**

**C. Data & methodology:**

**The study employs a broad range of experimental approaches, including murine tumor models, flow cytometry, TCR analyses, and reanalysis of human datasets. The overall design is comprehensive, and the data are generally of good quality. The main area where the manuscript could be strengthened is in clarifying mechanistic relationships:**

**1.Role of naive $CD8^+$ T cells in tumor immunity: The data support a strong association between reduced naive $CD8^+$ T cell numbers and impaired tumor control. It would be helpful to further clarify to what extent this reduction is directly limiting for anti-tumor responses versus reflecting broader immune remodeling.**

We thank the reviewer for this important point. We agree that clarifying the mechanistic link between naive CD8 T cell availability and impaired anti-tumor immunity strengthens the central message of the study. To address this, we have revised the relevant sections of the results and discussion to more explicitly articulate how reduced naive T cell numbers are expected to directly constrain anti-tumor responses, rather than merely reflecting broader immune remodeling.

Changes in the results, line 199-203: “These findings support a model in which reduced availability of naive CD8 T cells limits the probability of recruiting cancer-reactive clones. This is consistent with recent work showing that a diminished naive T cell pool is expected to directly constrain the formation and diversity of these precursor populations, thereby reducing the

likelihood of effective cancer-specific T cell responses (Hor et al., 2025; Salazar-Cavazos et al., 2026)."

Changes in the discussion, line 443-449: "Recent studies (Hor et al., 2025; Salazar-Cavazos et al., 2026) have shown that tdLNs continuously generate stem-like CD8 T cell populations that serve as a renewable source of effector cells and are enriched for high-affinity clones. In this context, the size and diversity of the naive T cell pool represent a key upstream determinant of the landscape from which these stem-like CD8 T cell populations are derived. Thus, reduced naive T cell availability can constrain anti-tumor immunity by limiting the generation of high-affinity, stem-like T cell clones capable of sustaining effective responses."

Finally, we have expanded the introduction to provide stronger contextual grounding for CD8 T cell-dependent anti-cancer immunity, with additional references supporting this paradigm. Changes in the introduction, line 59-63: "Recent work has begun to clarify why the numerical availability of naive CD8 T cells in draining LNs is crucial for anti-cancer immunity. Anti-cancer immune responses are inherently stochastic and driven by rare, functionally critical T cells. As the size of the naive T cell precursor pool available for priming decreases, the probability of these rare cells being available for activation also decreases (Hor et al., 2025; Salazar-Cavazos et al., 2026)."

**2.Relationship between thymic output and Tvm differentiation:**

**The proposed model combining reduced thymic output and increased Tvm differentiation is compelling. Additional clarification-either experimentally or through discussion-of how these processes are linked would improve the conceptual clarity.**

We thank the reviewer for highlighting the importance of clarifying the relationship between reduced thymic output and increased virtual memory (Tvm) differentiation. We agree that, while both processes are supported by our data, their integration into a coherent framework would benefit from clearer conceptual articulation.

Mechanistically, these two processes operate on opposite sides of the same balance: thymic output determines the rate of naive CD8 T cell replenishment, while antigen-agnostic Tvm conversion is one factor that determines the rate of peripheral naïve T cell attrition along with general antigen-dependent T cell activation and a low level of natural T cell senescence (Goronzy and Weyand, 2019; Mittelbrunn and Kroemer, 2021; Quinn et al., 2018). However, these processes are not independent, their interaction is what makes the sex-biased phenotype emerge. In a setting of normal thymic output, ongoing Tvm conversion would be well tolerated, as de novo naive T cells continuously replenish the pool. Conversely, peripheral Tvm conversion is what makes declining thymic output consequential. Without ongoing attrition, the long lifespan of naive T cells would buffer against even substantially reduced thymic replenishment. It is the combination of accelerated early-life Tvm conversion, occurring at a higher rate in males, acting in a background of progressively limited thymic replenishment that produces the cumulative, sex-biased divergence in naive CD8 T cell numbers. Thymic involution thus defines the ceiling of supply, while Tvm conversion determines how rapidly that supply is eroded. This framework is now explicitly described in the revised Discussion and summarized in the new schematic **(Rebuttal letter Fig. 7 and Fig. 6i)**.

Changes in the discussion, line 468-472: “Peripheral Tvm conversion is what makes declining thymic output consequential. Without ongoing attrition, the long lifespan of naive T cells would buffer against even substantially reduced thymic replenishment, and the sex-biased divergence we observe would not emerge on the timescale of middle age.”

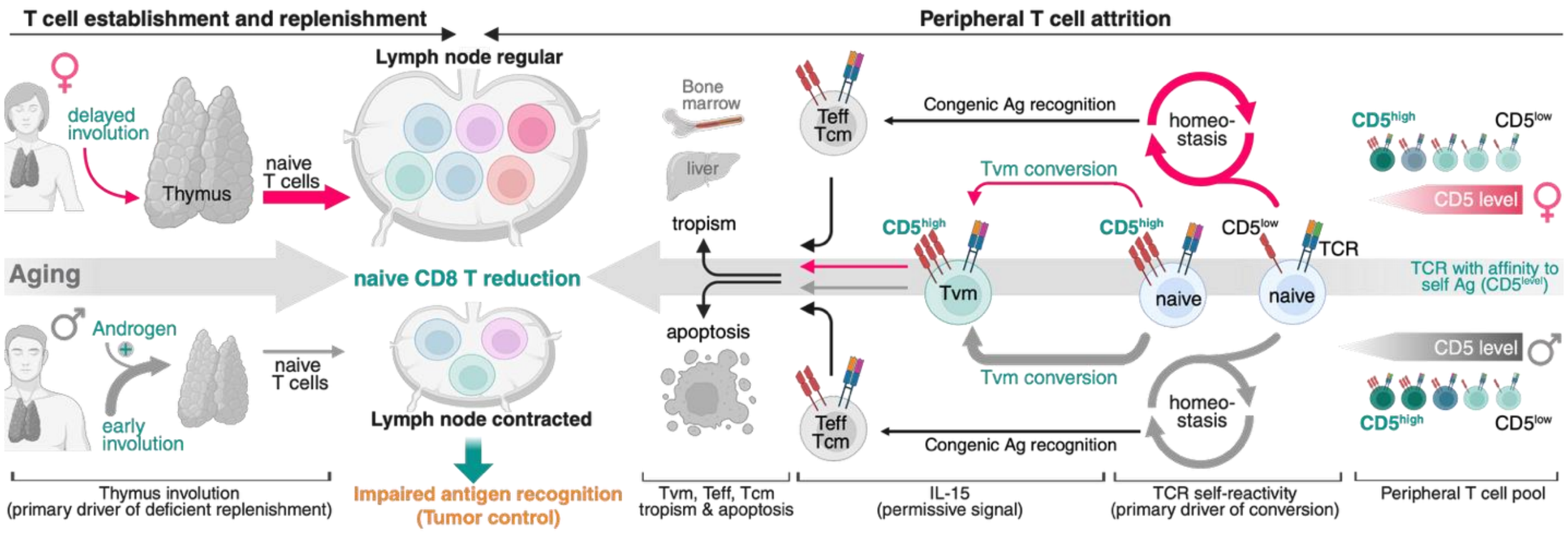


**Rebuttal letter Fig. 10 and Fig. 6i of the revised manuscript.** Conceptual model linking sex-biased thymic involution and TCR self-reactivity–driven Tvm conversion to naive $CD8^+$ T cell loss, LN contraction, and impaired antigen recognition.

### 3.Tvm functional properties:

**The study demonstrates system-level consequences of Tvm expansion. Providing additional insight into the functional characteristics of Tvm cells (e.g., effector potential or contribution to immune regulation) would further strengthen the mechanistic interpretation.**

We thank the reviewer for this suggestion. To address this question, we performed complementary effector function, proliferation and transcriptomic analyses **(Rebuttal letter Fig. 11a)**.

Compared to naive cells, TVM cells display enhanced effector cytokine production upon stimulation, especially regarding the expression of IFNγ **(Rebuttal letter Fig. 11b-d)**. Following 5 days of CD3/CD28 stimulation, middle-aged Tvm cells exhibited a significant age-associated decline in proliferative capacity that was independent of sex, whereas proliferation of naive CD8 T cells remained preserved with aging. In contrast, Tvm cells from young adult mice displayed proliferative responses comparable to those of naive cells **(Rebuttal Fig. 11e)**.

We performed bulk RNA sequencing of sorted naive and Tvm CD8 T cells from young adult and middle-aged male and female mice, unstimulated or after short-term stimulation **(Rebuttal Fig. 11a).** Principal component analysis revealed that the overall transcriptional identity is primarily defined by naive vs Tvm (62% proportion of variance) and stimulation groups (17%). Age influences the distribution of Tvm but not of the naïve CD8 T cells **(Rebuttal Fig. 11f)**. Consistent with the higher apoptotic rate observed in Tvm cells **(Extended Data Fig. 9d)**, gene set scoring across senescence-associated hallmarks revealed that middle-aged Tvm cells upregulate apoptosis, senescence and terminal differentiation signatures compared to naive cells **(Rebuttal Fig. 11g)**.

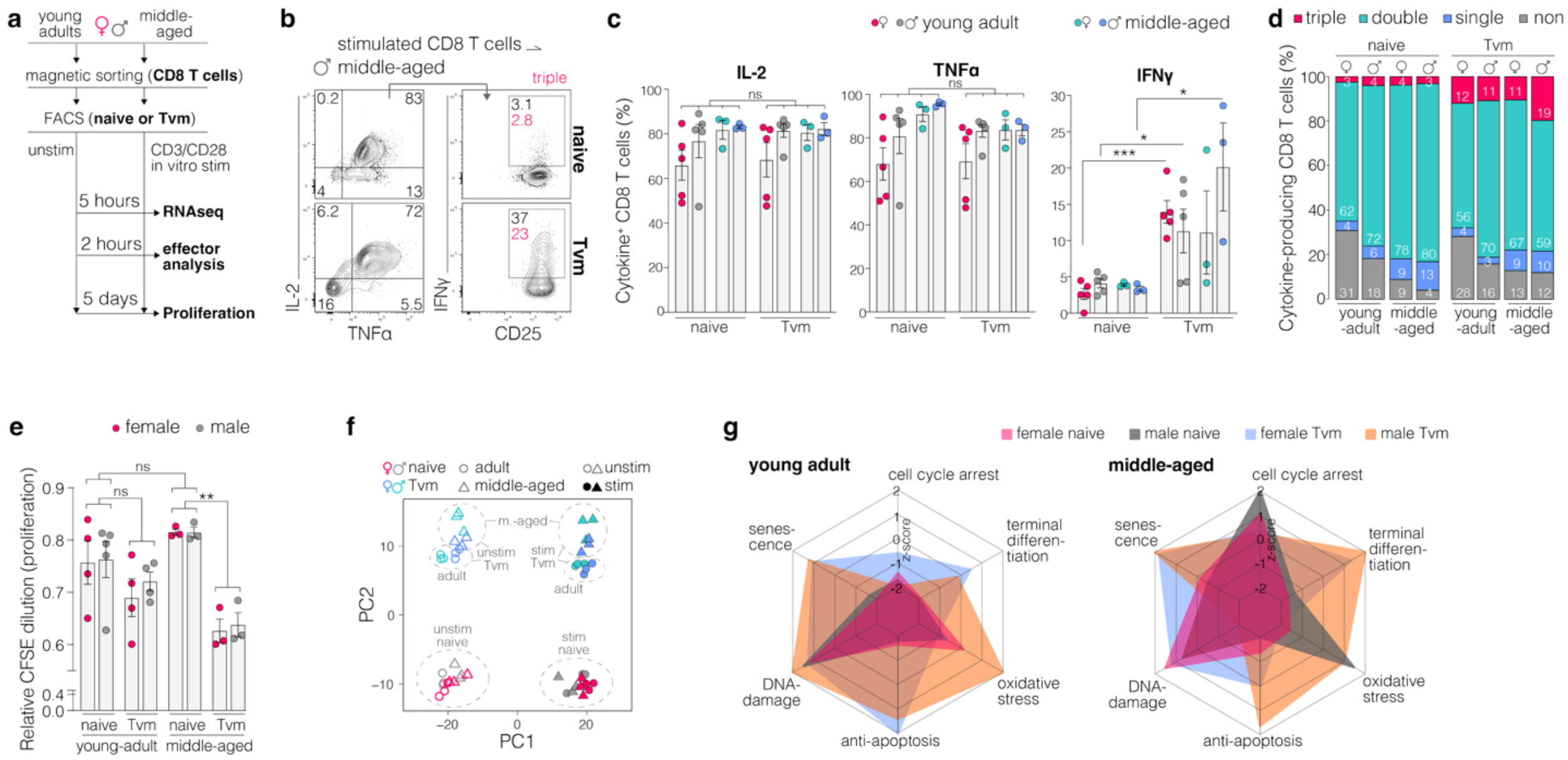


**Rebuttal letter Fig. 11 and Fig. 4a-c of the revised manuscript. a, Experimental setup. b**, Example plots and **c**, quantification of cytokine production in subsets of CD8 T cells after in vitro stimulation. **d (Fig. 4a),** Polyfunctional cytokine production in subsets of CD8 T cells after in vitro stimulation, showing the proportion of cells producing combinations of IL-2, IFNγ, and TNF as measure for effector capacity. **e (Fig. 4b)**, Proliferation capacity of CD8 T cell subsets after in vitro stimulation, quantification depicted by dilution of CFSE MFI relative to unstimulated control. **f**, Principal component (PC) analysis of VST-normalized bulk RNA-seq counts across all 48 samples. **g (Fig. 4c)**, Radar plots showing z-scored mean expression of senescence-associated gene sets in young adult and middle-aged naive and Tvm CD8 T cells across all groups. Gene sets described in material and methods.

**4.Human data interpretation: The human analyses are supportive and increase translational relevance. A clearer discussion of their limitations (e.g., cross-sectional nature, potential confounders) would improve interpretability.**

Thank you for this suggestion. We have made the following changes in the results, line 536-546: “The human T cell and thymus data in our study are derived from at least two independent cohorts, strengthening the translational relevance of our findings. The CT-based LN volume data are inherently more exploratory. The scans were obtained from individuals with unknown health indications, volumes are unadjusted for body composition or underlying conditions, and LN size and number can vary substantially between individual humans compared to mice. Nevertheless, averaging volumetric measurements across anatomically defined LN regions and using three-dimensional volume rendering show the sex- and age-related trends we observe are consistent with our murine and human immune cell data. These convergent lines of evidence raise the possibility that thymus-targeted interventions, such as short-term SSA treatment, could be explored in selected clinical contexts where insufficient priming constrains immunotherapy responses.”

**D. Appropriate use of statistics and treatment of uncertainties**

**The statistical analyses appear appropriate overall. To further strengthen the rigor, the authors may consider:**

**1)Providing more emphasis on effect sizes alongside statistical significance.**

We thank the reviewer for this suggestion. We agree that effect sizes provide important context beyond statistical significance alone and we have revised the manuscript to include them at three key points where biological magnitude is particularly informative.

Changes in the text, line 252-255: "Although the difference in $CD5^{high}$ naive CD8 T cell frequency between young adult females and males is modest (approximately 0.7% vs. 1.1%), these cells arise from a compartment comprising >85% of all CD8 T cells, resulting in a substantial absolute pool of higher self-reactive clones available for Tvm conversion."

Changes in the text, line 394-399: "This translated into a significant restoration of naive CD8 T cell numbers in middle-aged males, recovering from approximately $3x10^5$ cells/ingLN to $8x10^5$cells/ingLN, approaching levels seen in young adults (~$10^6$ cells/ingLN) and untreated middle-aged females. In aged males, DeX treatment also increased naive CD8 T cells, but to a lesser extent, and absolute numbers ($2x10^5$ cells/ingLN) remained reduced relative to middle-aged mice (**Fig. 6f; Extended Data Fig. 13a-l**)."

Changes in the text, line 171-177: "However, in our data we observe quantitative stability or loss of $CD44^+CD62L^+$ memory-phenotype cells and a clear loss of naive CD8 T cells in inguinal LNs (ingLN) beginning shortly after young adulthood, with naive cells showing a particularly pronounced reduction in males compared to females, declining from ~$10^6$ cells/ingLN in young adults of both sexes to ~$8x10^5$ cells/ingLN in middle-aged females and ~$4x10^5$ cells/ingLN in middle-aged males, with both sexes converging with advanced age **(Fig. 2e)**."

**2) Clarifying how multiple comparisons were handled where applicable.**

We thank the reviewer for this comment that was also raised by Reviewer 2. Multiple comparisons were not performed unless explicitly indicated. Longitudinal datasets were analyzed using repeated measures ANOVA with Geisser-Greenhouse correction, using the interaction between time and experimental group to assess differences between groups.

Changes in the text, line 889-892: "Tumor growth curves were analyzed using two-way repeated measures ANOVA with Geisser-Greenhouse correction, with the interaction between time and group used to assess differences in growth dynamics between experimental groups. Multiple pairwise comparisons were not performed unless explicitly indicated."

**3) Expanding discussion of variability in human datasets**

We thank the reviewer for this comment. We refer the reviewer to our response to comment C.4 above, where we address the limitations and variability of the human datasets in detail.

**These points are relatively minor but would enhance transparency.**

**E. Conclusions: robustness, validity, reliability**

**The conclusions are generally supported by the data and are biologically plausible. Some aspects of the interpretation—particularly regarding mechanistic causality and translational implications—could be presented with slightly more caution to better reflect the current level of evidence.**

We thank the reviewer for this comment. We have carefully reviewed the manuscript with this concern in mind.

Specific revisions include softening "cannot be compensated" to "is insufficiently compensated" (lines 468) to better reflect the correlative rather than strictly causal nature of the thymic involution data and ensuring that translational claims regarding checkpoint blockade are consistently framed as exploratory, as reflected in the revised clinical paragraph (lines 443-460): "Recent studies (Hor et al., 2025; Salazar-Cavazos et al., 2026) have shown that tdLNs continuously generate stem-like CD8 T cell populations that serve as a renewable source of effector cells and are enriched for high-affinity clones. In this context, the size and diversity of the naive T cell pool represent a key upstream determinant of the landscape from which these stem-like CD8 T cell populations are derived. Thus, reduced naive T cell availability can constrain anti-tumor immunity by limiting the generation of high-affinity, stem-like T cell clones capable of sustaining effective responses. Importantly, this mechanism reflects a fundamental relationship between naive CD8 T cell availability in LNs and the stochastic probability of antigen recognition. In this framework, the tumor serves primarily as a source of locoregional antigen, while the LN naive T cell pool determines the likelihood that a cognate clone will be recruited (Salazar-Cavazos et al., 2026). Tumor immunogenicity acts as a modulating variable in this relationship, as a higher density of neo-antigens and antigens with specific recognition motifs (Dolton et al., 2023) can partially compensate for a reduced naive precursor pool, demonstrated in the context of poorly versus highly immunogenic tumors (Escobar et al., 2023). In support of the broader applicability of this framework, age-related impairment of tumor control has been documented across a range of syngeneic tumor models with and without immunogenic modifications and consistently implicate loss of tumor-specific CD8 T cell responses as a shared underlying mechanism (Chen et al., 2024; Georgiev et al., 2024)."

Additionally, the role of IL-15 in sex-biased naive CD8 T cell decline has been more clearly explained. We have revised the relevant sections to clarify that IL-15 acts as a permissive baseline signal required for Tvm maintenance rather than a primary driver of sex- or age-dependent differences. This description is more consistent with our interpretation of our data and the role for IL-15 in Tvm maintenance (line 275-282): "To further establish that Tvm formation can lead to depletion of naive CD8 T cell populations, we used IL-15/IL-15 receptor alpha complexes (IL-15 Cx), the biologically most active form of IL-15, to experimentally drive Tvm formation in young adults. As illustrated in **Fig. 4d**, IL-15 does not directly induce Tvm differentiation but acts as a permissive co-stimulatory signal that lowers the activation threshold of self-reactivity ($CD5^{level}$) of naive CD8 T cells for converting into Tvm cells (Hussain et al., 2025, 2023; Hussain and Quinn, 2019; White et al., 2016). Thus, IL-15 Cx enables experimental lowering of the self-affinity threshold for Tvm formation in a setting with preserved TCR diversity but low baseline conversion."

Overstatement of the mechanistic link between the naive T cell pool and de novo synthesis in the thymus was toned down (line 486-490): "Consequently, while the systemic population of CD4 T cells remains relatively stable over time(Mold et al., 2019), independent of age and sex, naive CD8 T cells are more prone to Tvm formation, senescence and apoptosis, suggesting that maintaining an abundant and clonally diverse pool of naive CD8 T cells becomes increasingly dependent on de novo thymic synthesis with age."

We changed overly strong wording like “effectively recapitulating” to “mimicking key features” (line 308-311): “These findings demonstrate that transient induction of Tvm cells in young adult female mice preserves short-term anti-tumor effector function but accelerates long-term naive CD8 T cell attrition, thereby mimicking key features of male-biased immune aging.”

Instead of stating direct causality of impaired tumor recognition due to naive to Tvm conversion and a reduced abundance of naive T cell clones in LNs, we changed to a more correlative phrasing that is backed by our data (line 80-84): “The differentiation into virtual memory T cells, which are shorter-lived and more prone to senescence (Quinn et al., 2018), reduces the abundance of unique naive T cell clones in LNs and is associated with impaired cancer recognition. Experimental thymus regeneration in male mice was able to restore the source of naive CD8 T cells and improved cancer-targeted responses in middle-aged, but not aged, mice.”

We changed “defined therapeutic window” to “a potential therapeutic window” (line 508-511): “These data underscore the potential of therapeutic interventions targeting thymic regeneration, especially within a potential therapeutic window before advanced age-related changes such as thymus epithelial cell senescence, LN stromal fibrosis and structural degeneration irreversibly hinder regeneration efforts (Kousa et al., 2024; Masters et al., 2019; Thompson et al., 2019).”

**F. Suggested improvements**

**The manuscript is already strong, and the following suggestions are intended to further enhance clarity and impact:**

**1.Further support for the role of naive CD8$^{+}$ T cells:**

**Additional evidence (experimental or discussion-based) clarifying their contribution to tumor immunity would strengthen the central message.**

We added the following paragraph highlighting the role of a diverse and sufficiently abundant pool of naive and progenitor CD8 T cells (lines 443-460): “Recent studies (Hor et al., 2025; Salazar-Cavazos et al., 2026) have shown that tdLNs continuously generate stem-like CD8 T cell populations that serve as a renewable source of effector cells and are enriched for high-affinity clones. In this context, the size and diversity of the naive T cell pool represent a key upstream determinant of the landscape from which these stem-like CD8 T cell populations are derived. Thus, reduced naive T cell availability can constrain anti-tumor immunity by limiting the generation of high-affinity, stem-like T cell clones capable of sustaining effective responses. Importantly, this mechanism reflects a fundamental relationship between naive CD8 T cell availability in LNs and the stochastic probability of antigen recognition. In this framework, the tumor serves primarily as a source of locoregional antigen, while the LN naive T cell pool determines the likelihood that a cognate clone will be recruited (Salazar-Cavazos et al., 2026). Tumor immunogenicity acts as a modulating variable in this relationship, as a higher density of neo-antigens and antigens with specific recognition motifs (Dolton et al., 2023) can partially compensate for a reduced naive precursor pool, demonstrated in the context of poorly versus highly immunogenic tumors (Escobar et al., 2023). In support of the broader applicability of this framework, age-related impairment of tumor control has been documented across a range of syngeneic tumor models with and without immunogenic modifications and consistently implicate loss of tumor-specific CD8 T cell responses as a shared underlying mechanism (Chen et al., 2024; Georgiev et al., 2024).”

**2.Expanded characterization of Tvm cells:**

**Additional functional insights (if available) or discussion of their potential roles would improve mechanistic depth.**

We thank the reviewer for this comment. We refer the reviewer to our response to comment C.3 above, where we addressed the functional properties of Tvm cells with new experimental data and discussion, included in **(Fig. 4a-c and Extended Data Fig. 9a-c)** of the revised manuscript.

**3.Clarification of the proposed model:**

**A more explicit conceptual model describing the interplay between thymic involution, Tvm differentiation, and lymph node changes would improve readability.**

We thank the reviewer for this suggestion. This point is addressed by the inclusion of a schematic model summarizing the proposed mechanistic framework in **Rebuttal letter Fig.10 and Fig. 6i** of the revised manuscript, as described above.

**4.Contextualization of human findings:**

**Briefly outlining limitations and potential confounders would strengthen translational interpretation.**

We thank the reviewer for this comment. We refer the reviewer to our response to comment C.4 above, where we address the limitations and variability of the human datasets in detail.

**5.Broader validation:**

**Inclusion of an additional tumor model would be helpful, but is not essential.**

We thank the reviewer for this suggestion. We respectfully note that the mechanistic framework proposed in our study is not contingent on a specific tumor model, but rather reflects a fundamental relationship between naive CD8 T cell availability in lymph nodes and the stochastic probability of antigen recognition. In this framework, the tumor serves primarily as a source of locoregional antigen, while the lymph node naive T cell pool determines the likelihood that a cognate clone will be recruited. The stochastic nature of this encounter, and its dependence on naive pool size, has recently been formally demonstrated by Salazar-Cavazos et al. 2026, further supporting the generalizability of our model beyond any single tumor context.

We do agree, however, that cancer immunogenicity is an important modulating variable, as higher density of neo-antigens (Dolton et al., 2023) can partially compensate for a reduced naive precursor pool, a concept elegantly demonstrated by Escobar et al. 2023 in the context of B16-OVA (immunogenicity relatively low) and MC38-OVA adenocarcinoma (immunogenicity relatively high). Our model predicts that the functional consequences of naive T cell decline will be most pronounced in settings where antigen presentation and CD8 T cell priming is rate-limiting, consistent with our use of the B16-OVA model.

To further address the reviewer's concern regarding generalizability, we note that age-related differences in tumor control has been demonstrated across a broad range of syngeneic tumor models, including B16 melanoma, MC38 adenocarcinoma, and E0771 breast cancer, with and without immunogenic modifications. All these models consistently implicate the loss of cancer-specific CD8 T cell responses as a shared underlying mechanism (Georgiev et al., 2024). The

contribution of androgens to sex-biased CD8 T cell dysfunction has similarly been documented across multiple tumor models and cancer types (Kwon et al., 2022; Yang et al., 2020). Together, these convergent findings across diverse experimental systems support the broad applicability of our proposed mechanism. We have included a discussion of these supportive findings in published data in lines 443-460: "Recent studies (Hor et al., 2025; Salazar-Cavazos et al., 2026) have shown that tdLNs continuously generate stem-like CD8 T cell populations that serve as a renewable source of effector cells and are enriched for high-affinity clones. In this context, the size and diversity of the naive T cell pool represent a key upstream determinant of the landscape from which these stem-like CD8 T cell populations are derived. Thus, reduced naive T cell availability can constrain anti-tumor immunity by limiting the generation of high-affinity, stem-like T cell clones capable of sustaining effective responses. Importantly, this mechanism reflects a fundamental relationship between naive CD8 T cell availability in LNs and the stochastic probability of antigen recognition. In this framework, the tumor serves primarily as a source of locoregional antigen, while the LN naive T cell pool determines the likelihood that a cognate clone will be recruited (Salazar-Cavazos et al., 2026). Tumor immunogenicity acts as a modulating variable in this relationship, as a higher density of neo-antigens and antigens with specific recognition motifs (Dolton et al., 2023) can partially compensate for a reduced naive precursor pool, demonstrated in the context of poorly versus highly immunogenic tumors (Escobar et al., 2023). In support of the broader applicability of this framework, age-related impairment of tumor control has been documented across a range of syngeneic tumor models with and without immunogenic modifications and consistently implicate loss of tumor-specific CD8 T cell responses as a shared underlying mechanism (Chen et al., 2024; Georgiev et al., 2024)."

**G. References: appropriate credit to previous work**

**The manuscript cites relevant literature. It would be beneficial to more explicitly position the current findings within the broader context of:**

We thank the reviewer for this suggestion and added the following parts to the discussion:

**1)Immune aging and thymic involution**

Changes in the text of the discussion line 420-426: "Age-related thymic involution and its consequences for peripheral T cell homeostasis have been studied for decades, with foundational work establishing that progressive loss of thymic output leads to numerical and functional decline of naive T cells and contraction of TCR diversity with age (Bernatz et al., 2026a; Gudmundsdottir et al., 2016; Kooshesh et al., 2023). More recent clinical evidence has linked thymic atrophy directly to increased cancer risk and mortality in humans (Bernatz et al., 2026a, 2026b; Kooshesh et al., 2023), elevating this process—once-considered passive—to an active determinant of immune competence."

**2) Virtual memory T cell biology**

Changes in the text of the discussion line 266-274: "Compared to naive cells, Tvm cells display enhanced effector cytokine production upon stimulation, yet lose proliferative capacity, upregulate senescence-associated transcriptional signatures **(Fig. 4a-c, Extended Data Fig. 9a-c)**, and increase apoptosis rates with aging (**Extended Data Fig. 9d)** with minimal age and sex dependent metabolic differences **(Supplementary Fig. 2)**. LNs continue to shrink with age

despite Tvm formation, as Tvm cells have a shorter lifespan due to increased apoptosis (Moskowitz et al., 2017; Quinn et al., 2018), and preferentially relocate to the spleen, bone marrow **(Extended Data Fig. 9e)** or liver(White et al., 2016). Both of these processes contribute to a sex-biased loss of naive CD8 T cells and LN contraction earlier in life."

To further establish that Tvm formation can lead to depletion of naive CD8 T cell populations, we used IL-15/IL-15 receptor alpha complexes (IL-15 Cx)—the biologically most active form of IL-15—to experimentally drive Tvm formation in young adults. As illustrated in **Fig. 4d**, IL-15 does not directly induce Tvm differentiation but acts as a permissive co-stimulatory signal that lowers the activation threshold of self-reactivity ($CD5^{level}$) of naive CD8 T cells for converting into Tvm cells (Hussain et al., 2025, 2023; Hussain and Quinn, 2019; White et al., 2016). Thus, IL-15 Cx enables experimental lowering of the self-affinity threshold for Tvm formation in a setting with preserved TCR diversity but low baseline conversion."

**3) Sex differences in immune responses to better highlight the study's contribution.**

Changes in the text of the discussion line 45-47: **"**Addressing these gaps may reveal strategies to restore immune competence and improve cancer outcomes across aging populations in a sex-specific manner."

and line 465-474: "In line with a recent publication (Kooshesh et al., 2023), our data refine this paradigm by demonstrating a selective depletion of naive CD8 T cells, driven in part by premature differentiation into Tvm cells. This loss is insufficiently compensated in males due to earlier thymic involution. Peripheral Tvm conversion is what makes declining thymic output consequential. Without ongoing attrition, the long lifespan of naive T cells would buffer against even substantially reduced thymic replenishment, and the sex-biased divergence we observe would not emerge on the timescale of middle age. The sex-biased reduction in naive CD8 T cells profoundly reduces the numerical abundance of unique clones, critically impairing antigen recognition capacity in tumor-draining LNs."

**H. Clarity and context**

**The manuscript is generally well written and clearly structured. The central message is understandable.**
**Minor improvements that could further enhance clarity include:**

**1)Presenting the mechanistic framework more explicitly.**

We thank the reviewer for this suggestion. This point is addressed by the inclusion of a schematic model summarizing the proposed mechanistic framework in **Rebuttal letter Fig.10 and Fig. 6i** of the revised manuscript, as described above.

**2)Simplifying some dense sections in the Results.**

We thank the reviewer for this suggestion. We have revised the Results to improve readability in the denser sections, particularly the passages describing the CD5-based analysis of TCR self-reactivity and the rationale for the IL-15 complex experiments, where we have separated mechanistic explanation from data description more clearly and streamlined transitions between concepts.

**3)Slightly tempering speculative statements in the Discussion.**

We thank the reviewer for this suggestion. We have carefully reviewed the Discussion with this concern in mind and made several targeted revisions to temper speculative language, as detailed in our response to comment E.

**Additional suggestion on presentation clarity**

**To further enhance the clarity of the proposed mechanistic framework, the authors may consider including a schematic diagram or workflow-style figure summarizing the overall experimental design and key biological relationships.**

**In particular, a visual representation integrating thymic involution, naive CD8$^{+}$ T cell decline, Tvm differentiation, lymph node contraction, and their collective impact on tumor immunity would greatly improve readability and help readers better follow the central model of the study.**
**Such a schematic would also be useful in clearly distinguishing experimentally supported relationships from more conceptual or inferred connections.**

We thank the reviewer for this suggestion. This point is addressed by the inclusion of a schematic model summarizing the proposed mechanistic framework in **Rebuttal letter Fig.10 and Fig. 6i** of the revised manuscript, as described above.

**This is a well-designed and insightful study that provides an integrated view of sex-biased immune aging and its impact on tumor immunity. The manuscript would benefit from modest improvements in mechanistic clarification and contextual framing, but the core findings are compelling.**

**Recommendation: Major Revision**

## References


Auten, M.W., Huang, W., Dai, G., Ramsay, A.J., 2012. CD40 ligand enhances immunogenicity of vector-based vaccines in immunocompetent and CD4+ T cell deficient individuals. Vaccine 30, 2768–2777. https://doi.org/10.1016/j.vaccine.2012.02.020

Bernatz, S., Prudente, V., Pai, S., Attermann, A.K., Cao, Y., Chen, J., Lyass, A., Foldyna, B., Nürnberg, L., Bressem, K., Abbosh, C., Swanton, C., Jamal-Hanjani, M., Lu, M.T., Murabito, J.M., Lunetta, K.L., Birkbak, N.J., Aerts, H.J.W.L., 2026a. Thymic health consequences in adults. Nature 1–9. https://doi.org/10.1038/s41586-026-10242-y

Bernatz, S., Prudente, V., Pai, S., Attermann, A.K., Di Federico, A., Rowan, A., Veeriah, S., Dyrskjøt, L., Nürnberg, L., Alessi, J.V., Ott, P.A., Sharon, E., Hackshaw, A., McGranahan, N., Abbosh, C., Mak, R.H., Bitterman, D., Awad, M., Ricciuti, B., Swanton, C., Jamal-Hanjani, M., Birkbak, N.J., Aerts, H.J.W.L., 2026b. Thymic health and immunotherapy outcomes in patients with cancer. Nature 1–9. https://doi.org/10.1038/s41586-026-10243-x

Chen, A.C.Y., Jaiswal, S., Martinez, D., Yerinde, C., Ji, K., Miranda, V., Fung, M.E., Weiss, S.A., Zschummel, M., Taguchi, K., Garris, C.S., Mempel, T.R., Hacohen, N., Sen, D.R., 2024. The aged tumor microenvironment limits T cell control of cancer. Nat Immunol 1–13. https://doi.org/10.1038/s41590-024-01828-7

Dolton, G., Rius, C., Wall, A., Szomolay, B., Bianchi, V., Galloway, S.A.E., Hasan, M.S., Morin, T., Caillaud, M.E., Thomas, H.L., Theaker, S., Tan, L.R., Fuller, A., Topley, K., Legut, M., Attaf, M., Hopkins, J.R., Behiry, E., Zabkiewicz, J., Alvares, C., Lloyd, A., Rogers, A., Henley, P., Fegan, C., Ottmann, O., Man, S., Crowther, M.D., Donia, M., Svane, I.M., Cole, D.K., Brown, P.E., Rizkallah, P., Sewell, A.K., 2023. Targeting of multiple tumor-associated antigens by individual T cell receptors during successful cancer immunotherapy. Cell 186, 3333-3349.e27. https://doi.org/10.1016/j.cell.2023.06.020

Drobek, A., Moudra, A., Mueller, D., Huranova, M., Horkova, V., Pribikova, M., Ivanek, R., Oberle, S., Zehn, D., McCoy, K.D., Draber, P., Stepanek, O., 2018. Strong homeostatic TCR signals induce formation of self-tolerant virtual memory CD8 T cells. The EMBO Journal 37, e98518. https://doi.org/10.15252/embj.201798518

Escobar, G., Tooley, K., Oliveras, J.P., Huang, L., Cheng, H., Bookstaver, M.L., Edwards, C., Froimchuk, E., Xue, C., Mangani, D., Krishnan, R.K., Hazel, N., Rutigliani, C., Jewell, C.M., Biasco, L., Anderson, A.C., 2023. Tumor immunogenicity dictates reliance on TCF1 in CD8+ T cells for response to immunotherapy. Cancer Cell 41, 1662-1679.e7. https://doi.org/10.1016/j.ccell.2023.08.001

Georgiev, P., Han, S., Huang, A.Y., Nguyen, T.H., Drijvers, J.M., Creasey, H., Pereira, J.A., Yao, C.-H., Park, J.S., Conway, T.S., Fung, M.E., Liang, D., Peluso, M., Joshi, S., Rowe, J.H., Miller, B.C., Freeman, G.J., Sharpe, A.H., Haigis, M.C., Ringel, A.E., 2024. Age-associated contraction of tumor-specific T cells impairs antitumor immunity. Cancer Immunology Research. https://doi.org/10.1158/2326-6066.CIR-24-0463

Goronzy, J.J., Weyand, C.M., 2019. Mechanisms underlying T cell ageing. Nat Rev Immunol 19, 573–583. https://doi.org/10.1038/s41577-019-0180-1

Gudmundsdottir, J., Óskarsdóttir, S., Skogberg, G., Lindgren, S., Lundberg, V., Berglund, M., Lundell, A.-C., Berggren, H., Fasth, A., Telemo, E., Ekwall, O., 2016. Early thymectomy leads to premature immunologic ageing: An 18-year follow-up. Journal of Allergy and Clinical Immunology 138, 1439-1443.e10. https://doi.org/10.1016/j.jaci.2016.05.014

Haluszczak, C., Akue, A.D., Hamilton, S.E., Johnson, L.D.S., Pujanauski, L., Teodorovic, L., Jameson, S.C., Kedl, R.M., 2009. The antigen-specific CD8+ T cell repertoire in unimmunized mice includes memory phenotype cells bearing markers of homeostatic expansion. J Exp Med 206, 435–448. https://doi.org/10.1084/jem.20081829

Hataye, J., Moon, J.J., Khoruts, A., Reilly, C., Jenkins, M.K., 2006. Naive and memory CD4+ T cell survival controlled by clonal abundance. Science 312, 114–116. https://doi.org/10.1126/science.1124228

Hor, J.L., Schrom, E.C., Wong-Rolle, A., Vistain, L., Shang, W., Dong, Q., Zhao, C., Jin, C., Germain, R.N., 2025. Inhibitory PD-1 axis maintains high-avidity stem-like CD8+ T cells. Nature 1–11. https://doi.org/10.1038/s41586-025-09440-x

Hussain, T., Nguyen, A., Daunt, C., Thiele, D., Pang, E.S., Li, J., Zaini, A., O'Keeffe, M., Zaph, C., Harris, N.L., Quinn, K.M., La Gruta, N.L., 2023. Helminth Infection–Induced Increase in Virtual Memory CD8 T Cells Is Transient, Driven by IL-15, and Absent in Aged Mice. The Journal of Immunology 210, 297–309. https://doi.org/10.4049/jimmunol.2200316

Hussain, T., Nguyen, A., Thiele, D., Kannangara, D., Huang, Z., Pang, E.S., Kirn, A., Bedoui, S., Good-Jacobson, K.L., O'Keeffe, M., Quinn, K.M., La Gruta, N.L., 2025. LCMV-mediated loss of virtual memory CD8 T cells yields a functionally enhanced T cell subset. iScience 28, 113893. https://doi.org/10.1016/j.isci.2025.113893

Hussain, T., Quinn, K.M., 2019. Similar but different: virtual memory CD8 T cells as a memory-like cell population. Immunology & Cell Biology 97, 675–684. https://doi.org/10.1111/imcb.12277

Kooshesh, K.A., Foy, B.H., Sykes, D.B., Gustafsson, K., Scadden, D.T., 2023. Health Consequences of Thymus Removal in Adults. N Engl J Med 389, 406–417. https://doi.org/10.1056/NEJMoa2302892

Kousa, A.I., Jahn, L., Zhao, K., Flores, A.E., Acenas, D., Lederer, E., Argyropoulos, K.V., Lemarquis, A.L., Granadier, D., Cooper, K., D'Andrea, M., Sheridan, J.M., Tsai, J., Sikkema, L., Lazrak, A., Nichols, K., Lee, N., Ghale, R., Malard, F., Andrlova, H., Velardi, E., Youssef, S., Burgos da Silva, M., Docampo, M., Sharma, R., Mazutis, L., Wimmer, V.C., Rogers, K.L., DeWolf, S., Gipson, B., Gomes, A.L.C., Setty, M., Pe'er, D., Hale, L., Manley, N.R., Gray, D.H.D., van den Brink, M.R.M., Dudakov, J.A., 2024. Age-related epithelial defects limit thymic function and regeneration. Nat Immunol 25, 1593–1606. https://doi.org/10.1038/s41590-024-01915-9

Kwon, H., Schafer, J.M., Song, N.-J., Kaneko, S., Li, A., Xiao, T., Ma, A., Allen, C., Das, K., Zhou, L., Riesenberg, B., Chang, Y., Weltge, P., Velegraki, M., Oh, D.Y., Fong, L., Ma, Q., Sundi, D., Chung, D., Li, X., Li, Z., 2022. Androgen conspires with the CD8+ T cell exhaustion program and contributes to sex bias in cancer. Science Immunology 7, eabq2630. https://doi.org/10.1126/sciimmunol.abq2630

Masters, A.R., Hall, A., Bartley, J.M., Keilich, S.R., Lorenzo, E.C., Jellison, E.R., Puddington, L., Haynes, L., 2019. Assessment of Lymph Node Stromal Cells as an Underlying Factor in Age-Related Immune Impairment. J Gerontol A Biol Sci Med Sci 74, 1734–1743. https://doi.org/10.1093/gerona/glz029

Mittelbrunn, M., Kroemer, G., 2021. Hallmarks of T cell aging. Nat Immunol 22, 687–698. https://doi.org/10.1038/s41590-021-00927-z

Mold, J.E., Réu, P., Olin, A., Bernard, S., Michaëlsson, J., Rane, S., Yates, A., Khosravi, A., Salehpour, M., Possnert, G., Brodin, P., Frisén, J., 2019. Cell generation dynamics underlying naive T-cell homeostasis in adult humans. PLoS Biol 17, e3000383. https://doi.org/10.1371/journal.pbio.3000383

Moses, C.T., Thorstenson, K.M., Jameson, S.C., Khoruts, A., 2003. Competition for self ligands restrains homeostatic proliferation of naive CD4 T cells. Proceedings of the National Academy of Sciences 100, 1185–1190. https://doi.org/10.1073/pnas.0334572100

Moskowitz, D.M., Zhang, D.W., Hu, B., Le Saux, S., Yanes, R.E., Ye, Z., Buenrostro, J.D., Weyand, C.M., Greenleaf, W.J., Goronzy, J.J., 2017. Epigenomics of human CD8 T cell differentiation and aging. Sci. Immunol. 2, eaag0192. https://doi.org/10.1126/sciimmunol.aag0192

Nimanong, S., Ostroumov, D., Wingerath, J., Knocke, S., Woller, N., Gürlevik, E., Falk, C.S., Manns, M.P., Kühnel, F., Wirth, T.C., 2017. CD40 Signaling Drives Potent Cellular Immune Responses in Heterologous Cancer Vaccinations. Cancer Res 77, 1918–1926. https://doi.org/10.1158/0008-5472.CAN-16-2089

Ostroumov, D., Benne, N., Lozano Vigario, F., Escalona-Rayo, O., Dodz, K., Sauer, S., Suhl, L.L., Wedemeyer, H.H., Kühnel, F., Slütter, B., Wirth, T.C., 2025. Sequential STING and CD40 agonism drives massive expansion of tumor-specific T cells in liposomal peptide vaccines. Cell Mol Immunol 22, 150–160. https://doi.org/10.1038/s41423-024-01249-4

Quinn, K.M., Fox, A., Harland, K.L., Russ, B.E., Li, J., Nguyen, T.H.O., Loh, L., Olshanksy, M., Naeem, H., Tsyganov, K., Wiede, F., Webster, R., Blyth, C., Sng, X.Y.X., Tiganis, T., Powell, D., Doherty, P.C., Turner, S.J., Kedzierska, K., La Gruta, N.L., 2018. Age-Related Decline in Primary CD8+ T Cell Responses Is Associated with the Development of Senescence in Virtual Memory CD8+ T Cells. Cell Rep 23, 3512–3524. https://doi.org/10.1016/j.celrep.2018.05.057

Salazar-Cavazos, E., Jia, D., Missolo-Koussou, Y., Kenet, A.L., Achar, S.R., Dada, H., Kondo, T., Krishnan, A., Taylor, N., Klemen, N.D., Jiang, P., Waterfall, J.J., DeVoe, D.L., Altan-Bonnet, G., 2026. Stochasticity in cancer immunotherapy stems from rare but functionally critical Spark T cells. Cell 189, 1074-1085.e23. https://doi.org/10.1016/j.cell.2025.12.026

Sosinowski, T., White, J.T., Cross, E.W., Haluszczak, C., Marrack, P., Gapin, L., Kedl, R.M., 2013. CD8α+ Dendritic Cell Trans Presentation of IL-15 to Naive CD8+ T Cells Produces Antigen-Inexperienced T Cells in the Periphery with Memory Phenotype and Function. The Journal of Immunology 190, 1936–1947. https://doi.org/10.4049/jimmunol.1203149
Thompson, H.L., Smithey, M.J., Uhrlaub, J.L., Jeftić, I., Jergović, M., White, S.E., Currier, N., Lang, A.M., Okoye, A., Park, B., Picker, L.J., Surh, C.D., Nikolich-Žugich, J., 2019. Lymph nodes as barriers to T-cell rejuvenation in aging mice and nonhuman primates. Aging Cell 18, e12865. https://doi.org/10.1111/acel.12865
Turner, V.M., Mabbott, N.A., 2017. Structural and functional changes to lymph nodes in ageing mice. Immunology 151, 239–247. https://doi.org/10.1111/imm.12727
White, J.T., Cross, E.W., Burchill, M.A., Danhorn, T., McCarter, M.D., Rosen, H.R., O’Connor, B., Kedl, R.M., 2016. Virtual memory T cells develop and mediate bystander protective immunity in an IL-15-dependent manner. Nat Commun 7, 11291. https://doi.org/10.1038/ncomms11291
White, J.T., Cross, E.W., Kedl, R.M., 2017. Antigen-inexperienced memory CD8+ T cells: where they come from and why we need them. Nat Rev Immunol 17, 391–400. https://doi.org/10.1038/nri.2017.34
Yang, X., Wang, X., Lei, L., Sun, L., Jiao, A., Zhu, K., Xie, T., Liu, H., Zhang, X., Su, Y., Zhang, C., Shi, L., Zhang, D., Zheng, H., Zhang, J., Liu, X., Wang, X., Zhou, X., Sun, C., Zhang, B., 2020. Age-Related Gene Alteration in Naïve and Memory T cells Using Precise Age-Tracking Model. Front Cell Dev Biol 8, 624380. https://doi.org/10.3389/fcell.2020.624380
